# Supplementary material for: Unmasking the perching effect of the pioneer Mediterranean dwarf palm Chamaerops humilis L
Source: PLoS One. 2022 Aug 23;17(8):e0273311. doi: 10.1371/journal.pone.0273311 (PMC9398033; doi:10.1371/journal.pone.0273311)
Supplement: S1 File — Technical details concerning the cluster analysis. (DOCX) [file pone.0273311.s001.docx]

**S1 File. Cluster Analysis.** Technical details concerning the cluster analysis.

We carried out some cluster analysis to quantify the *C. humilis* distribution and level of aggregation in each study plot. To evaluate the fit of the observed point patterns in both the early and the late-successional study plots to different cluster processes, four different summary functions were used: pair correlation function *g*(r), *L*-function *L*(r), Spherical contact distribution *H_S_*(r) and nearest neighbor distribution function *D*(r). Both *g*(r) and *L*(r) functions can be calculated for the Thomas cluster point processes and are used to fit their parameters, being *g*(r) more sensitive to clustering at small scales and *L*(r) at larger ones. *H_S_*(r) and *D*(r) functions are supplementary to the previous ones, since they get different kinds of spatial information such as areas with low density of points, isolated points.

In both study plots, the four summary functions showed a good fit with the selected null models, being the model-predicted values mostly confined within the simulation envelopes (S1 Figure 1). Specifically, palm distribution in both plots were best described by a double-clustered component pattern with a random component pattern (S1 Table 1), with the late-successional study plot having a greater proportion of *C. humilis* in isolated pattern (22%) than the early-successional study plot (9%).
