# Supplementary material for: Unmasking the perching effect of the pioneer Mediterranean dwarf palm Chamaerops humilis L
Source: PLoS One. 2022 Aug 23;17(8):e0273311. doi: 10.1371/journal.pone.0273311 (PMC9398033; doi:10.1371/journal.pone.0273311)
Supplement: S2 File — (DOCX) [file pone.0273311.s002.docx]

**S2 File. Spatial analysis excluding *Rubus*’ seeds**

Not all seeds are dispersed equally due to their size and the number of them contained in the fruit, especially *Rubus*’ seeds which stands out for their small size and for being dispersed in large numbers since a single fruit contains many seeds. This contrast with the rest of dispersed seed species, which are larger in size and generally with just one seed per fruit. Therefore, we carried out some analysis excluding *Rubus’* seeds to find whether a different pattern of seed dispersal would be found.

The analysis carried out where the same explained in the Spatial Analysis within the Material and methods section. For the aggregation of *C. humilis* receiving dispersed seeds within the overall pattern of *C. humilis* (presence of seeds) the following analysis were carried out: (*i*) *p*_11_(r), (*ii*) *p*_12_(r) and (*iii*) *g*_1,1+2_ (r) - *g*_2,1+2_ (r). For the association of the number of dispersed seeds on *C. humilis* the following analysis were carried out: (*i*) r-mark correlation function km_1_. (r), (*ii*) Schlather’s correlation function and (*iii*) density correlation function.

As it is shown in the S2 Fig, the results obtained are pretty similar to the analysis obtained when *Rubus’* seeds were included in the analysis, so we can conclude that they do not interfere with the seed dispersal spatial patterns found in the late-successional study plot.
