# Supplementary material for: Unmasking the perching effect of the pioneer Mediterranean dwarf palm Chamaerops humilis L
Source: PLoS One. 2022 Aug 23;17(8):e0273311. doi: 10.1371/journal.pone.0273311 (PMC9398033; doi:10.1371/journal.pone.0273311)
Supplement: S1 Table — (DOCX) [file pone.0273311.s003.docx]

**S1 Table**. **Summary of the results of the *C. humilis* distribution fitted with the Thomas cluster process**.

|  |  |  |  |  | **LARGE CLUSTERS** | | | | **SMALL CLUSTERS** | | |  |  |
| --- | --- | --- | --- | --- | --- | --- | --- | --- | --- | --- | --- | --- | --- |
|  | N | N_isol_ | PC | PC^2^ | Aρ_1_ | σ_sum_ | 2σ_1_(m) | μ_1_ | Aρ_2_ | 2σ_2_(m) | μ_2_ | ρ_2_/ρ_1_ | σ_1_/σ_2_ |
| **Early-sucessional plot** | 109 | 10 | 0.91 | 0.82 | 15.17 | 9.15 | 8.33 | 7.19 | 65.06 | 3.78 | 1.68 | 4.29 | 2.20 |
| **Late-sucessional plot** | 180 | 40 | 0.78 | 0.60 | 7.08 | 9.15 | 8.33 | 25.41 | 28.12 | 3.78 | 6.40 | 3.97 | 2.20 |

N: number of *C. humilis* individuals. N_isol_: number of *C. humilis* individuals in isolated pattern. P_C_: proportion of *C. humilis* individuals in isolated pattern. Aρ_1_: number of large clusters in the study plot. 2σ_1_(m): size of large clusters. μ_1_: average number of individuals in one large cluster. Aρ_2_: number of small clusters in the study plot. 2σ_2_(m): size of small clusters. μ_2_: average number of individuals in one small cluster. ρ_2_/ρ_1_: average number of small clusters in one large cluster. σ_1_/σ_2_: size of large clusters relative to the size of small clusters.
