# Supplementary material for: Unmasking the perching effect of the pioneer Mediterranean dwarf palm Chamaerops humilis L
Source: PLoS One. 2022 Aug 23;17(8):e0273311. doi: 10.1371/journal.pone.0273311 (PMC9398033; doi:10.1371/journal.pone.0273311)
Supplement: S3 Table — A General Linear Model (GLM) was carried out with the interaction between C. humilis Height (m) and Area (m2) as a new variable instead of the C. humilis Volume (m3). The results found are pretty similar to the ones obtained by using the variable Volume (m3) in both study plots. Thus, we decided to maintain the initial model. (DOCX) [file pone.0273311.s005.docx]

**S3 Table. GLM carried out to analyze the palm traits in the late-successional plot.** A General Linear Model (GLM) was carried out with the interaction between *C. humilis* Height (m) and Area (m2) as a new variable instead of the *C. humilis* Volume (m3). The results found are pretty similar to the ones obtained by using the variable Volume (m3) in both study plots. Thus, we decided to maintain the initial model.

| **Explanatory variable** | **Competing models** |  | **β** | **SE** | **P** | **AICc** | **ΔAIC** |
| --- | --- | --- | --- | --- | --- | --- | --- |
| ***Model 1*** |  |  |  |  |  |  |  |
| Presence of Bird Feces | Gender + Area (m^2^) + Richness |  |  |  |  | 196.63 | 0 |
|  |  | Intercept* | -0.77559 | 0.30364 | 0.010641 |  |  |
|  |  | Gender (Male) | 0.55831 | 0.35223 | 0.112956 |  |  |
|  |  | Area (m^2^)* | 0.0891 | 0.02394 | 0.000198 |  |  |
|  |  | Richness | -0.4846 | 0.26617 | 0.068635 |  |  |
|  | Gender + Height (m) + Area (m2) + Richness |  |  |  |  | 198.56 | 1.93 |
|  | Gender + Height (m) + Area (m^2^) + Height*Area + Richness |  |  |  |  | 199.49 | 2.86 |
|  | Gender + Height (m) + Area (m^2^) + Height*Area + Richness + Beneficiary height (cm) |  |  |  |  | 201.76 | 5.13 |
| Deviance = 188.63 |  |  |  |  |  |  |  |
| ***Model 2*** |  |  |  |  |  |  |  |
| Presence of Dispersed Seeds | Height (m) + Richness |  |  |  |  | 137.98 | 0 |
|  |  | Intercept* | -3.3834 | 0.5486 | 6.93E-10 |  |  |
|  |  | Height (m) | 0.4797 | 0.2728 | 0.07872 |  |  |
|  |  | Richness* | 1.9713 | 0.7313 | 0.00703 |  |  |
|  | Height (m) + Area (m2) + Richness |  |  |  |  | 139.96 | 1.98 |
|  | Height (m) + Area (m^2^) + Height*Area + Richness |  |  |  |  | 141.64 | 3.66 |
|  | Gender + Height (m) + Area (m^2^) + Height*Area + Richness |  |  |  |  | 143.5 | 5.52 |
|  | Gender + Height (m) + Area (m^2^) + Height*Area + Richness + Beneficiary height (cm) |  |  |  |  | 147.44 | 9.46 |
| Deviance = 131.98 |  |  |  |  |  |  |  |
| ***Model 3*** |  |  |  |  |  |  |  |
| Number of Dispersed Seeds | Gender + Height (m) + Area (m^2^) + Height*Area + Richness + Beneficiary height (cm) |  |  |  |  | 1273.09 | 0 |
|  |  | Intercept* | -2.8228 | 1.07845 | 0.00886 |  |  |
|  |  | Gender (Male)* | 1.80173 | 0.12685 | < 2E-16 |  |  |
|  |  | Height (m)* | -1.9455 | 0.45779 | 2.14E-05 |  |  |
|  |  | Area (m^2^)* | -0.1135 | 0.01363 | < 2E-16 |  |  |
|  |  | Height*Area | 0.13377 | 0.01437 | < 2E-16 |  |  |
|  |  | Richness* | 0.95673 | 0.0926 | < 2E-16 |  |  |
|  |  | Beneficiary height (25-100cm) | 0.7794 | 1.03393 | 0.45096 |  |  |
|  |  | Beneficiary height (>100cm)* | 2.59102 | 1.0217 | 0.01121 |  |  |
| Deviance = 1155.7 |  |  |  |  |  |  |  |

Summary of fitted parameters and models employed to analyse the presence of bird feces (model 1), the presence of dispersed seeds (model 2) and the number of dispersed seeds (model 3) on *C. humilis* in late-successional plot. Competitive models are ranked from the lowest AICc value (best model) to the highest one, being significant variables indicated with asterisks (*).
