# Supplementary material for: Unmasking the perching effect of the pioneer Mediterranean dwarf palm Chamaerops humilis L
Source: PLoS One. 2022 Aug 23;17(8):e0273311. doi: 10.1371/journal.pone.0273311 (PMC9398033; doi:10.1371/journal.pone.0273311)

**S1 Fig. Univariate cluster analysis for *Chamaerops humilis*** in the early (A, C, E, G) and late-successional study plots (B, D, F, H). (A, B) Pair correlation function *g*(r). (C, D) *L*-function *L*(r). (E, F) Spherical contact distribution *H_S_*(r). (G, H) Nearest neighbor distribution function *D*_1_(r). The expected mark connection function statistics (gray line) and the corresponding simulation envelopes (black lines), being the fifth lowest and highest values of the functions created by 199 simulations of the null model, are also shown.


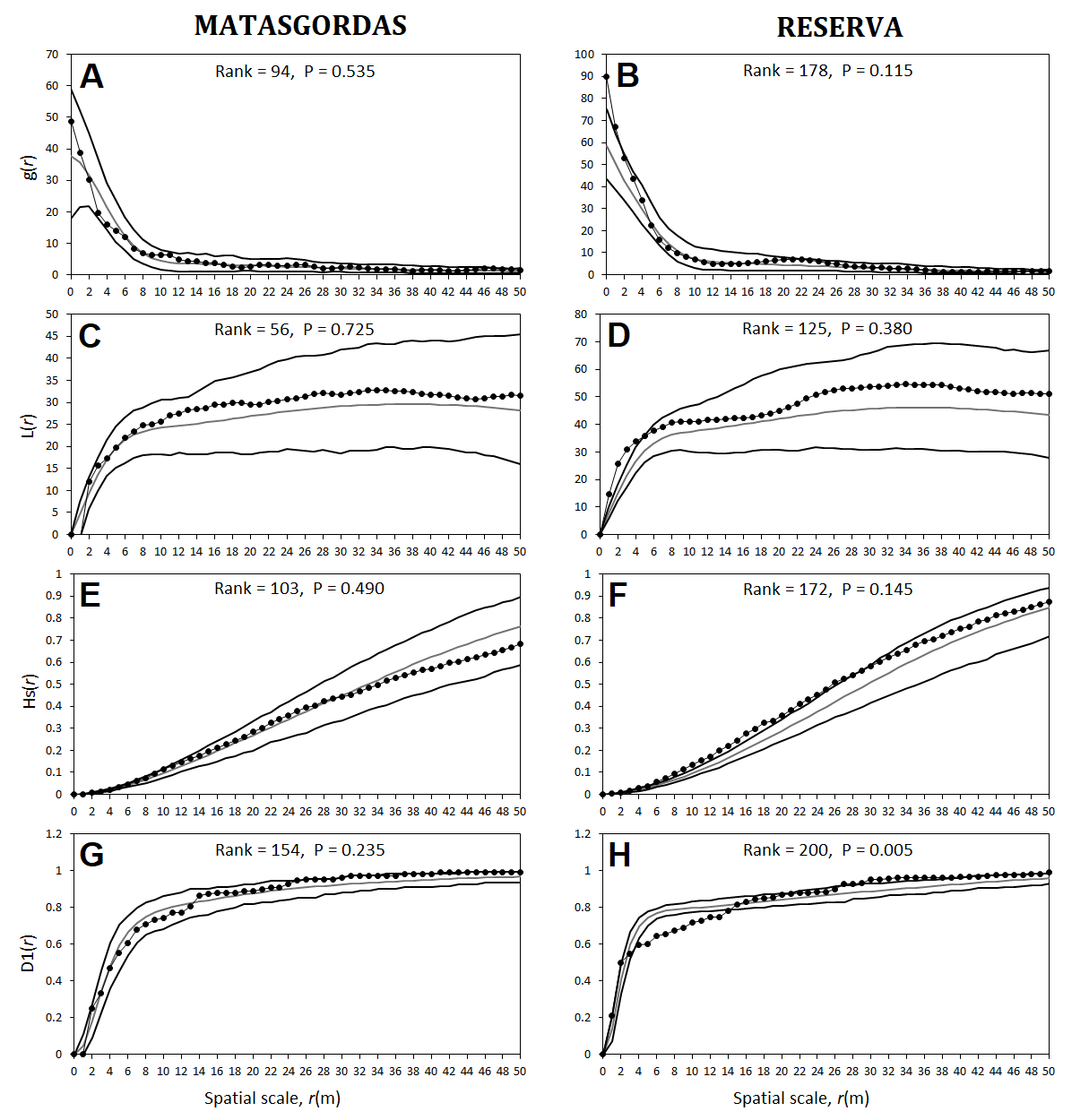

Supplement: S1 Fig — Univariate cluster analysis for Chamaerops humilis in the early (A, C, E, G) and late-successional study plots (B, D, F, H). (A, B) Pair correlation function g(r). (C, D) L-function L(r). (E, F) Spherical contact distribution HS(r). (G, H) Nearest neighbor distribution function D1(r). The expected mark connection function statistics (gray line) and the corresponding simulation envelopes (black lines), being the fifth lowest and highest values of the functions created by 199 simulations of the null model, are also shown. (DOCX) [file pone.0273311.s006.docx]
