# Supplementary material for: Unmasking the perching effect of the pioneer Mediterranean dwarf palm Chamaerops humilis L
Source: PLoS One. 2022 Aug 23;17(8):e0273311. doi: 10.1371/journal.pone.0273311 (PMC9398033; doi:10.1371/journal.pone.0273311)

**S2 Fig.** **Analysis of feces with seeds (excluding *Rubus*) in the late-successional study plot using mark connection functions as summary statistics**. (A) The mark connection function *p*_11_(*r*) gives the conditional probability that, from two *C. humilis* that are separated by distance *r*, both are type 1 (i.e., with seeds). (B) The mark connection function *p*_12_(*r*) gives the conditional probability that, from two *C. humilis* that are separated by distance *r*, the first is type 1 (i.e., with seeds) and the second is type 2 (i.e., without seeds). (C) The test statistic *g*_1,1+2_(*r*) - *g*_2,1+2_(*r*) compares the density of *C. humilis* (i.e., 1 + 2) around *C. humilis* with seeds (i.e., type 1) with the density of *C. humilis* (i.e., 1 + 2) around *C. humilis* without seeds (i.e., type 2). (D, E, F) Mark correlation functions to evaluate a potential spatial structure in the number of dispersed seeds (excluding *Rubus*). (D) The r‐mark correlation function describes the mean number of seeds (mi) on a *C. humilis* at distance r of another *C. humilis*. (E) Schlather's correlation function quantifies the correlation between the number of seeds on two different *C. humilis* separated by distance r. (F) Density correlation function assesses the correlation between the number of seeds and the number of neighbours located at a distance r. The expected mark connection function statistics (gray line) and the corresponding simulation envelopes (black lines), being the fifth lowest and highest values of the functions created by 199 simulations under random labelling, are also shown.


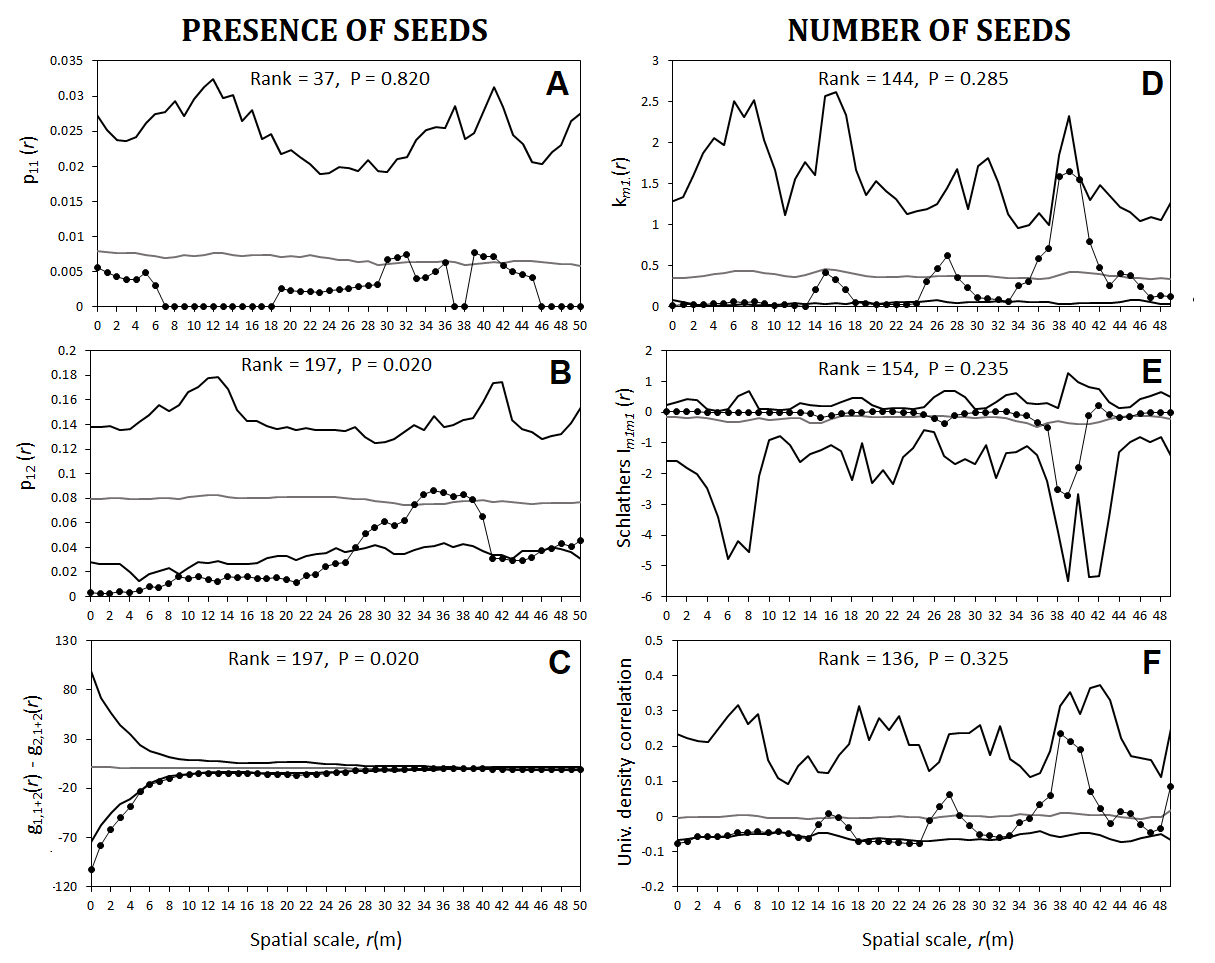

Supplement: S2 Fig — (A) The mark connection function p11(r) gives the conditional probability that, from two C. humilis that are separated by distance r, both are type 1 (i.e., with seeds). (B) The mark connection function p12(r) gives the conditional probability that, from two C. humilis that are separated by distance r, the first is type 1 (i.e., with seeds) and the second is type 2 (i.e., without seeds). (C) The test statistic g1,1+2(r)—g2,1+2(r) compares the density of C. humilis (i.e., 1 + 2) around C. humilis with seeds (i.e., type 1) with the density of C. humilis (i.e., 1 + 2) around C. humilis without seeds (i.e., type 2). (D, E, F) Mark correlation functions to evaluate a potential spatial structure in the number of dispersed seeds (excluding Rubus). (D) The r‐mark correlation function describes the mean number of seeds (mi) on a C. humilis at distance r of another C. humilis. (E) Schlather’s correlation function quantifies the correlation between the number of seeds on two different C. humilis separated by distance r. (F) Density correlation function assesses the correlation between the number of seeds and the number of neighbours located at a distance r. The expected mark connection function statistics (gray line) and the corresponding simulation envelopes (black lines), being the fifth lowest and highest values of the functions created by 199 simulations under random labelling, are also shown. (DOCX) [file pone.0273311.s007.docx]
