# Supplementary material for: Unmasking the perching effect of the pioneer Mediterranean dwarf palm Chamaerops humilis L
Source: PLoS One. 2022 Aug 23;17(8):e0273311. doi: 10.1371/journal.pone.0273311 (PMC9398033; doi:10.1371/journal.pone.0273311)
Supplement: Attachment — Submitted filename: PONE-D-22-01669_R1.pdf [file pone.0273311.s010.pdf]

PLOS ONE

Unmasking the perching effect of the pioneer Mediterranean palm <i>Chamaerops humilis</i> L.

--Manuscript Draft--

**Manuscript Number:**	PONE-D-22-01669R1
**Article Type:**	Research Article
**Full Title:**	Unmasking the perching effect of the pioneer Mediterranean palm <i>Chamaerops humilis</i> L.
**Short Title:**	Unmasking the perching effect of <i>Chamaerops humilis</i> L.
**Corresponding Author:**	Jose M. Fedriani<br>Centro de Investigaciones sobre Desertificacion<br>Moncada, Valencia SPAIN
**Keywords:**	Ecological restoration; frugivorous birds; human-disturbed habitat; mediterranean old-field; seed dispersal; recolonization; spatial point pattern analysis
**Abstract:**	Although farmlands are the most extended terrestrial biomes, the abandonment of traditional agriculture in many parts of world has brought challenge for the restoration of such human-disturbed habitats. Seed arrival is a crucial necessary ecological process during the (re)colonization that can be enhanced, by the so-called “perch plants”. Little is known about the spatial patterns of “perching” effect in disturbed habitats. To evaluate several spatial patterns of “perching” effect and whether the seed arrival via frugivorous birds is related to the spatial distribution of the perch plants, we used a spatially explicit approach in two disturbed sites within the Doñana National Park (SW Spain). Specifically, we chose as study system the pioneer Mediterranean dwarf palm <i>Chamaerops humilis</i> L., which is often used as perch by a variety of frugivorous bird species. A total of 289 <i>Chamaerops humilis</i> were sampled in search of bird feces (N = 2998) and dispersed seeds (N = 529). The found seeds belonged to six different woody species from five different families. Nine bird species from six different families were recorded using <i>Chamaerops humilis</i> as perches. GLMs analyses indicated that taller <i>Chamaerops humilis</i> males with high richness of beneficiary woody species received more dispersed seeds We detected a random spatial structure of bird feces and dispersed seeds in one site, while a nonrandom spatial structure was found in the other one, where isolated <i>Chamaerops humilis</i> received a higher number of bird feces and dispersed seeds than expected under spatial null models. The difference in spatial patterns between both study sites could relate, among other factors, to the different state of development in the ecological succession. The fact that frugivorous birds visited most often isolated <i>Chamaerops humilis</i> (rather than aggregated ones) questions the typical aggregated spatial structure of revegetation designs of many restoration projects. This study reveals novel spatial aspects of the “perching” effect which could be helpful in the restoration of human-disturbed habitats worldwide.
**Order of Authors:**	Víctor González-García
	Jose M. Fedriani
	Pedro J. Garrote
**Response to Reviewers:**	Dear Dr. Karen Root,<br>We have now prepared a substantially revised version of our manuscript "Unmasking the perching effect of the pioneer Mediterranean palm Chamaerops humilis L." (PONE-D-22-01669) submitted to Plos One. We would like to thank you and the reviewers for your thoughtful comments, and we are happy with the careful and constructive criticisms and helpful suggestions. In response to the comments, we have introduced the following three main changes:<br>1. As suggested by the editor and both reviewers, the manuscript has been carefully revised several times to improve the English grammar and flow of logic.<br>2. Following suggestion by reviewer #1, the role of birds as frugivorous and seed dispersers have been described in further detail. Also, some spatial analyses have been repeated excluding data from Rubus ulmifolius and the result are now provided in

Powered by Editorial Manager® and ProduXion Manager® from Aries Systems Corporation

a new appendix.
3. As suggested by reviewer #2, we have conducted cluster analyses to estimate the distribution of C. humilis in both study plots. Also, we fitted a new GLM to estimate the effect of the interaction between palm height and palm area on seed arrival to C. humilis. Results of these new analyses are provided in the corresponding new appendices.
Please, find below an itemized list detailing how we have responded to each of the items in the reports. The comments are in black font, our response follows in blue font, and changed phrases in response to the comment are provided in blue font and between quotation marks and.
Thank you again for your kind assistance and the time devoted to our manuscript.
Sincerely,

V.

1. Changes made to address editor comments:
The discussion, though, needs substantial revision to increase clarity and improve the interpretation of your results, especially in terms of language. It contains many awkwardly worded sentences that are challenging to understand (e.g., lines 459-462; 465-467; 493-497; 508-511; 550-552; 565-566; 594-596) and/or use poor grammar (e.g., lines 268-269; 502-503; 536-537; 559-561). Some of these issues could be minimized if you use more active rather than passive voice.
Thank you for your suggestions. To address them, we made the following changes:
Lines 495-499 (previously 459-462): "Considering that our sampling period did not take place throughout the year and that an unknown fraction of the bird feces presents in target C. humilis individuals surely went unnoticed, the data obtained could be considered as a hint of how widespread the "perching" effect in both study plots. Therefore, the high presence of (frugivorous) bird feces and dispersed seeds in both study plots is remarkable.".
Lines 501-504 (previously 465-467): "Nevertheless, we did not find seeds of the beneficiary species C. salviifolius, P. bourgaeana, Q. suber and S. genistoides, which are usually associated to C. humilis. Most of these plant species are seldom dispersed by birds".
Lines 516-519 (previously 493-497): "However, by concentrating frugivorous activity to aggregated planted trees, seed dispersal could be limited [16, 55]. Therefore, our results supporting the role of isolated perches diverge from the most widespread tendency in ecological restoration of aggregating trees and shrubs during revegetation efforts [65]".
Lines 523-526 (previously 508-511): "However, by concentrating frugivorous activity to aggregated planted trees, seed dispersal could be limited [16, 55]. Therefore, our results supporting the role of isolated perches diverge from the most widespread tendency in ecological restoration of aggregating trees and shrubs during revegetation efforts [65]".
Lines 583-587 (previously 550-552): "Additionally, beyond the fact of bearing fleshy fruits, the own beneficiary species could be used as a perch and thus attract birds. Therefore, a similar reasoning explained earlier about how C. humilis height influences seed dispersal, could be applied for the revealed positive effect of height of beneficiary plants".
Line 605-607: (previously 565-566): "In the last 30 years, restoration ecology has undergone considerable development thanks to the emerging new tools and techniques that facilitate the retrieval of biological properties of disturbed habitats [80]".
Lines 635-638 (previously 594-596): "We prove for the first time its key role as natural perch, an ecological function that, though it is shared with some fleshy-fruited woody plants, is especially relevant in early stages of ecological succession when few pioneer species occur".
Lines 288-289 (previously 268-269): "Finally, if km1. (r) ~ 1, indicates that the number of seeds is not affected by the distance to C. humilis individuals".
Lines 531-533 (previously 502-503): "Besides, it seems reasonable to expect that birds select C. humilis located on the edge of the plot, since these individuals are closer to the Mediterranean scrubland and thus allow birds to move between habitats with a minimal consumption of energy".
Previous lines 536-537: deleted.
Lines 598-600 (previously 559-561): "In summary, taller (and isolated) male C. humilis with a higher diversity of beneficiary species seemed to be the most suitable for

Powered by Editorial Manager® and ProduXion Manager® from Aries Systems Corporation

	attracting frugivorous birds and, therefore, received higher number of dispersed seeds".<br>In particular, it will be important to address the concerns of the both of the reviewers who highlight a number of areas that need further exploration. Reviewer #1 has some concerns about the foraging and movement ecology of the dispersers and the potential impact on the seed dispersal patterns as well as the large differences in the types of seeds potentially dispersed. Please also note that Reviewer #1 has some specific questions that should be addressed to improve clarity, particularly in the study design and motivations. Reviewer #2 also provides some valuable recommendations for each section and identifies a number of places where more explanation or revision is needed. Some additional issues are identified below:<br>Regarding Reviewer #1's concerns: we have improved our manuscript according to the comments received. We answered the questions related to the ecology of dispersers and did some new analyses without considering the Rubus' seeds. We found that the results obtained when we excluded these seeds barely differed from the results obtained when those seed were included.<br>In respect of Reviewer #2's comments, we have considerably improved the clarity and readability of the whole manuscript. We also added some information that Reviewer #2 considered important to clarify some aspects (C. humilis distribution, replicability, area of study, origin and identification of feces, etc.). We also run a new GLM including the interaction between C. humilis height (m) and area (m2) instead of the variable volume (m3), as suggested. The GLM obtained for both study plots (Matasgordas an Reserva) and for the three studied levels (presence of feces, presence of seeds and number of seeds) were pretty similar to the models obtained when the variable Volume was used. So, we provide the new model in the appendixes and kept the original one in the main text. We also carried out the analysis to study the distribution and level of aggregation of C. humilis. Our cluster analyses showed that Reserva has a greater percentage of isolated C. humilis, which is consistent with our results showing that birds prefer isolated C. humilis for perching in Reserva.<br>Line 63: "template" should be "temperate"<br>Thank you. Done.<br>Line 99: The use of the term "beneficiary" requires some type of description/definition or a reference source.<br>To address your comment have made the following change at Line 79-81:<br>"Interestingly, in some cases, the same shrub species acting as perches also act as nurse plants, i.e. facilitating the emergence, growth and survival of other plant species, [23] which are designated as "beneficiary species", promoting the natural (re)colonization [24, 25]."<br>Also, at Line 220-222, we now state: "Beneficiary species were woody plants that grow under dwarf palms and emerge from the top of their surface. These species often benefit from C. humilis due to microclimatic improvement and protection against herbivory [42]."<br><br>What characteristics define this group and is it context-dependent?<br>Thank you for such an interesting point. In Garrote et al (2019), some of us proved that there is a consistent and strong positive spatial association between C. humilis and several woody species. This result suggested that the interaction is constant rather than context dependent. Most recently (Garrote et al. 2022), however, we have showed through field experiments that the strength and even the sign of such interaction changes mostly with plant stages (seed survival, seedling emergence and survival), which would suggest context-dependency. However, the overall effect C. humilis on woody species was always either positive or neutral and whenever we detected neutral effects that result seemed associated to limited sample sizes. So, most compelling evidence indicate that, in overall, C. humilis exerts a consistent positive effect on woody species.<br>Garrote PJ, AR Castilla, JM Fedriani. 2019. The endemic Mediterranean dwarf palm boosts old-field recolonization: implications for restoration. Journal of Environmental Management 250: 109478.<br>Garrote PJ, A Castilla, JM Fedriani. 2022. Coping with changing plant-plant interactions in restoration ecology: effect of species, site, and individual variation. Applied Vegetation Sciences. DOI: 10.1111/avsc.12644<br>Line 232: Why 199 simulations? It seems like a rather arbitrary number. Is this based on some initial modeling or preliminary results?

The 199 simulations are the number recommended by Wiegand & Moloney (2013). They estimate the curve asymptotes for each spatial statistic (rank, P-value, etc.). Wiegand, T., & Moloney, K. A. (2013). Handbook of spatial point-pattern analysis in ecology. CRC press.
Line 255: “that” should be “than”
Thank you. Done.

Journal Requirements:
When submitting your revision, we need you to address these additional requirements.
1. Please ensure that your manuscript meets PLOS ONE's style requirements, including those for file naming.
Reviewed and ensured.
2. In your Methods section, please provide additional information regarding the permits you obtained for the work. Please ensure you have included the full name of the authority that approved the field site access and, if no permits were required, a brief statement explaining why.
We have added at Lines 131-132: “All permits necessary were granted by the National Park Service (ref. 2019/10) and the Junta de Andalucía (ref. 2019107300002261/IRM/MDCG/mes).”
3. Please note that in order to use the direct billing option the corresponding author must be affiliated with the chosen institute. Please either amend your manuscript to change the affiliation or corresponding author, or email us at plosone@plos.org with a request to remove this option.
Thank you. We have now specified JM Fedriani (who is affiliated at CSIC) as corresponding author in the Plos One manuscript submission platform. Also, in the manuscript both JM Fedriani and V Gonzalez-Garcia appear as corresponding authors.
4. We note that you have stated that you will provide repository information for your data at acceptance. Should your manuscript be accepted for publication, we will hold it until you provide the relevant accession numbers or DOIs necessary to access your data. If you wish to make changes to your Data Availability statement, please describe these changes in your cover letter and we will update your Data Availability statement to reflect the information you provide.
https://figshare.com/articles/dataset/PerchigEffectDwarfPalm_xlsx/19642446
5. We note that Figures 1 and 4 in your submission contain map images which may be copyrighted. All PLOS content is published under the Creative Commons Attribution License (CC BY 4.0), which means that the manuscript, images, and Supporting Information files will be freely available online, and any third party is permitted to access, download, copy, distribute, and use these materials in any way, even commercially, with proper attribution. For these reasons, we cannot publish previously copyrighted maps or satellite images created using proprietary data, such as Google software (Google Maps, Street View, and Earth). For more information, see our copyright guidelines: http://journals.plos.org/plosone/s/licenses-and-copyright.
Sorry, we are not sure to fully understand your concern. Figure 1 and Figure 4 represent study areas sketch maps and study plots, respectively. They have been made by us specifically for this manuscript. To this aim, we used QGIS v3.18 software. So, we don’t see any copyright issue. However, we now provide the photo credit in Figure 2 and Figure 3 headings (Photo credit: Pedro J. Garrote) which was missing in the earlier manuscript version.

2. Changes made to address reviewer #1 comments:
Although there is a lot of consideration on the use of artificial perches in restoration, the role of natural perches deserves more investigation, especially in a poorly studied biome under this perspective, as is the case with Mediterranean Europe. In this regard, the authors provide an important dataset based on field observations and fine spatial analyses, which represents a great contribution to the restoration of that particular habitat.
Thank you very much for your suggestions and comments to improve our manuscript.

Despite their efforts to explain the different patterns found between the two study sites (Mastasgordas and Reserva), I think this attempt was a bit confused, perhaps because there may be underlying causes not fully investigated by the authors. I would put this question in the perspective of the field data and not in the analysis itself. One underlying problem may be the unbalanced frequency of birds and seeds in the two

areas. From the nine bird species recorded, six are reputed insectivores and just three can be considered omnivores that can take significant amounts of fruit in their diets (https://esajournals.onlinelibrary.wiley.com/doi/abs/10.1890/13-1917.1). These three omnivore species performed more than 50% of the visits recorded in the two areas and probably deposited more seeds per visit beneath the palms than the other ones. Special attention should be paid to the foraging and movement patterns of these three omnivores, since they may account for the general pattern of the seed rain and, perhaps, provide sound explanations for these patterns.

Lines 190-193: “Overall, nine bird species were recorded perching: Cisticola juncidis, Lanius senator, Phoenicurus ochruros, Phylloscopus collybita, Pica pica, Saxicola rubicola, Sturnus unicolor, Sylvia atricapilla and Sylvia melanocephala”.

All species but Cisticola, Lanius, Pica and Sturnus are reported by Herrera (1984) as frugivorous. However, Pica pica does include fleshy-fruits (Green et al. 2019) and acorns (Martínez-Baroja 2019) in their diet. Additionaly, genus Sturnus has been reported as fruit-eater too (Jordano 1987, Gonzalez-Varo et al. 2017), as well as genus Phoenicurus, Sylvia and Saxicola (Campo-Celada et al. 2022, Carnicer et al. 2009). Therefore, just two of our reported species (Cisticola juncidis and Lanius senator) would not participate directly in seed dispersal.

That is a good point even though during the fall and early winter (when our study was carried out) most of the recorded bird species (Table 1) shift their diet, at least partially, towards a frugivorous diet. To address your concern, we have added the following text in the discussion (Line 562-570): “However, we sampled during the seed dispersal season, time when some birds change their diet into a more fruit-based one. For instance, it has been reported that P. ochruros, S. atricapilla and S. malenocephala are able to quickly change their diet when fleshy fruits become available, while other species such as S. rubicola show a more gradual change to a frugivorous diet [67, 68]. Also, S. unicolor, P. ochruros, P. collybita, P. pica, and S. rubicola has been reported as fruit-eater, although in lesser quantities [69, 70, 71, 72, 73]. Therefore, although not all recorded bird species contribute equally to seed dispersal, fleshy fruits represent a considerable fraction of most of their diets during the seed dispersal season.”

Campo-Celada M, Jordano P, Benítez-López A, Gutiérrez-Expósito C, Rabadán-González J, Mendoza I. 2022. Assessing short and long-term variations in diversity, timing and body condition of frugivorous birds. Oikos, 2: e08387

Carnicer J, Jordano P, Melían CJ. 2009. The temporal dynamics of resource use by frugivorous birds: a network approach. Ecology, 90(7): 1958-1970.

Gonzalez-Varo JP, Carvalho CS, Arroyo JM, Jordano P. 2017. Unravelling seed dispersal through fragmented landscapes: Frugivore species operate unevenly as mobile links. Molecular Ecology, 26(16): 4309-4321.

Green AJ, Elmberg J, Lovas-Kiss A. 2019. Beyond Scatter-Hoarding and Frugivory: European Corvids as Overlooked Vectors for a Broad Range of Plants. Front. Ecol. Evol., 18.

Herrera CM. 1987. A study of avian frugivores, bird-dispersed plants, and their interaction in Mediterranean scrublands. Ecological Monographs, 54(1): 1-23

Martínez-Baroja L, Pérez-Camacho L, Villar-Salvador P, Rebollo S, Quiles P, Gómez-Sánchez D, Molina-Morales M, Leverkus AB, Castro J, Rey-Benayas JM. 2019. Massive and effective acorn dispersal into agroforestry systems by an overlooked vector, the Eurasian magpie (Pica pica). Ecosphere, 10(12): e02989.

Another point addresses the plant composition and fruit types between the two areas. Rubus ulmifolius is the only recorded fruit consumed that is a functional berry, containing dozens of seeds. The other plant species are single-seeded drupes. This species was not related as common or abundant in Matasgordas, whose seed rain was nil, but its presence was highlighted in Reserva, whose seed rain under C. humilis accounted for 85-90% of the total seed rain. Even a few feces with dozen of R. ulmifolius seeds would certainly bias the results and the comparison between areas when the response variable “number of seeds” is considered. Would the spatial patterns be the same between the areas if R. ulmifolius is excluded from the analysis? If so, the conclusions presented by the authors should be revisited and adjusted to the bias introduced by this species.

I see these points do not invalidate the importance of C. humilis as perches and facilitative species in that habitat, but at least would provide more explanatory power for the distinct spatial patterns found between the two study sites.

Thank you for your suggestion. We repeated our spatial analysis for the presence of seeds and for the number of seeds excluding Rubus’ seeds. The results obtained are pretty similar to the ones obtained initially when we included those seeds in the

	analysis. The results from such a new analysis are now included as Supporting Information (S2 Spatial seed analysis excluding Rubus' seeds).<br>Besides that, I would recommend the authors to improve the English text, as some sentences are somewhat difficult to understand.<br>Thank you. We have carefully revised the manuscript several times to improve it language use and clarity. The revised manuscript has considerably improved in this respect.<br>Bellow, I add some other specific points to the authors.<br>Page 4, lines 94-96: To question whether there are differences in the role of C. humilis as perches between the two areas presupposes some previous knowledge of the distributional pattern of this species between the areas. Although the authors provide a detailed description of the study areas in the proper session (pages 6 and 7) it should be enlightening to give a hint here on what triggered the idea that the plant role could be different between areas. Certainly, these two areas were not chosen at random.<br>Garrote et al. 2019 documented that Reserva presents higher density of shrubs than our study plot at Matasgordas. Such a difference in shrub cover led to a greater abundance of fleshy fruits in Reserva which likely makes this site more attractive to frugivorous birds.<br>Lines 137-138: "Study areas are different in terms of vegetation and human-use history [4, 38]".<br>Lines 147-152: "Due to such past human activity, Matasgordas is now formed, mainly, by two habitats: (i) Scrubland dominated by P. lentiscus shrubs with some Q. suber and O. europaea var. sylvestris trees [4, 33] and (ii) an old-field, where our study plot was set, which woody vegetation is mainly composed by animal-dispersed native plants such as C. humilis, P. lentiscus, D. gnidium and P. bourgaeana, Asparagus aphyllus L., Halimium halimifolium (L.) Willk and Cistus salviifolius L. and scattered Q. suber and O. europaea var. sylvestris trees [4]".<br>Lines 167-175: "Reserva (…) has been historically managed by human for agriculture, hunting, cattle grazing and tree felling, especially O. europaea var. sylvestris and Q. suber [41]. Reserva was protected earlier than Matasgordas, in 1964 and it has been recovering ever since, being recolonised by animal-dispersed plants such as A. aphyllus, C. humilis, Phillyrea angustifolia L. or R. ulmifolius [4]. This scrubland is dominated by H. halimifolium and Stauracanthus genistoides (Brot.) Samp. with scattered trees of Q. suber, O. europaea var. sylvestris and Pinus pinea [33]. The study plot at Reserva presents higher density of shrubs than the one at Matasgordas. Such a difference in shrub cover led to a greater abundance of fleshy fruits in Reserva, which likely makes this site more attractive to frugivorous birds [4].".<br>Page 6, line 142: Delete the period after bourgaeana.<br>Thank you. Done.<br>Page 7, lines 177-178: What was the total sampling effort for bird perching for the separate areas?<br>Thank you for your comment. To address it, we have added the following text (Line 189-190) in the revised manuscript: "During the sampling, which lasted about 3h per day in Matasgordas and about 2h in Reserva, any observed bird perching on focal C. humilis individuals was recorded."<br>Page 9, lines 189-192: What do you mean by "palm interior"? Does it include the foliage surface in the interior and also the soil under the foliage? Was the open area of a similar surface associated with the palms bare soil?<br>Palm interior refers to the foliage surface since the soil under the palm was unreachable in most of the cases. Thus, we have replaced in the manuscript "palm interior" for "palm foliage surface".<br>Yes, the open area associated to each palm was of similar surface (than the associated palm) and mostly in bare soil with scarce vegetation, mainly grasses. This open area was 3-4m from the associated C. humilis.<br>Lines 200-204: "Every target C. humilis was checked for bird feces and regurgitations by carefully checking the palm foliage surface. We also evaluated whether seeds arrive at open spaces without palms. To this aim, we checked for the presence of bird feces and regurgitations in an open area associated to each focal C. humilis. These areas were of similar surface and mostly located in bare soils with scarce vegetation (mainly grasses) and 3-4m from the associated C. humilis".<br>What were the criteria used to distinguish between bird-and-mammal dispersed seeds in these areas?<br>Most sampled seeds were found within bird feces which are easily distinguishable from mammal feces based on size, shape, color and smell. For example, the uric acid in bird

Powered by Editorial Manager® and ProduXion Manager® from Aries Systems Corporation

feces forms a conspicuous and white sticky paste that makes them easily to identify. Also, the diameter of feces of target birds (a few millimeters) was much smaller than for feces of local frugivorous mammal species (several centimeters).
Page 14, lines 312-313: Since the number of feces and number of seeds are two interrelated response variables, what is the weight of the regurgitated seeds in the analysis? How much regurgitated seeds contributed to the total number of seeds sampled?
Regurgitated seeds were just a small fraction of the total sample. Their number was too small to really contribute to the final analysis.
Page 16, line 375: Consider this writing: "Otherwise, in Reserva, the probability …"
Thank you. We have changed the sentence. Line 395-397 (previously line 375): "Results in Reserva substantially differed from results from Matasgordas. The probability of two C. humilis separated by distance r, having both seeds (p11(r) statistic), was similar to expected of random labelling for all spatial scales (Fig 6B)."
Page 2, line 501: Insert "of" after "chances".
Thank you. Done.

3. Changes incorporated to address reviewer #2 comments:
Unmasking the perching effect of the pioneer Mediterranean palm Chamaerops humilis L.

I have now carefully read the manuscript. I found the authors approach very interesting using SPPA to unravel the perching effect. Using this spatial analysis they evaluate several spatial patterns of "perching" effect and whether the seed arrival via frugivorous birds is related to the spatial distribution of the C. humilis in two disturbed scenarios. I think the manuscript could be interesting will be interesting for readers interested in restoration or ecological succession drivers.
Thank you very much for your suggestions and comment to improve our manuscript.
I think the manuscript needs to improve the readability mostly in the methods and the results section (see comments below).
We have considerably improved the clarity and readability of the whole manuscript, including the methods and results sections.
I have concerns with the general work. On one hand, I think that it needs more information about the state of the selected plots. For instance, the main objective is to evaluate the perching effect based on the plants distribution, however, the authors did not show the spatial distribution of the individual plants, plants density, size of the plot, etc. that in my opinion has to be in the article.
Thank you for communicating this concern. Both Figure 1 and Figure 4 show the spatial distribution of the individual C. humilis. Following your suggestion, plant densities and plot sizes are now included in the Material and Methods section:
Line 136-137: "We selected two study plots 10 km apart called Matasgordas (area sampled: 13.9 ha) and Doñana Biological Reserve (area sampled: 21.4 ha)".
Line 182-184: "The density of C. humilis plants in Matasgordas' plot (7.78 individuals/ha) was pretty similar than in Reserva' plot (8.41 individuals/ha).".
On the other hand, your main conclusions are based on some patterns found in one plot (male plants, isolation). This is my major concern because when you present the analysis of the other plot the main patterns did not applied to other ecological context. It seems that the results only applied under certain circumstances. I think this is a very common problem when studying the effects of perching which makes it very difficult to generalize the drivers of this process.
We agree in that is very difficult to generalize the drivers of the perching effect. Differences in results between our study areas likely relate to the fact that they present a different state of ecological succession, an issue that is detailed in our Discussion and could be used by managers to decide, for example, whether particular areas are adequate to promote seed arrival using natural or artificial perches.

Abstract
L39- "dispersed seeds We detected" missed a point
Thank you. Done.
Introduction

L97: Do exist some individuals of C. humilis that act as "hotspots" of dispersed seeds?

Change for: Do any C. humilis individuals act as "hot spots" for seed dispersal?
Changed for: Do particular C. humilis individuals act as "hot spots" of seed arrival, and if so, why? (Lines 99-100, previously Line 97).
Methods

L134-144. With this explanation it is unclear where did you work.
Reading some works made in the area it seems that Matasgordas has a higher plant density than Reserva. For instance, In the article by Fedriani and Delibes (2011) when the description of the Matasgordas site is made, it seems to have a higher density of individuals, same situation in the article by Jácome et al. 2016 where the spatial patterns of this species are studied where they also report higher number of individuals in the area. Thus, it would be interesting to specify that you do it "in a low density area in order to be comparable with Reserva."
Thank you for your comment. The density for C. humilis is highly spatially heterogeneous both in Matasgordas and in Reserva areas. Thus, it is expectable that density changes depending on the specific location of the study plots in both areas. In our selected study plots, the density of C. humilis plants in Matasgordas (7.78 individuals/ha) was pretty similar to that in Reserva (8.41 individuals/ha) but, again, this could change if the plots were set in different specific locations. Ultimately, Matasgordas and Reserva were not too different in terms of palm density.
Our previous studies (Fedriani and Delibes 2011, Jácome et al. 2016) were carried out in Matasgordas and Reserva but their study plots were not the same that the pots of the present study; so, no wonder they reported other C. humilis densities. For instance, the study by Fedriani and Delibes (2011) was carried out in a patch of Mediterranean scrubland within Matasgordas area (where C. humilis density is certainly very high), whereas our present study was conducted in the old-field within Matasgordas area (where C. humilis density is much lower than in the scrubland).
To prevent possible misunderstandings in relation to this issue, we now consistently use throughout our manuscript the term study plot instead of study site.
L163- Related with my previous comment. Although Reserva was protected earlier authors have to consider that there still traditional uses in the area, thus plants in the Reserva are submitted to a high herbivorous pressure due to the presence of horses, cattle and high density of cervids. This pressure has reflected in the lower density of individuals explained above.
As mentioned above, the densities of C. humilis in both Matasgordas (7.78 ind. ha-1) and Reserva (8.41 ind. ha-1) were not too different but, they could change depending on where the study plots are located.
Suggested papers:
-Fedriani, J. M., & Delibes, M. (2011). Dangerous liaisons disperse the Mediterranean dwarf palm: Fleshy- pulp defensive role against seed predators. Ecology, 92, 304–315.
-Jácome-Flores, M. E., Delibes, M., Wiegand, T. & Fedriani, J. M. Spatial patterns of an endemic Mediterranean palm recolonizing old fields. Ecol. Evol. 6, 8556–8568 (2016).
L170-Every target C. humilis individual (109 and 180 in Matasgordas and Reserva, respectively)
Thank you. Done.
SPPA requires the delimitation of an area in which all target individuals of the study species are georeferenced. I suppose this was the case, however it is not clear in the methodology. I suggest to put more information such as density and/or no. of individuals, area sampled, etc. for each selected plot. Also this information will clarify more the differences that you found between plots.
Thank you. The study plots are shown in Fig. 1. We have added the suggested information:
Lines 136-137: "We selected two study plots 10 km apart called Matasgordas (area sampled: 13.9 ha) and Doñana Biological Reserve (area sampled: 21.4 ha)".
Lines 181-184: "Every target C. humilis (109 and 180 in Matasgordas and Reserva, respectively) was individually georeferenced with a submetric GPS Leica 1200. The density of C. humilis plants in Matasgordas' plot (7.78 individuals/ha) was pretty similar than in Reserva' plot (8.41 individuals/ha)".
Discussion

L463-L478- Reduce the length of this paragraph, there is too much mention of dispersal mechanisms of other species. This section should only focus on the ability of C. humilis to attract different dispersers including mammals.

Powered by Editorial Manager® and ProduXion Manager® from Aries Systems Corporation

	We changed the paragraph to highlight the C. humilis ability to attract seed dispersers as you suggest.<br>Lines 500-509: "Besides, it is apparent that some seeds can emerge seedling beneath C. humilis, since all seeds collected belonged to beneficiary woody species found within C. humilis. Nevertheless, we did not find seeds of the beneficiary species C. salviifolius, P. bourgaeana, Q. suber and S. genistoides, which are usually associated to C. humilis. Most of these plant species are seldom dispersed by birds. Other animals are more likely to disperse them, such as insects for C. salviifolius or S. genistoides seeds [56, 57, 58] or mammals, such as ungulates (red deer, wild boars, domestic cattle) and carnivores (Eurasias badger, red fox) for P. bourgaeana seeds [59]. Therefore, the presence of these woody species associated to C. humilis reveals that it does not attract just bird-dispersed plants but also mammal-dispersed plants, accentuating its role in habitat restoration."<br>L484-485 I suggest to the authors to do SPPA to the individual plants to unravel the spatial patterns of the "Matasgordas and Reserva C. humilis populations", this analysis could be useful to explain some of the patterns. For instance maybe the patterns of the plants in Matasgordas tend to be more random explaining your findings without any over explanation. On the other hand maybe Reserva population its characterized by more isolated plants that had enough height to function as a perch for birds.<br>Thanks for your suggestion. We carried out some cluster analysis in order to quantify the C. humilis distribution and level of aggregation. To evaluate the fit of the observed point patterns in both Matasgordas and Reserva to different cluster processes, four different summary functions were used: pair correlation function g(r), L-function L(r), Spherical contact distribution HS(r) and nearest neighbor distribution function D(r).<br>In both study plots, the four summary functions showed good fits with the selected null models, being the model-predicted values mostly confined within the simulation envelopes. The distribution of C. humilis in Matasgordas and Reserva were both best described by a double-clustered component pattern with a random component pattern, being Reserva the one with a greater proportion of isolated C. humilis individuals. This may relate to the observed tendency found in Reserva of bird's preference for isolated C. humilis, which is now mentioned in the Discussion.<br>We added the results of cluster analyses as Supporting Information (S1 Cluster Analysis) and are quoted in the description of the study plots.<br>L528-529: …while the surface of Chamaerops humilis was found significant in Reserva I suggest to change surface to cover area.<br>Thank you for your suggestion. We have removed that line in the revised version.<br>L526-534. Sometimes is difficult and confusing to follow the explanations or results because sometimes appear first Matasgordas and then Reserva. I suggest to the authors to be consistent with the appearance order through all document.<br>Thank you. The order is now consistent, as suggested.<br>L550 Change: What is more to Furthermore or Additionally<br>Thank you. Done.<br>L552- Unexpectedly, it was found that larger C. humilis (i.e. those with larger volume) were less suitable for seed arrival, at least in Reserva. -Change "it was found" to "we found"<br>Thank you for your suggestion. We have removed that line in the revised version.<br>-I suggest to the authors not use volume but instead the interaction between cover area (surface) and height. This interaction could reveal some perching preferences regarding this two variables. The variety of shapes in this palm make it difficult to standardize the volume as a "common variable" between all individuals.<br>Although this species can be highly variable in shape, we found that C. humilis in our study plots were very similar in shape, with a consistent dome shape. This uniformity made us to choose the variable 'volume'.<br>However, following your suggestion, we carried out the GLM with the interaction between Height (m) and Area (m2) instead of the variable Volume (m3) and the results obtained were pretty similar to the ones obtained in the initial GLM. Results from this new analysis are shown in the Supporting Information (S3 Effect of palm traits).<br>L554-We speculate that male C. humilis could be selected over females because they lack fruits. C. humilis fruits are too big for birds, but they are often eaten by medium-sized carnivores such as the European badger and the red fox [33]. The presence of these predators could dissuade frugivorous birds from visit female C. humilis in fruit, and thus perching more often male palms.<br>I suggest to put some information about the abundance of "potential predators" in each study area to reinforced this idea. Another explanation to this pattern (in my opinion

	more likely) is the presence of rodents and rabbits beneath the female plants. Rodents and rabbits are also highly attracted to C. humilis fruits, in this foraging process they could predate the seeds dropped in the perching process, limiting the number of seeds. In this context, the same situation could be happening in aggregated C. humilis individuals that are preferred by rodents due to high vegetation cover.<br>Thank you for your comment. On the one hand, the presence and abundance of potential predators in this area, such as red foxes and badgers, have been studied earlier (Fedriani et al. 1998, Fedriani et al. 2008). On the other hand, all seeds collected were found at a certain height at the palm leaves, being pretty much inaccessible for those mammals (especially rabbits). Further, during our intensive monitoring of C. humilis using camera traps we have not recorded ever rodent foraging on leaves at the top surface of this palm, where seeds dispersed by birds were collected.<br>L564- It seems that your results could be useful for restoration planification where the C. humilis is a very useful species. However the inconsistency of the results between areas and the unclear relation between the traits masked by the context dependency seems to be an issue to the applicability of the study. For instance the efficiency of the perching effect seems to be related to male plants, if we use this results as it comes we would be deprecated female plants that would be useful to attract super-efficient fleshy fruit dispersers such as badgers or foxes.<br>Thank you for your comment. We have included the following sentences in the revised manuscript (Lines 593-597): "The use of C. humilis in ecological restoration should follow a balance between male and female plants. Male plants would attract preferably birds that would disperse seeds of woody species present in the surrounding area, while female plants would attract medium-sized carnivores and ungulates that would mostly disperse seed of species such as P. bourgaena and C. humilis."
**Additional Information:**	
**Question**	**Response**
**Financial Disclosure**<br><br>Enter a financial disclosure statement that describes the sources of funding for the work included in this submission. Review the submission guidelines for detailed requirements. View published research articles from *PLOS ONE* for specific examples.<br><br>This statement is required for submission and **will appear in the published article** if the submission is accepted. Please make sure it is accurate.	This work was supported by the Portuguese Foundation for Science and Technology to PJG (SFRH/BD/130527/2017) and by a grant of the Spanish Ministry of Education and Science to JMF (PGC2018-094808-B-I00). Logistic and technical support was provided by ICTS-RBD. The authors acknowledge support of the publication fee by the CSIC Open Access Publication Support Initiative through its Unit of Information Resources for Research (URICI). The funders had no role in study design, data collection and analysis, decision to publish, or preparation of the manuscript.

Powered by Editorial Manager® and ProduXion Manager® from Aries Systems Corporation

**Unfunded studies**<br>Enter: *The author(s) received no specific funding for this work.*<br><br>**Funded studies**<br>Enter a statement with the following details:<br>• Initials of the authors who received each award<br>• Grant numbers awarded to each author<br>• The full name of each funder<br>• URL of each funder website<br>• Did the sponsors or funders play any role in the study design, data collection and analysis, decision to publish, or preparation of the manuscript?<br>• **NO** - Include this sentence at the end of your statement: *The funders had no role in study design, data collection and analysis, decision to publish, or preparation of the manuscript.*<br>• **YES** - Specify the role(s) played.<br><br>* typeset	
**Competing Interests**<br><br>Use the instructions below to enter a competing interest statement for this submission. On behalf of all authors, disclose any competing interests that could be perceived to bias this work—acknowledging all financial support and any other relevant financial or non-financial competing interests.<br><br>This statement is required for submission and **will appear in the published article** if the submission is accepted. Please make sure it is accurate and that any funding sources listed in your Funding Information later in the submission form are also declared in your Financial Disclosure statement.<br><br>View published research articles from *PLOS ONE* for specific examples.	The authors have declared that no competing interests exist.

Powered by Editorial Manager® and ProduXion Manager® from Aries Systems Corporation

**NO authors have competing interests**<br><br>Enter: *The authors have declared that no competing interests exist.*<br><br>**Authors with competing interests**<br><br>Enter competing interest details beginning with this statement:<br><br>*I have read the journal's policy and the authors of this manuscript have the following competing interests: [insert competing interests here]*<br><br>* typeset	
**Ethics Statement**<br><br>Enter an ethics statement for this submission. This statement is required if the study involved:<br><br>• Human participants<br>• Human specimens or tissue<br>• Vertebrate animals or cephalopods<br>• Vertebrate embryos or tissues<br>• Field research<br><br>Write "N/A" if the submission does not require an ethics statement.<br><br>General guidance is provided below. Consult the submission guidelines for detailed instructions. **Make sure that all information entered here is included in the Methods section of the manuscript.**	All permits necessary were granted by the National Park Service (ref. 2019/10) and the Junta de Andalucía (ref. 2019107300002261/IRM/MDCG/mes)

**Format for specific study types**<br><br>**Human Subject Research (involving human participants and/or tissue)**<br>• Give the name of the institutional review board or ethics committee that approved the study<br>• Include the approval number and/or a statement indicating approval of this research<br>• Indicate the form of consent obtained (written/oral) or the reason that consent was not obtained (e.g. the data were analyzed anonymously)<br><br>**Animal Research (involving vertebrate animals, embryos or tissues)**<br>• Provide the name of the Institutional Animal Care and Use Committee (IACUC) or other relevant ethics board that reviewed the study protocol, and indicate whether they approved this research or granted a formal waiver of ethical approval<br>• Include an approval number if one was obtained<br>• If the study involved *non-human primates*, add *additional details* about animal welfare and steps taken to ameliorate suffering<br>• If anesthesia, euthanasia, or any kind of animal sacrifice is part of the study, include briefly which substances and/or methods were applied<br><br>**Field Research**<br><br>Include the following details if this study involves the collection of plant, animal, or other materials from a natural setting:<br>• Field permit number<br>• Name of the institution or relevant body that granted permission	
**Data Availability**<br><br>Authors are required to make all data underlying the findings described fully available, without restriction, and from the time of publication. PLOS allows rare exceptions to address legal and ethical concerns. See the PLOS Data Policy and FAQ for detailed information.	Yes - all data are fully available without restriction

A Data Availability Statement describing where the data can be found is required at submission. Your answers to this question constitute the Data Availability Statement and **will be published in the article**, if accepted.<br><br>**Important:** Stating 'data available on request from the author' is not sufficient. If your data are only available upon request, select 'No' for the first question and explain your exceptional situation in the text box.<br><br>Do the authors confirm that all data underlying the findings described in their manuscript are fully available without restriction?	
**Describe where the data may be found in full sentences. If you are copying our sample text, replace any instances of XXX with the appropriate details.**<br><br>• If the data are **held or will be held in a public repository**, include URLs, accession numbers or DOIs. If this information will only be available after acceptance, indicate this by ticking the box below. For example: *All XXX files are available from the XXX database (accession number(s) XXX, XXX.*).<br>• If the data are all contained **within the manuscript and/or Supporting Information files**, enter the following: *All relevant data are within the manuscript and its Supporting Information files.*<br>• If neither of these applies but you are able to provide **details of access elsewhere**, with or without limitations, please do so. For example:<br><br>*Data cannot be shared publicly because of [XXX]. Data are available from the XXX Institutional Data Access / Ethics Committee (contact via XXX) for researchers who meet the criteria for access to confidential data.*<br><br>*The data underlying the results presented in the study are available from (include the name of the third party*	All files will be held in a public repository (FigShare) upon acceptance.<br><br>https://figshare.com/articles/dataset/PerchigEffectDwarfPalm_xlsx/19642446

and contact information or URL).<br>• This text is appropriate if the data are owned by a third party and authors do not have permission to share the data.<br><br>* typeset	
Additional data availability information:	Tick here if the URLs/accession numbers/DOIs will be available only after acceptance of the manuscript for publication so that we can ensure their inclusion before publication.

Powered by Editorial Manager® and ProduXion Manager® from Aries Systems Corporation

Revised manuscript Click here to access/download;Manuscript;Revised Manuscript.docx

Unmasking the perching effect of the pioneer Mediterranean palm *Chamaerops humilis* L.

Víctor González-García[1]* ¶, Pedro J. Garrote[2] ¶ and Jose M. Fedriani[3, 4] *¶

[1] Atlantic Botanical Gardens, Gijon, Asturias, Spain

[2] Centre for Applied Ecology "Prof. Baeta Neves" (CEABN/InBIO), Institute Superior of Agronomy, University of Lisbon, Tapada da Ajuda, Lisbon, Portugal

[3] Desertification Research Centre CIDE, CSIC UV GV, Moncada, Valencia, Spain

[4] Doñana Biological Station (EBD - CSIC), Seville, Spain

* Corresponding authors

victor.gon.gar@hotmail.com (VG), fedriani@csic.es (JMF)

¶ These authors contributed equally to this work.

Abstract

Although farmlands are the most extended terrestrial biomes, the abandonment of traditional agriculture in many parts of [illegible]rld has brought challenges for the restoration of such human-disturbed habitats. Seed arrival is a crucial necessary ecological process during the (re)colonization that can be enhanced by the use of the so-called "perch plants". Little is known about how spatial aspects of "perching" affect their effectiveness in disturbed habitats. To evaluate several spatial aspects of "perching" effect, including whether the seed arrival via frugivorous birds is associated to the spatial distribution of the perch plants, we used a spatially explicit approach in two disturbed plots within the Doñana National Park (SW Spain). Specifically, we chose as study system the pioneer Mediterranean dwarf palm *Chamaerops humilis* L., which is often used as perch by a variety of frugivorous bird species. A total of 289 *C. humilis* individuals were sampled in search of bird feces (N = 2998) and dispersed seeds (N = 529). Recorded seeds belonged to six different woody species from five different families. Nine bird species from six different families were recorded using *C. humilis* as perches. GLMs analyses indicated that taller *C. humilis* males with high richness of beneficiary woody species received more dispersed seeds. We detected a random spatial structure of bird feces and dispersed seeds in one study plot, while a nonrandom spatial structure was found in the other one, where isolated *C. humilis* received a higher number of bird feces and dispersed seeds than expected under spatial null models. The difference in spatial patterns between both study plots could relate, among other factors, to their different state of development in the ecological succession. Most of dispersed seeds were concentrated in a small number of *C. humilis* individuals (usually male and large ones) that acted as "hotspots" of seed arrival. The fact that frugivorous birds visited most often isolated *C. humilis* (rather than aggregated ones) questions the aggregated spatial structure of revegetation designs typically used in restoration projects. This study reveals novel spatial aspects of the "perching" effect which could be helpful in the restoration of human-disturbed habitats worldwide.

Introduction

Croplands and grazing lands have become the largest terrestrial biomes, occupying almost the 40% of Earth's land surface [1, 2]. However, in the Mediterranean Europe, areas destined to agriculture have been reduced by 34.2% in the last half century. In Spain, specifically, they have decreased by 54.8% [3, 4]. This abandonment of farmlands represents an opportunity to boost biodiversity through natural (and facilitated) revegetation and reforestation [5], and thus to restore habitats to the stages prior to human disturbances. Restoration projects are generally expensive given that they have been usually focused on planting dominant tree species [6]. Although many of such restoration projects have been successfully [7], they could be unaffordable when the affected area is too large, limiting the full recovery of vegetation structure [8]. Natural regeneration acts more widely and is less expensive than tree plantations [5], although its intensity varies by region and habitat. In arid and semiarid areas with low primary productivity such as the Mediterranean basin, natural restoration is clearly slower [7, 9] than in more humid and productive environments like tropics or temperate regions, being the water availability for plants an evident limitation [10]. Some other constrains in human-disturbed habitat restoration are inter- and intraspecific competition from herbaceous plants [11], the border effect or the past landscape pattern [12], and the limited propagule pressure and dispersal capacity of seeds [9, 13, 14].

Seed dispersal plays a key role in habitat dynamics and structure of plant communities [15]. Seed dispersal facilitates plant colonization of vacant areas and thus, to recover habitats that have returned to be suitable [16]. Birds and mammals are the main seed dispersers of most fleshy-fruited plants [17]. Many frugivorous birds often use tree and shrub branches as "perches", i.e. sites where they defecate and regurgitate large number of viable seeds [18, 19, 20]. Repeated visits to the same perches locally increase seed arrival and thus can trigger nucleation processes [20]. Human-disturbed habitats are more prone to these nucleation effects, since they are often vacant habitats where perches are scarce, which promotes a marked seed clumping [21, 22]. However, we are not aware of any study that has quantified the patterns of seed clumping under perches using a spatially explicit approach.

Interestingly, in some cases, the same shrub species acting as perches also act as nurse plants, i.e. facilitating the emergence, growth and survival of other plant species, [23] which are designated as “beneficiary species”, promoting the natural (re)colonization [24, 25]. *Chamaerops humilis* L., is a pioneer Mediterranean palm crucial in habitat recovery [26]. Our unpublished data indicate that these palms potentially act as perches for many (frugivorous) birds, which play important roles as seed dispersers and agents of the natural (re)colonization of human-disturbed areas by woody plants (e.g. [27]). The facilitative role of this keystone palm has been recently evaluated [28]. In particular, [4] the dwarf palm has been found positively associated with multiple woody plant species in disturbed vacant habitats. By contrast, the “perching” effect of *C. humilis* and its potential implications for the ecological restoration of human-disturbed landscapes remain unknown. In addition, very little is known about the pervasiveness of the natural “perching” effect and its spatial aspects and scales. On this matter, Spatial Point Pattern Analysis (SPPA) constitute a powerful tool to evaluate this sort of questions [29].

In this study we evaluate spatial patterns on the role of *C. humilis* as perch for frugivorous birds dispersing woody plant seeds in human-disturbed habitats. To achieve our goal, we selected two study plots within Doñana National Park (SW Spain) that differ in their history of human-driven perturbations and vegetation. We used a spatially explicit approach to answer the following questions: (*i*) How pervasive is the “perching” effect in our two studied human-disturbed habitats? (*ii*) Are there differences between the two study plots in the role of *C. humilis* as perch? And, if so, what are the factors underlying such differences? (*iii*) Do aggregated *C. humilis* receive more bird feces comprising dispersed seeds? (*iv*) Do particular *C. humilis* individuals act as "hot spots" of seed arrival, and if so, why? And (*v*) Do the species of dispersed seeds correspond to the woody beneficiary species found spatially associated to the *C. humilis*?

Material and methods

Study Species

Chamaerops humilis L. (Arecaceae), commonly known as "European fan palm" or "Mediterranean dwarf palm", is an evergreen shrub original from Western Mediterranean [30, 31]. This palm can grow up to 10m tall, although individuals are not usually taller than 2m [32]. Leaves are big, fan-shaped and displayed in numerous segments, exhibiting several long thorns on the petiole. It is dioecious, with fruits only growing on female plants. Flowering occurs in spring, from March to May. Spread takes place through seeds (sexually) or, less commonly, through division of adult trees (asexually) [30]. Seeds are mostly dispersed by medium-sized carnivores such as Eurasian badgers *Meles meles* and red foxes *Vulpes vulpes* [33] but also by ungulates (e.g. red deers *Cervus elaphus* or wild boars *Sus scrofa*) some of which occasionally disperse seeds by regurgitation [34, 35].

The facilitative role of *C. humilis* and its capacity of recolonizing disturbed habitats have made it widely used in ecological restorations [36], although its role as nurse plant has only recently been demonstrated [4]. *Chamaerops humilis* protect seeds and seedlings of woody species from high temperatures and drought, as well as from herbivory thanks to the spikes present in its leaves [4, 37]. Frequent visits of some seed dispersers such as red foxes could lessen seed and seedling predation of beneficiary plants by several species of rodents (e.g. *Apodemus sylvaticus*) and lagomorphs (e.g. *Oryctolagus cuniculus*), which use nurse plants as shelters from their predators [35].

In its typical habitat, the Mediterranean scrubland, *C. humilis* is often spatially associated with *Asparagus* L. spp., *Daphne gnidium* L., *Olea europaea* L. var. *sylvestris* (Mill.) Hegi, *Pistacia lentiscus* L. or *Pyrus bourgaeana* Mecde, and other woody species. These spatial associations are thought to be due to a combination of "nursing" and "perching" effects [4, 30], although the "perching" effect has not been tested yet.

Study area and plots

The study was carried out within the Doñana National Park (SW Spain, on the west of the Guadalquivir River estuary). All permits necessary were granted by the National Park Service (ref. 2019/10) and the Junta de Andalucía (ref. 2019107300002261/IRM/MDCG/mes). The National Park presents a Mediterranean sub-humid climate, with most of rainfalls taking place from November to April, showing an average annual rainfall of 500-600mm [33], with hot dry summers (June-September) and template humid winters (October-January).

We selected two study plots 10 km apart called Matasgordas (area sampled: 13.9 ha) and Doñana Biological Reserve (area sampled: 21.4 ha) (Fig 1). Study areas are different in terms of vegetation and human-use history [4, 38]. Matasgordas has been historically and intensively affected by anthropic management of the vegetation and livestock pressure [39]. The eastern portion of this study plot was drastically modified in 1970 when most of woody plants (both shrubs and trees) were removed in order to create a "dehesa", becoming an open area for cattle grazing with scattered trees, mostly *Quercus suber* L. and *O. europaea* var. *sylvestris* [4, 16]. In 1996, the land became owned by Government and happened to be under the protection of the National Park boundaries, putting an end to the cattle grazing. Once livestock was removed from the area, native fleshy-fruited plants such as *C. humilis*, *D. gnidum*, *P. lentiscus* and *P. bourgaeana* started to recolonize their habitat via seed dispersal by birds and mammals [4, 16]. Due to such past human activity, Matasgordas is now formed, mainly, by two habitats: (*i*) Scrubland dominated by *P. lentiscus* shrubs with some *Q. suber* and *O. europaea* var. *sylvestris trees* [4, 33] and (*ii*) an old-field, where our study plot was set, which woody vegetation is mainly composed by animal-dispersed native plants such as *C. humilis*, *P. lentiscus*, *D. gnidium* and *P. bourgaeana*, *Asparagus aphyllus* L., *Halimium halimifolium* (L.) Willk and *Cistus salviifolius* L. and scattered *Q. suber* and *O. europaea* var. *sylvestris* trees [4]. The distribution of *C. humilis* in this plot is very aggregated, and best described by a double-clustered component process with a random component process (with 22% of isolated *C. humilis* individuals; see Supporting Information [S1 Cluster Analysis]).

Figure 1. **The study plots.** (A) Darkened location of the Doñana National Park within (NW) Spain. (B) Doñana Natural Park with the two study plots highlighted in black, being Matasgordas the northern one and Reserva the southern one. (C) Matasgordas and (D) Reserva study plots located within the Doñana National Park. Every occurring *C. humilis* was georeferenced (black solid points).

Doñana Biological Reserve is located in the centre of the National Park. It is laid on a coastal plain distinguished for its flatness and being separated from the sea by lagoons and mobile dune systems. However, it is this closeness to the sea which allows a humid and mild climate [40] and enables the existence of three different types of habitats: marshland, dunes and scrubland; being the ecotone between the scrubland and the marshes (locally known as "La Vera") where we set our study plot. This area has been historically managed by human for agriculture, hunting, cattle grazing and tree felling, especially *O. europaea* var. *sylvestris* and *Q. suber* [41]. Reserva was protected earlier than Matasgordas, in 1964 and it has been recovering ever since, being recolonised by animal-dispersed plants such as *A. aphyllus*, *C. humilis*, *Phillyrea angustifolia* L. or *R. ulmifolius* [4]. This scrubland is dominated by *H. halimifolium* and *Stauracanthus genistoides* (Brot.) Samp. with scattered trees of *Q. suber, O. europaea* var. *sylvestris* and *Pinus pinea* [33]. The study plot at Reserva presents higher density of shrubs than the one at Matasgordas. Such a difference in shrub cover led to a greater abundance of fleshy fruits in Reserva, which likely makes this site more attractive to frugivorous birds [4]. The distribution of *C. humilis* in this plot is very aggregated, and best described by a double-clustered component process with a random component process (with 9% of isolated *C. humilis* individuals; see Supporting Information [S1 Cluster Analysis]).

Data Collection

Every target *C. humilis* (109 and 180 in Matasgordas and Reserva, respectively) was individually georeferenced with a submetric GPS Leica 1200. The density of *C. humilis* plants in Matasgordas' plot (7.78 individuals/ha) was pretty similar than in Reserva' plot (8.41 individuals/ha). Each study plot was visited eight and five times along the seed dispersal seasons (i.e. from September to November) of 2019 and 2020, respectively. To control for potential temporal variations on bird abundance and seed dispersal along the dispersal seasons, both study plots were sampled during the same sampling days. Rainy days were avoided because of the low bird activity and the feces wash out on *C. humilis*.

During the sampling, which lasted about 3h per day in Matasgordas and about 2h in Reserva, any observed bird perching on focal *C. humilis* individuals was recorded. Overall, nine bird species were recorded perching: *Cisticola juncidis*, *Lanius senator*, *Phoenicurus ochruros, Phylloscopus collybita, Pica pica, Saxicola rubicola*, *Sturnus unicolor*, *Sylvia atricapilla* and *Sylvia melanocephala* (Fig 2, Table 1).

Figure 2. Birds using *Chamaerops humilis* as perches in Doñana National Park. (A) Eurasian magpie (*Pica pica*). (B) Common stonechat (*Saxicola rubicola*). (Photo credit: Pedro J. Garrote)

Table 1. **Summary of collected data**.

	MATASGORDAS		RESERVA	
	2019	2020	2019	2020
Chamaerops humilis (individuals)	109		180	
Number of feces	705	206	1560	527
Number of seeds	28	16	340	145
C. humilis with feces (%)	42.2	37.6	43.9	27.8
Feces with seeds (%)	3.3	17.8	7.5	7.8
C. humilis with seeds (%)	8.3	11.9	13.9	7.8
Asparagus aphyllus (seeds)	1	7	16	3
Daphne gnidium (seeds)	7	5	1	2
Olea europaea var. *sylvestris* (seeds)	0	0	26	8
Phillyrea angustifolia (seeds)	0	0	4	0
Pistacia lentiscus (seeds)	20	4	2	0

Rubus ulmifolius (seeds)	0	0	291	132
**Perching birds**				
Cisticola juncidis	1	1	0	2
Lanius senator	0	0	2	0
Phoenicurus ochruros	0	0	3	1
Phylloscopus collybita	0	0	1	0
Pica pica	0	0	2	0
Saxicola rubicola	2	1	7	4
Sturnus unicolor	0	0	2	0
Sylvia atricapilla	1	1	1	2
Sylvia melanocephala	3	3	14	4
**Beneficiary species**				
Asparagus aphyllus	35		96	
Cistus sakviifolius	16		0	
Daphne gnidium	1		0	
Olea europaea var. *sylvestris*	6		20	
Pistacia lentiscus	3		0	
Pyrus bourgaeana	1		0	
Quercus suber	1		5	
Rubus ulmifolius	3		7	
Stauracanthus genistoides	31		17	

Number of samples (feces, seeds) collected in each study plot, the number of seeds found for each plant species, as well as the number and species of bird recorded perching on *C. humilis*.

Every target *C. humilis* was checked for bird feces and regurgitations by carefully checking the palm foliage surface. We also evaluated whether seeds arrive at open spaces without palms. To this aim, we checked for the presence of bird feces and regurgitations in an open area associated to each focal *C. humilis*. These areas were of similar surface and mostly located in bare soils with scarce vegetation (mainly grasses) and 3-4m from the associated *C. humilis*. Every found excrement and regurgitated seed was collected and stored inside of an individual Eppendorf tube. In the lab, feces were dried to make their handling easier. Once dried, every sample was checked for seeds by crumbling the excrement and looking for hard matter, either of vegetable (seeds, bark) or animal (elytra and other arthropod parts) origin. Found seeds were identified at the species level using images of a reference collection (Fig 3), considering its shape, size and the surface design. All collected seeds (N = 529) were positively identified.

Figure 3. Examples of collected feces and identified dispersed seeds. (A) Bird excrement on a leaf of *Chamaerops humilis* in Matasgordas (B) *Asparagus aphyllus.* (C) *Daphne gnidium.* (D) *Olea europaea* var. *sylvestris*. (E) *Phillyrea angustifolia*. (F) *Pistacia lentiscus*. (G) *Rubus ulmifolius*. (Photo credit: Pedro J. Garrote).

To assess the potential selection by birds of particular traits of *C. humilis* individuals, we measured the following palm variables: gender (male, female, undetermined), size (height, area and volume), presence and richness of beneficiary woody species within them and beneficiary height (<25cm, 25-100cm, >100cm). Beneficiary species were woody plants that grow under dwarf palms and emerge from the top of their surface. These species often benefit from *C. humilis* due to microclimatic improvement and protection against herbivory [42].

Spatial analyses

Aggregation of *C. humilis* used as perches within the overall pattern *of C. humilis*

Every *C. humilis* was characterized with its spatial location and a mark (presence/absence of bird feces in this case). To find out if *C. humilis* used as perches by birds (i.e. receiving bird feces) show a spatial pattern (aggregated, isolated) or they instead are randomly distributed we used qualitatively marked point patterns techniques and the random labelling null models to evaluate lack of any spatial structure. The random labelling null model consists in giving the mark “with feces” or “without feces” over all *C. humilis* (having or not feces) randomly, conducting a total of 199 simulations [38].

In order to quantify the spatial pattern of *C. humilis* used as perches within the overall pattern of *C. humilis* we used mark connection functions [43, 44, 45] as summary statistics [38]. Whether *C. humilis* contained feces they were assigned with a mark of value 1 and a mark of

value 0 if they had no feces. The mark connection function $p_{ij}(r)$ gives the probability that two *C. humilis*, separated by a distance *r*, the first is type *i* (i.e. with feces) and the second is type *j* (without feces).

Finally, to test if *C. humilis* with feces are located in areas of overall high density of neighbouring *C. humilis* (with feces or without them) we used the test statistic $g_{i,i+j}(r) - g_{j,i+j}(r)$ [44], which consist in comparing the density of *C. humilis* ($i + j$) around *C. humilis* with feces (type *i*) with the density of *C. humilis* ($i + j$) around *C. humilis* without feces (type *j*). Under random labelling, the expected value for this statistic is zero: in case that feces tend to be delivered in palm aggregations we expected $g_{i,i+j}(r) > g_{j,i+j}(r)$ and, if not, if there is an isolation of feces, the we expected $g_{i,i+j}(r) < g_{j,i+j}(r)$.

To test the fit of data with the point process and departures from the null model, we conducted 199 simulations (for every analysis) of the fitted point processes and estimated envelopes with an approximate error rate of $\alpha = 0.05$ [46] which are the fifth lowest and highest values of the summary statistics of the simulated point process. Observed values above the top or below the bottom simulation envelopes indicate higher or lower than expected spatial aggregation, respectively. Observed values within the simulation envelopes indicate a level of spatial aggregation compatible with the stochasticity of the point process model. For all point pattern analyses, we used the grid-based software *Programita* [47].

Aggregation of *C. humilis* receiving dispersed seeds within the overall pattern of *C. humilis*

To assess if *C. humilis* containing (feces with) dispersed seeds were randomly distributed within the pattern of all *C. humilis* (with and without seeds) or they follow a particular spatial pattern (aggregation, dispersion), we also used qualitatively marked point pattern techniques and the random labelling null model to represent lack of any spatial structure. The same process and analysis were conducted as in the previous case but, in this occasion, the used mark was the

presence (1) or absence (0) of seed in each individual *C. humilis*. In parallel, these same analyses were carried out but excluding the *Rubus'* seeds since their great number could distort the spatial pattern for the presence of seeds on *C. humilis* (see Supporting Information, S2 Spatial analysis excluding *Rubus'* seeds). Since results were rather consistent whether we considered or not *Rubus*'s seeds, here we provide the results when all seed species are included.

Association of the number of dispersed seeds on *C. humilis*

To find out whether the number of dispersed seeds within bird feces deposited on *C. humilis* has a spatial association among different *C. humilis* we used quantitative marked point patterns techniques and the random labelling null model to represent lack of any spatial structure.

To quantify potential spatial associations in the number of seeds on different *C. humilis* we used the univariate mark correlation function as summary statistic [38, 43, 47, 48], ergo we wanted to find out if closer *C. humilis* had a higher number of seeds than isolated ones. In addition to its location, each *C. humilis* was characterized by a quantitative mark: (i.e. the number of dispersed seeds (of any species).

Mark correlation functions are based on pairs of study units (in this case *C. humilis* individuals) and the idea of estimating the mean value of a test function $t(m_i, m_j)$ of the two marks m_i and m_j, taken over all the ordered pairs *i-j* of *C. humilis* which have interpoint distances of $r \pm h$, being h the parameter bandwidth, which must be wide enough to produce a sufficient number of pairs in each distance class r but small enough to reveal significant biological detail [43, 49]. From all possible test functions $t(m_\mathrm{i}, m_\mathrm{j})$ [43], we selected the following three:

(*i*) The r-mark correlation function $\mathrm{k_{m1.}}\,(r)$ which is based on the test function: $t(m_i, m_j) = m_i$. $\mathrm{k_{m1.}}\,(r) > 1$ indicates that the number of seeds on *C. humilis* that have neighbouring *C. humilis* at distance r is larger than the average of seeds, showing a positive effect of *C. humilis* aggregation on seed number. By contrast, if $\mathrm{k_{m1.}}\,(r) < 1$, it would indicate a negative effect of aggregation and

a positive effect of isolation of *C. humilis* on seed number [49]. Finally, if $k_{m1.}$ $(r) \sim 1$, indicates that the number of seeds is not affected by the distance to *C. humilis* individuals.

(*ii*) Another mark correlation function was calculated to characterize the spatial covariance in number of seeds of two *C. humilis* separated by distance *r*. This test function is known as Schlather's: $t(r, m_i, m_j) = [m_i - \mu(r)][(m_j - \mu(r)]$ which results in a Moran's I like summary statistic $I_{mm}(r)$, a spatial variant of the Pearson correlation coefficient between the variables m_i and m_j defined by the ordered *i-j* pairs of *C. humilis* separated by distance $r \pm h$.

(*iii*) Lastly, we used a function that relates the number of seeds on a *C. humilis* directly to the density of neighbouring *C. humilis*, called "density correlation function" [49]: $C_{m,K}(r)$. This function estimates the Pearson correlation coefficient between the number of seeds m_i and the number of neighbours within distance *r*, based on the following equation: $t(r, m_i, K_i) = [m_i = \mu][(\lambda K_i(r) - \lambda K_i(r)]$. Where m_i is the number of seeds of the focal *C. humilis* *i*, λ is the overall density of *C. humilis* in the study area, μ is the mean number of seeds of the population, $\lambda K_i(r)$ is the number of neighbours around the focal *C. humilis i* within distance *r* for all *C. humilis*.

To test whether the data show spatial correlation, the observed values of the three functions mentioned above were contrasted to the data obtained from simulations of a null model without spatial correlation in the marks. The null model consisted in shuffling randomly the marks (number of seeds) over all *C. humilis*, conducting a total of 199 simulations. These simulations were also useful to estimate the simulation envelopes for the three functions, being the fifth highest and lowest values of the summary statistics among all simulations of the null mode [38, 49].

Effect of palm traits

To analyse how our response variables (presence of bird feces, presence of dispersed seeds and number of dispersed seeds) were influenced by the size (height, area and volume) and gender (male, female) of *C. humilis* individuals, as well as by the presence (and height: <25cm, 25-

100cm, >100cm) of beneficiary species, three sets of generalized linear models (GLMs) were fitted per study plot.

Model 1 assessed the effect of palm traits on the likelihood of presence of bird feces, Model 2 evaluated the probability of finding dispersed seeds depending on such characteristics and Model 3 appraised the number of seeds based on those characteristics. Both Model 1 and Model 2 have a binary response variable (presence = 1, absence = 0), and thus were fitted with a binomial distribution and a logit link. Finally, Model 3, was fitted with a Poisson distribution with logit link.

A set of competing models were fitted and the best candidate model was selected based on Akaike's Information Criterion (AIC), allowing to compare different hypothesis and know their supportive level on the data by weighting them (AICc; [50]). All analyses were performed in R 4.0.3 statistical software [51]. Additionally, these same models were carried out but using the interaction between *C. humilis* Height (m) and Area (m^2) instead of the variable Volume (m3). Results were rather consistent with those from the previous analysis (see Supporting Information, S3 Effect of palm traits).

Results

Overall patterns

We studied total of 109 *C. humilis* in Matasgordas' plot, from which were collected 705 bird feces and 28 seeds in 2019 and 206 bird feces and 16 seeds in 2020 (Fig 4). In Reserva's plot, we monitored 180 *C. humilis*, having been collected 1560 feces and 340 seeds in 2019, and 527 feces and 145 seeds in 2020. Nevertheless, not all dispersed seeds were contained within the feces since a small fraction was regurgitated by the birds. Six shrub species were identified among the collected seeds: *A. aphyllus*, *D. gnidium*, *O. europaea* var. *sylvestris*, *P. angustifolia*, *P. lentiscus* and *R. ulmifolius*. Finally, a large fraction of *C. humilis* acted as perch for birds and

contained their feces: 42.2% (2019) and 37.6% (2020) in Matasgordas and 43.9% (2019) and 27.8% (2020) in Reserva (Table 1).

Figure 4. **Distribution of *C. humilis* in Matasgordas (upper) and Reserva (lower) plots.** (A) *C. humilis* in Matasgordas with (black solid points) and without (white circles) bird feces or regurgitations. (B) *C. humilis* in Matasgordas with (black solid points) and without (white circles) dispersed seeds. (C) *C. humilis* in Reserva with (black solid points) and without (white circles) bird feces. (D) *C. humilis* in Reserva with (black solid points) and without (white circles) dispersed seeds.

Besides, some *C. humilis* had beneficiary woody plants growing within them. In Reserva and Matasgordas 104 out of 180 individuals (57.78%) and 71 out to 109 (65.14%) had beneficiary plants, respectively. A total of nine different beneficiary species were found, all of them present in Matasgordas but just five were recorded in Reserva (Table 1).

Spatial patterns

Aggregation of *C. humilis* used as perches within the overall pattern of *C. humilis*

Analysis of how *C. humilis* used as perches by birds (i.e. receiving bird feces) were distributed within the overall pattern of *C. humilis* showed the following results. In Matasgordas, the probability that two *C. humilis*, separated by a distance *r*, both used as perches (statistic $p_{11}(r)$), was contained within the expectation of random labelling (Fig 5A). The probability that two separated *C. humilis*, with just one comprising feces (statistic p_{12}(r)), was included within the simulation envelops generated by random labelling (Fig 5C). Finally, the observed density of neighbouring *C. humilis* (regardless of having feces or not; statistic $g_{i,i+j}(r) - g_{j,i+j}(r)$) around *C. humilis* with feces was within the simulation envelops generated by a random labelling mode (Fig

5E). Thus, in Matasgordas, the observed probabilities described by the three statistics were within the envelops generated by random labelling models, which indicates they are compatible with the stochasticity of the process model.

On the contrary, in Reserva, the probability that two *C. humilis* separated for distances 1-6m, both having feces ($p_{11}(r)$ statistic), was lower than the expectation under random labelling. The bivariate $p_{12}(r)$ showed that *C. humilis* with and without feces were further away than expected under random labelling up to scales of 6m (Fig 5D). Finally, the density of neighbouring *Chamaerops humilis* (having or not feces) around *C. humilis* with feces was significantly lower than expected by random labelling for distances up to scales of 7m (Fig 5F). Overall, these results indicate a tendency by frugivorous birds in Reserva towards using and defecating in isolated *C. humilis* individuals.

Figure 5. **Analysis of *C. humilis* with feces in the two study plots (Matasgordas and Reserva) areas using mark connection functions as summary statistics.** (A, B) The mark connection function $p_{11}(r)$ gives the conditional probability that, from two *C. humilis* that are separated by distance *r*, both are type 1 (i.e., with feces). (C, D) The mark connection function $p_{12}(r)$ gives the conditional probability that, from two *C. humilis* that are separated by distance *r*, the first is type 1 (i.e., with feces) and the second is type 2 (i.e., without feces). (E, F) The test statistic $g_{1,1+2}(r)$ - $g_{2,1+2}(r)$ compares the density of *C. humilis* (i.e., 1+2) around *C. humilis* with feces (i.e., type 1) with the density of *C. humilis* (i.e., 1+ 2) around *C. humilis* without feces (i.e., type 2). The expected mark connection function statistics (gray line) and the corresponding simulation envelopes (black lines), being the fifth lowest and highest values of the functions created by 199 simulations under random labelling, are also shown.

Distribution of *C. humilis* receiving dispersed seeds within the overall pattern of *C. humilis*

In Matasgordas, the probability $p_{11}(r)$, where two *C. humilis* separated by distance *r*, both having seeds, was contained within the envelopes of random labelling for all spatial scales (Fig

6A). The bivariate $p_{12}(r)$, the probability for two neighbouring *C. humilis*, one with seeds and other without them, was also contained between random labelling's envelopes (Fig 6C). Finally, the test statistic $g_{1,1+2}(r)$ - $g_{2,1+2}(r)$ shows that the density of neighbouring *C. humilis* around *C. humilis* receiving seeds did not differ from the mean *C. humilis* density. (Fig 6E).

Results in Reserva substantially differed from results from Matasgordas. The probability of two *C. humilis* separated by distance *r*, having both seeds ($p_{11}(r)$ statistic), was similar to expected of random labelling for all spatial scales (Fig 6B). However, the probability for two neighbouring *C. humilis*, just one of them with seeds ($p_2(r)$ statistic), was significantly lower ($P = 0.01$) that the obtained by using random labelling for distances up to 6m, between 12 and 29m and higher than 41m (Fig 6D). Neighbourhood density of *C. humilis* around *C. humilis* receiving seeds was significantly lower ($P = 0.005$) than the mean *C. humilis* density at scales of 1 to 6m (Fig 6F).

In short, the analysis of how *C. humilis* with seeds were distributed within the overall pattern of *C. humilis* showed that *C. humilis* with seeds were randomly distributed in Matagordas (Fig 6A, C, E) while, in Reserva, *C. humilis* with seeds were spatially isolated at small spatial scale (Fig 6D, F).

Figure 6. **Analysis of feces with seeds in the two study plots (Matasgordas and Reserva) using mark connection functions as summary statistics**. (A, B) The mark connection function $p_{11}(r)$ gives the conditional probability that, from two *C. humilis* that are separated by distance *r*, both are type 1 (i.e., with seeds). (C, D) The mark connection function $p_{12}(r)$ gives the conditional probability that, from two *C. humilis* that are separated by distance *r*, the first is type 1 (i.e., with seeds) and the second is type 2 (i.e., without seeds). (E, F) The test statistic $g_{1,1+2}(r)$ - $g_{2,1+2}(r)$ compares the density of *C. humilis* (i.e., 1+2) around *C. humilis* with seeds (i.e., type 1) with the density of *C. humilis* (i.e., 1+ 2) around *C. humilis* without seeds (i.e., type 2). The expected mark connection function statistics (gray line) and the

corresponding simulation envelopes (black lines), being the fifth lowest and highest values of the functions created by 199 simulations under random labelling, are also shown.

Distribution of the number of dispersed seeds across *C. humilis*

The observed r- mark correlation function for the number of seeds was included within the simulation envelop for all scales, indicating no relationship between distance among individual *C. humilis* and the number of dispersed seeds neither in Matasgordas (*P* = 0.95, Fig 7A) nor in Reserva (*P* = 0.31, Fig 7B). Similarly, the Schlather's correlation function did not show a correlation between number of seeds and distance among individuals at any spatial scales, neither in Matasgordas (*P* = 0.22, Fig 7C) nor in Reserva (*P* = 0.22, Fig 7D). Finally, the density correlation function did not indicate correlation between the number of seeds and the density of *C. humilis* at any scale neither in Matasgordas (*P* = 0.35, Fig 7E) nor in Reserva (*P* = 0.35, Fig 7F).

Figure 7. **Mark correlation functions to evaluate a potential spatial structure in the number of dispersed seeds found on *C. humilis* in Matasgordas and Reserva study plots.** (A, B) The r- mark correlation function describes the mean number of seeds (m_i) on a *C. humilis* at distance r of another *C. humilis*. (C, D) Schlather's correlation function quantifies the correlation between the number of seeds on two different *C. humilis* separated by distance r. (E, F) Density correlation function assesses the correlation between the number of seeds and the number of neighbours located at a distance r. The expected mark connection function statistics (gray line) and the corresponding simulation envelopes (black lines), being the fifth lowest and highest values of the functions created by 199 simulations under random labelling, are also shown.

Effect of palm traits

We found that *C. humilis* in Matasgordas were on average (8.80 m^2) smaller than in Reserva (15.26 m^2). Also, the richness of beneficiary species beneath *C. humilis* was slightly lower in Matasgordas than in Reserva, with 0.89 and 1.07 beneficiary species per individual, respectively.

For Matasgordas, the best model (AIC = 133.97) showed that presence of bird feces on *C. humilis* were positively related to their height and area, but negatively to volume, although none of these variables were found significant (Table 2. Model 1). The best model for Reserva (AIC = 196.63) indicated that presence of bird feces on *C. humilis* was higher in male than females and positively influenced by palm area, but negatively to the richness of beneficiary species contained within them. Nevertheless, only the palm area was found significant ($P < 0.001$).

Table 2. GLM carried out to analyze the palm traits Matasgordas' plot.

Explanatory variable	Competing models		β	SE	AICc	ΔAIC	Weighted AIC
Model 1							
Presence of Bird Feces	Height (m) + Area (m^2) + Volume (m^3)				133.97	0	0.62
		Intercept*	-4.5863	2.2444			
		Height (m)	5.9296	3.5128			
		Area (m^2)	0.4108	0.2187			
		Volume (m^3)	-0.4497	0.2822			
	Gender + Height (m) + Area (m^2) + Volume (m^3)				135.67	1.7	0.27
	Gender + Height (m) + Area (m^2) + Volume (m^3) + Richness				137.54	3.57	0.10
	Gender + Height (m) + Area (m^2) + Volume (m^3) + Richness + Beneficiary height (cm)				143.11	9.14	0.01
Deviance = 90.343							
Model 2							
Presence of Dispersed Seeds	Gender + Height (m)				96.343	0	0.05
		Intercept*	-4.9694	1.4405			
		Gender (Male)	0.9151	0.557			
		Height (m)*	4.2936	1.8368			
	Gender + Height (m) + Volume (m^3)				98.21	1.87	0.02
	Gender + Height (m) + Area (m^2) + Volume (m^3)				99.03	2.69	0.01
	Gender + Height (m) + Area (m^2) + Volume (m^3) + Richness				100.73	4.39	< 0.01
	Gender + Height (m) + Area (m^2) + Volume (m^3) + Richness + Beneficiary height (cm)				107.56	11.2	< 0.01
Deviance = 126.52							
Model 3							
Number of Dispersed Seeds	Gender + Height (m) + Volume (m^3)				191.41	0	0.11
		Intercept*	-5.9884	1.15268			
		Gender(Male)*	0.82204	0.35402			
		Height (m)*	7.7374	2.03287			
		Volume (m^3)*	-0.1341	0.06466			
	Gender + Height (m) + Area (m^2) + Volume (m^3)				192.77	1.36	0.06
	Gender + Height (m) + Area (m^2) + Volume (m^3) + Richness				194.47	3.06	0.02

	Gender + Height (m) + Area (m^2) + Volume (m^3) + Richness + Beneficiary height (cm)				196.18	4.77	0.01
Deviance = 133.75							

Summary of fitted parameters and models employed to analyse the presence of bird feces (model 1), the presence of dispersed seeds (model 2) and the number of dispersed seeds (model 3) on *C. humilis* in Matasgordas study plot. Competitive models are ranked from the lowest AICc value (best model) to the highest one, being significant variables indicated with asterisks (*).

Presence of dispersed seeds in Matasgordas depended on *C. humilis* gender and height (Table 2. Model 2), according to the best model obtained (AIC = 96.343). Nonetheless, only the height was significant ($P < 0.05$) with larger *C. humilis* individuals having a higher number of dispersed seeds. In Reserva, the best model obtained (AIC = 137.98) showed that height and richness of beneficiary species had a positive effect on the number of dispersed seeds, although just the last one was significant in this case ($P < 0.01$) (Table 3. Model 2).

Table 3. GLM carried out to analyze the palm traits Reserva's plot.

Explanatory variable	Competing models		β	SE	AICc	ΔAIC	Weighted AIC
Model 1							
Presence of Bird Feces	Gender + Area (m^2) + Richness				196.63	0	0.54
		Intercept*	-0.7758	0.30364			
		Gender (Male)	0.55831	0.35223			
		Area (m^2)*	0.0891	0.02394			
		Richness	-0.4846	0.26617			
	Gender + Area (m^2) + Volume (m^3) + Richness				197.88	1.25	0.29
	Gender + Height (m) + Area (m^2) + Volume (m^3) + Richness				199.49	2.86	0.13
	Gender + Height (m) + Area (m^2) + Volume (m^3) + Richness + Beneficiary height (cm)				201.76	5.13	0.04
Deviance = 188.63							
Model 2							
Presence of Dispersed Seeds	Height (m) + Richness				137.98	0	0.62
		Intercept*	-3.3834	0.5486			
		Height (m)	0.4797	0.2728			
		Richness*	1.9713	0.7313			
	Height (m) + Volume (m^3) + Richness				139.86	1.88	0.24
	Height (m) + Area (m^2) + Volume (m^3) + Richness				141.64	3.66	0.10
	Gender + Height (m) + Area (m^2) + Volume (m^3) + Richness				143.5	5.52	0.04
	Gender + Height (m) + Area (m^2) + Volume (m^3) + Richness + Beneficiary height (cm)				147.44	9.46	< 0.01
Deviance = 131.98							
Model 3							
Number of Dispersed Seeds	Gender + Height (m) + Area (m^2) + Volume (m^3) + Richness + Beneficiary height (cm)				1273.09	0	1
		Intercept*	-2.8228	1.07845			
		Gender (Male)*	1.80173	0.12685			
		Height (m)*	-1.9455	0.45779			
		Area (m^2)*	-0.1135	0.01363			
		Volume (m^3)*	0.13377	0.01437			
		Richness*	0.95673	0.0926			

	Beneficiary height (25-100cm)	0.7794	1.03393
	Beneficiary height (>100cm)*	2.59102	1.0217
Deviance = 1155.7			

Summary of fitted parameters and models employed to analyse the presence of bird feces (model 1), the presence of dispersed seeds (model 2) and the number of dispersed seeds (model 3) on *C. humilis* in Reserva study plot. Competitive models are ranked from the lowest AICc value (best model) to the highest one, being significant variables indicated with asterisks (*).

According to the best model in Matasgordas (AIC = 191.41), male *C. humilis* had a higher number of dispersed seeds than females ($P < 0.05$) and height had a positive and significant ($P < 0.001$) influence on the number of seeds found on *C. humilis*. Palm volume had a negative effect on the number of seeds ($P < 0.05$) (Table 2. Model 3). Finally, in Reserva, just one model was fitted (AIC = 1273.09). This model predicted: (*i*) a negative relationship between height of *C. humilis* and the number of dispersed seeds ($P < 0.0001$); (*ii*) a higher number of dispersed seeds in male *C. humilis* than in females ($P < 0.0001$); (*iii*) a positive relationship between *C. humilis* size and the number of dispersed seeds ($P < 0.0001$ for both area and volume); (*iv*) a positive relationship between the richness of beneficiary species in *C. humilis* and the number of dispersed seeds (P < 0.0001); and (*v*) a higher number of dispersed seeds in *C. humilis* holding tall (> 100 cm in height) beneficiary woody individuals as compared to both small (< 25 cm) and intermediate (25-100 cm) beneficiary individuals ($P < 0.05$; Table 3, Model 3).

Discussion

The role of pioneer plants acting as perches for seed dispersers is crucial for the restoration of many human-disturbed habitats by increasing seed arrival and eventually seedling establishment [52, 53, 54]. Little is known concerning how the spatial distribution of perches influences the spatial patterns of seed dispersal by frugivorous birds, and the subsequent patterns of establishment of woody species [55]. Using a spatially explicit approach, we detected a random spatial structure in both the number of visits (bird feces) and the dispersed seeds found on *C. humilis* in one study plot (Matasgordas) while an aggregated pattern in the other one (Reserva).

Frugivorous birds tended to disperse seeds into a small number of *C. humilis* individuals (usually male and large-sized ones) that acted as “hotspots” of seed arrival. Our study also revealed that the species of dispersed seeds partially matched the woody beneficiary species growing inside *C. humilis*, reinforcing the idea that this palm species plays a key role in the restoration of disturbed habitats.

Widespread perching effect by *C. humilis*

Considering that our sampling period did not take place throughout the year and that an unknown fraction of the bird feces presents in target *C. humilis* individuals surely went unnoticed, the data obtained could be considered as a hint of how widespread the "perching” effect in both study plots. Therefore, the high presence of (frugivorous) bird feces and dispersed seeds in both study plots is remarkable.

Besides, it is apparent that some seeds can emerge seedling beneath *C. humilis*, since all seeds collected belonged to beneficiary woody species found within *C. humilis*. Nevertheless, we did not find seeds of the beneficiary species *C. salviifolius*, *P. bourgaeana*, *Q. suber* and *S. genistoides*, which are usually associated to *C. humilis*. Most of these plant species are seldom dispersed by birds. Other animals are more likely to disperse them, such as insects for *C. salviifolius* or *S. genistoides* seeds [56, 57, 58] or mammals, such as ungulates (red deer, wild boars, domestic cattle) and carnivores (Eurasias badger, red fox) for *P. bourgaeana* seeds [59]. Therefore, the presence of these woody species associated to *C. humilis* reveals that it does not attract just bird-dispersed plants but also mammal-dispersed plants, accentuating its role in habitat restoration.

Spatial patterns of the perching effect

Our study has revealed different spatial patterns in both study plots at the three target organizational levels studied: (*i*) *C. humilis* receiving bird feces, (*ii*) *C. humilis* receiving (feces with) dispersed seeds, and (*iii*) number of dispersed seeds arriving to *C. humilis*.

No spatial pattern was found in Matasgordas' plot, where the distribution of *C. humilis* did not seem to play a decisive role in attracting frugivorous birds. By contrast, in Reserva's plot, where isolated palms were more frequent than in Matasgordas, there was strong evidence that birds preferred isolated *C. humilis*, at least for those separated from conspecifics by a distance up to 7 m. Aggregated perches could be found attractive for seed dispersers and, therefore, more visited by them [60], since they would act as shelters against predators [59]. This premise has been used for designing ecological restoration and conservation actions in mixed ecosystems, where trees or other woody plants, acting as perches and nurses, are planted following aggregated spatial patterns [62, 63, 64, 65]. However, by concentrating frugivorous activity to aggregated planted trees, seed dispersal could be limited [16, 55]. Therefore, our results supporting the role of isolated perches diverge from the most widespread tendency in ecological restoration of aggregating trees and shrubs during revegetation efforts [65].

Spatial patterns of perching effect differed between study plots. On the one hand, these differences could be attributed to the changing distance that frugivorous birds must fly between the main seed source area (Mediterranean scrubland) and our study plots where target *C. humilis* individuals are located. This distance is longer in Matasgordas (up to 350m) than in Reserva (150-200m). Besides, it seems reasonable to expect that birds select *C. humilis* located on the edge of the plot, since these individuals are closer to the Mediterranean scrubland and thus allow birds to move between habitats with a minimal consumption of energy. On the other hand, both plots are open habitats and have been intensively disturbed by humans in the past, but with some decades apart [16, 41]. Thus, study plots are in different stages of ecological succession and the vegetation surrounding them is different too. For instance, in Matasgordas' plot there are more scattered trees (mostly *Q. suber* and *O. europaea* var. *sylvestris*) than in Reserva's plot. The reduced number of

feces and seeds and the lack of spatial patterns found in Matasgordas could relate to the bird's intensive usage of such scattered trees. A recent study has shown that in places where ecological succession is more advanced (Reserva) the "nursing" effect of *C. humilis* may be diluted among different shrub species [4], since the landscape is dominated by shrub-encroached communities. The "perching" effect of *C. humilis,* however, seems to follow the opposite trend, being enhanced in the most shrub-encroached landscape (i.e. Reserva) probably because of the few available trees for perching.

Interestingly, we found that most of dispersed seeds were concentrated in a small number of *C. humilis* individuals that acted as "hotspots" of seed arrival. For example, in Reserva there was a single large *C. humilis* individual receiving 47.4% (N = 485) of the total seeds found in that plot. Such a strong concentration of dispersed seeds within a few *C. humilis* individuals likely explains that fact that we did not find further spatial pattern of the number of dispersed seeds. Features (other than spatial patterns) that make particular *C. humilis* individuals hotspots of seed arrival are discussed below.

Palm trait's influence on perching effect

This investigation identified some features that made particular *C. humilis* attractive "perching" sites for frugivorous birds and, therefore, hotspots for seed arrival. However, this pattern was found only in Reserva's plot, and not in Matasgordas' plot. In Reserva, larger *C. humilis* individuals received more seed as compared to small ones. The greater surface of large individuals could attract more birds since it implies a greater number of entry points to *C. humilis* interior, where the bird could be safe from predators and adverse weatherly conditions (e.g. high temperatures, rainfall).

The use of *C. humilis* as "perching" site by birds does not necessarily implies seed arrival since not all recorded birds are seed dispersers [66]. However, we sampled during the seed dispersal season, time when some birds change their diet into a more fruit-based one. For instance,

it has been reported that *P. ochruros*, *S. atricapilla* and *S. malenocephala* are able to quickly change their diet when fleshy fruits become available, while other species such as *S. rubicola* show a more gradual change to a frugivorous diet [67, 68]. Also, *S. unicolor, P. ochruros*, *P. collybita*, *P. pica*, and *S. rubicola* has been reported as fruit-eater, although in lesser quantities [69, 70, 71, 72, 73]. Therefore, although not all recorded bird species contribute equally to seed dispersal, fleshy fruits represent a considerable fraction of most of their diets during the seed dispersal season.

Therefore, although not all recorded bird species contribute equally to seed dispersal, fleshy fruits represent a considerable fraction of their diet during this the seed dispersal season.

Secondly, the preference of taller *C. humilis* by frugivorous birds could relate to their own requirements as well as to characteristics of the surrounding landscape. Taller *C. humilis* could suppose safer places since they would provide greater visual fields to birds to spot potential predators. Also, taller perches are likely better sites to take flight towards other habitats (e.g. the Mediterranean scrubland) adjacent to the studied site, which may be more suitable habitats for them [64, 75, 76].

The detected positive effect of richness of beneficiary species within *C. humilis* in Reserva could be related to some of them producing fleshy fruits (e.g. *Rubus ulmifolius*, *Asparagus aphyllus*), acting thus as an additional attractant for frugivorous birds [77, 78, 79]. Richness of beneficiaries was also found positively related to the number of seeds found on *C. humilis*, which is likely a consequence of the increased number of frugivorous bird visits. Additionally, beyond the fact of bearing fleshy fruits, the own beneficiary species could be used as a perch and thus attract birds. Therefore, a similar reasoning explained earlier about how *C. humilis* height influences seed dispersal, could be applied for the revealed positive effect of height of beneficiary plants. Finally, it seemed that male *C. humilis* were more likely to receive dispersed seeds than females. We speculate that male *C. humilis* could be selected by birds over females because the former have a lower associated predation risk. Females *C. humilis* produce fruits that are too big for birds, but they are often eaten by medium-sized carnivores such as the Eurasian badger and

the red fox [33]. Although these mammals are mainly nocturnal, their presence likely prevents birds from using female *C. humilis* in fruit as shelter at night, and thus may dissuade them at day too. The use of *C. humilis* in ecological restoration should follow a balance between male and female plants. Male plants would attract preferably birds that would disperse seeds of woody species present in the surrounding area, while female plants would attract medium-sized carnivores and ungulates that would mostly disperse seed of species such as *P. bourgaena* and *C. humilis*.

In summary, taller (and isolated) male *C. humilis* with a higher diversity of beneficiary species seemed to be the most suitable for attracting frugivorous birds and, therefore, received higher number of dispersed seeds. However, this pattern was not entirely confirmed in Matasgordas' plot, likely because of a much smaller sample sizes (i.e. lesser numbers of both *C. humilis* used as perches and of dispersed seeds).

Implications for ecological restoration

In the last 30 years, restoration ecology has undergone considerable development thanks to the emerging new tools and techniques that facilitate the retrieval of biological properties of disturbed habitats [80]. Our study plots are a good representation of Mediterranean old fields, which have been targets for habitat restoration [5, 16]. Previous studies have demonstrated the value of *C. humilis* in these programs since it facilitates recruitment of numerous woody species dispersed by frugivores [4, 36]. Here, we have demonstrated that *C. humilis* also exerts a marked "perching" effect, crucial in restoration and ecological succession [27, 52, 81, 82]. The combination of artificial and natural perches with nurse plants could increase the seed arrival and recruitment leading to nucleation processes around them [4, 19, 20]. In this sense, to our knowledge, this study represents the first spatially explicit assessment of the relationship between natural perches and seed arrival via frugivorous birds in Mediterranean human-disturbed

landscapes. Our results could be used by managers to decide, for example, whether particular areas are adequate to promote seed arrival using natural or artificial perches.

Finding that dispersed seeds are concentrated in a few *C. humilis* individuals, which tend to be isolated from the rest, breaks surprisingly with the general trend of revegetating under an aggregation design (e.g. [64, 65]. Thus, ideas that were taken for granted in the past have changed thanks to further study and understanding (see [16]). For instance, traditionally, most of shrubs were considered as potential competitors for tree species in habitat restoration [83] and, therefore, as retarders of ecological succession, meaning that they were frequently removed from the area before the revegetation started [6]. This idea has changed during the last few decades and now it is widely known that many shrub species, under different circumstances, can act as nurses for late-successional species, remarking their important role in ecological restoration [28, 62, 84, 85, 86]. Furthermore, the pre-existing vegetation does not act only on flora, but also on the environment conditions (e.g. by reducing soil erosion) making restoration programs more affordable from an economic perspective [23, 63]. Thus, the strategy of using aggregated perches for increasing seed arrival and habitat restoration should not be advocated under all ecological circumstances.

Final conclusions

This investigation supports previous studies about the importance of the pioneer *C. humilis* for the restoration of human-disturbed Mediterranean landscapes. We prove for the first time its key role as natural perch, an ecological function that, though it is shared with some fleshy-fruited woody plants, is especially relevant in early stages of ecological succession when few pioneer species occur. Birds tended to disperse seeds into a small number of *C. humilis* individuals (usually male and large-sized ones) that acted as “hotspots” of seed arrival. Thanks to the double role of *C. humilis*, which attracts frugivorous birds that deliver considerable numbers of seeds of late-successional woody species and provides improved conditions for seedling emergence and

survival, it is likely to become a successful tool for ecological restoration in many habitats of the western Mediterranean basin.

References

1. Asner GP, Elmore AJ, Olander LP, Martin RE, Harris AT. Grazing systems, ecosystem responses, and global change. Annual Review of Environment and Resources 2004;29:261-99.
2. Foley JA, DeFries R, Asner GP, Barford C, Bonan G, Carpenter SR, et al. Global consequences of land use. Science 2005;309:570-574.
3. FAO (Food and Agriculture Organization of the United Nations) [Internet]. FAOSTAT; 2021 [updated 2021 June 29; cited 2021 June 29] Available from: http://faostat.fao.org/site/377/default.aspx
4. Garrote PJ, Castilla AR, Fedriani, JM. The endemic Mediterranean dwarf palm boosts the recolonization of old fields: Implications for restoration. Journal of Environmental Management 2019;250:109478.
5. Rey-Benayas JM, Martins A, Nicolau JM, Schulz JJ. Abandonment of agricultural land: an overview of drivers and consequences. Perspectives in Agriculture, Veterinary Science, Nutrition and Natural Resources 2007;2(57):1-14.
6. Rey PJ, Siles G, Alcántara JM. Community-level restoration profiles in Mediterranean vegetation: nurse-based vs. traditional reforestation. Journal of Applied Ecology 2009;46:937-945.
7. Brunet J. Plant colonization in heterogeneous landscapes: an 80-year perspective on restoration of broadleaved forest vegetation. Journal of Applied Ecology 2007;44:563-572.
8. Cramer VA, Hobbs RJ, Standish RJ. What's new about old fields? Land abandonment and ecosystem assembly. Trends in Ecology & Evolution 2008;23:104-112.
9. Bullock JM, Moy IL, Pywell RF, Coulson SJ, Nolan AM, Caswell H. Plant dispersal and colonization processes at local and landscape scales. In: Bullock JM, Kenward RE, Hails RS, editors. Dispersal Ecology. Oxford, UK: Blackwell Science; 2002. p. 279-302.
10. Rey-Benayas, J. M. Growth and mortality in Quercus ilex L. seedlings after irrigation and artificial shading in Mediterranean set-aside agricultural lands. Annals of Forest Science 1998;55:801-807.
11. Rey-Benayas JM, Navarro J, Espigares T, Zavala MA, Nicolau JM. Effects of artificial shading and weed mowing in reforestation of Mediterranean abandoned cropland with contrasting Quercus species. Forest Ecology and Management 2005;212:302-314.
12. Mouillot F, Ratte JP, Joffre R, Moreno JM, Rambal S. Some determinants of the spatio-temporal fire cycle in a Mediterranean landscape (Corsica, France). Landscape Ecology 2003;18:665-674.
13. Lindskog P, Mando A. (1992) *The relationship between research institutes and NGOs in the field of soil and water conservation in Burkina Faso.* Drylands Issue Paper Nº 39. International Institute for Environment and Development (IIED) London, UK.

14. Vogel, H. Soil erosion interrace farming: destruction of farming land induced by changes in land use in the Haraz mountains of Yemen. Eschborn, Germany. Deutsche Gesellschaft für Technische Zusammenarbeit (GIZ); 1992. 190 p.

15. Levine JM, Murrell DJ. The Community-Level Consequences of Seed Dispersal Patterns. Annual Review of Ecology, Evolution, and Systematics 2003;34(1):549-574.

16. Fedriani JM, Wiegand T, Ayllón D, Palomares F, Suárez-Esteban A, Grimm V. Assisting seed dispersers to restore oldfields: An individual-based model of the interactions among badgers, foxes and Iberian pear trees. Journal of Applied Ecology 2018;55(2):600-611.

17. Debussche M, Escarré J, Lepart J. Ornithochory and plant succession in Mediterranean abandoned orchards. Vegetatio 1982;48:255-266.

18. Howe HF, Smallwood J. Ecology of Seed Dispersal. Annual Review of Ecology and Systematics 1982;13(1):201-228.

19. Pausas JG, Bonet A, Maestre FT, Climent A. The role of the perch effect on the nucleation process in Mediterranean semi-arid oldfields. Acta Oecologica 2006;29(3):346-352.

20. Verdú M, García-Fayós P. Nucleation processes in Mediterranean bird-dispersed plant. Functional Ecology 1996;10:275-280.

21. Izhaki I, Walton PB, Safriel UN. Seed shadows generated by frugivorous birds in an Eastern Mediterranean scrub. Journal of Ecology 1991;79:575-590.

22. Herrera CM, Jordano P, López-Soria L, Amat JA. Recruitment of a mast-fruiting, bird-dispersed tree: bridging frugivore activity and seedling establishment. Ecological Monographs 1994;64:314-344.

23. Castro J, Zamora R, Hódar JA, Gómez JM, Gómez-Aparicio L. Benefits of Using Shrubs as Nurse Plants for Reforestation in Mediterranean Mountains: A 4-Year Study. Restoration Ecology 2004;12(3):352-358.

24. Connell JH, Slatyer RO. Mechanisms of Succession in Natural Communities and Their Role in Community Stability and Organization. The American Naturalist 1977;111(982):1119-1144.

25. Cruz-Alonso V, Villar- Salvador P, Ruiz- Benito P, Ibáñez I, Rey- Benayas JM. Long-term dynamics of shrub facilitation shape the mixing of evergreen and deciduous oaks in Mediterranean abandoned fields. Journal of Ecology 2019;108(3):1125-1137.

26. Debussche M, Lepart J. Establishment of woody plants in mediterranean old fields: opportunity in space and time. Landscape Ecology 1992;6:133-145.

27. Lamb P, Erskine P, Parrota J. Restoration of Degraded Tropical Forest Landscapes. Science 2005;310:1628-1632.

28. Garrote PJ, Castilla AR, Fedriani JM. Assessing the relative importance of nurse species on Mediterranean human- altered areas. Restoration Ecology 2021;29(4):e13402.

29. Wiegand T, Moloney KA. *Handbook of spatial point-pattern analysis in ecology*. Boca Raton, FL, USA: Chapman & Hall/CRDC applied environmental statistics. CRC Press/Taylor & Francis; 2014. 538 p.

30. López-González GA. Los árboles y arbustos de la Península Ibérica e Islas Baleares. 2nd ed. Madrid, Spain: Ediciones Mundi-Prensa; 2006. 1731 p.

31. Guzmán B, JM Fedriani, Delibes M, Vargas P. The colonization history of the Mediterranean dwarf palm (*Chamaerops humilis* L., Palmae). Tree Genetics and Genomics 2017;13:1-10.

32. Charco J, Becerra M, Santa-Barbara C, Fernández C, García FJ, Triano E, Vizoso MT, Baena L. Árboles y arbustos autóctonos de Andalucía. Valencia, Spain: Centro de Investigaciones Ambientales del Mediterráneo; 2014. 593 p.

33. Fedriani JM, Ferreras P, Delibes M. Dietary response of the Eurasian badger, Meles meles, to a decline of its main prey in the Doñana National Park. Journal of Zoology 1998;245:214-218.

34. Castañeda I, Fedriani JM, Delibes M. Red deer (Cervus elaphus) disperse viable seeds spitting from the cud. Mammalian Biology 2018;90:89-91.

35. Selwyn M, Garrote PJ, Castilla AR, Fedriani JM. Interspecific interactions among functionally diverse frugivores and their outcomes for plant reproduction: A new approach based on camera-trap data and tailored null models. PloS one 2020;15(10):e0240614.

36. García-Novo F, Escudero-García JC, Carotenuto L, García-Sevilla D, Fernández-Lo Faso RP. The restoration of El Partido stream watershed (Doñana Natural Park): A multiscale, interdisciplinary approach. Ecological Engineering 2007;30(2):122-130.

37. Soliveres L, DeSoto L, Maestre FT, Olano JM. Spatio-temporal heterogeneity in abiotic factors modulate multiple ontogenetic shifts between competition and facilitation. Perspectives in Plant Ecology 2010;12(3):227-234.

38. Fedriani JM, Wiegand T. Hierarchical mechanisms of spatially contagious seed dispersal in complex seed-disperser networks. Ecology 2014;96(2):514-526.

39. Allier C, González-Bernáldez F, Ramírez-Díaz L. Mapa ecológico de la Reserva Biológica de Doñana. Sevilla. Spain: Consejo Superior de Investigaciones Científicas; 1974. 7 p.

40. Martín R, Rodríguez A, Delibes M. Local feeding specialization by badgers (Meles meles) in a mediterranean environment. Oecologia 1995;101:45-50.

41. Granados-Corona M, Martin-Vicente A, García Novo F. Long-term vegetation changes on the stabilized dunes of Doñana National Park (SW Spain). Vegetatio 1998;75(1-2): 73–80.

42. Garrote PJ, A Castilla, JM Fedriani. Coping with changing plant-plant interactions in restoration ecology: effect of species, site, and individual variation. Applied Vegetation Sciences. 2022;25(1): e12644.

43. Illian J, Penttinen A, Stoyan H, Stoyan D. Statistical analysis and modelling of spatial point patterns. Hoboken, NJ, USA: John Wiley & Sons; 2008. 560 p.

44. Jacquemyn H, Endels P, Honnay O, Wiegand T. Spatio-temporal analysis of seedling recruitment, mortality and persistence into later life stages in the rare Primula vulgaris. Journal of Applied Ecology 2010;47: 431-440.

45. Raventós J, Wiegand T, De Luis M. Evidence for the spatial segregation hypothesis: a test with nine-year survivorship data in a Mediterranean fire-prone shrubland show that interspecific and density-dependent spatial interactions dominate. Ecology 2010;91:2110-2120.

46. Stoyan D, Stoyan H. Fractals, random shapes and point fields: methods of geometrical statistics (Vol. 302). Hoboken, NJ, USA: Wiley-Blackwell; 1994. 408p.

47. Wiegand T, Moloney KA. Handbook of spatial point-pattern analysis in ecology. New York, NY, USA: Chapman and Hall/CRC. 2014. 540p.

48. Getzin S, Worbes M, Wiegand T, Wiegand K. Size dominance regulates tree spacing more than competition within height classes in tropical Cameroon. Journal of Tropical Ecology 2011;27:93-102.

49. Fedriani JM, Wiegand T, Calvo G, Suárez-Esteban A, Jácome M, Żywiec M, et al. Unravelling conflicting density- and distance-dependent effects on plant reproduction using a spatially explicit approach. Journal of Ecology 2015;103:1344-1353.

50. Burnham KP, Anderson DR. Model selection and multimodel inference: a practical information-theoretic approach. 2nd ed. New York, USA: *Springer-Verlag*; 2002. 488 p.

51. R Core Team. R: A language and environment for statistical computing. Vienna, Austria: R Foundation for Statistical Computing, 2021. Available from: https://www.R-project.org/.

52. Graham LLB, Page SE. Artificial bird perches for the regeneration of degraded tropical peat swamp forest: a restoration tool with limited potential. Restoration Ecology 2012;20:631-637.

53. La Mantia T, Rühl J, Massa B, Pipitone S, Lo Verde G, Bueno RS. Vertebrate-mediated seed rain and artificial perches contribute to overcome seed dispersal limitation in a Mediterranean old field. Restoration Ecology 2019;27(6):1393-1400.

54. Zanini L, Ganade G. Restoration of Araucaria Forest: The Role of Perches, Pioneer Vegetation, and Soil Fertility. Restoration Ecology 2005;13(3):507-514

55. Pegman APM, Perry GLW, Clout MN. Exploring the interaction of avian frugivory and plant spatial heterogeneity and its effect on seed dispersal kernels using a simulation model. Ecography 2017;40:1098-1109.

56. Bastida F, Talavera S. (2002) Temporal and Spatial Patterns of Seed Dispersal in Two Cistus Species (Cistaceae). Annals of Botany 2002;89(4):427-434.

57. Talavera S, Gibbs PE, Herrera J. Reproductive biology of Cistus ladanifer (Cistaceae). Plant Systematics and Evolution 1993;186:123-134.

58. Melendo M, Giménez E, Cano E, Gómez-Mercado F, Valle F. The endemic flora in the south of the Iberian Peninsula: taxonomic composition, biological spectrum, pollination, reproductive mode and dispersal. Flora. Morphology, Distribution, Functional Ecology of Plants 2003;198(4):260-276.

59. Fedriani JM, Delibes M. Seed dispersal in the Iberian pear, Pyrus bourgaeana: A role for infrequent mutualists. Écoscience 2009;16(3):311-321.

60. Carlo TA, Morales JM. Inequalities in fruit-removal and seed dispersal: consequences of bird behavior, neighborhood density and landscape aggregation. Journal of Ecology 2008;96(4):609-618.

61. Howe HF. Fear and frugivory. American Naturalist, 1979;114:925-931.

62. Callaway RM, Pugnaire FI. Facilitation in plant communities. In Pugnaire FI, Valladares F, editors. *Handbook of functional plant ecology*. New York, USA: Marcel Dekker; 1999. p. 623-648.

63. Gómez-Aparicio L, Zamora R, Gómez JM, Hódar JA, Castro J, Baraza E. Applying plant facilitation to forest restoration: a meta-analysis of the use of shrubs as nurse plants. Ecological Applications 2004;14(4):1128-1138.

64. Pueyo Y, Moret-Fernández D, Saiz H, Bueno CG, Alados CL. Relationships Between Plant Spatial Patterns, Water Infiltration Capacity, and Plant Community Composition in Semi-arid Mediterranean Ecosystems Along Stress Gradients. Ecosystems 2013;16:452-466.

65. Rey-Benayas JM, Bullock JM, Newton AC. Creating woodland islets to reconcile ecological restoration, conservation, and agricultural land use. Frontiers in Ecology and the Environment 2008;6:329-336.

66. Schupp EW, Jordano P, Gómez JM. Seed dispersal effectiveness revisited: a conceptual review. New Phytologist 2010;188(2):333-353.

67. Campo-Celada M, Jordano P, Benítez-López A, Gutiérrez-Expósito C, Rabadán-González J, Mendoza I. Assessing short and long-term variations in diversity, timing and body condition of frugivorous birds. Oikos 2022;2:e08387

68. Carnicer J, Jordano P, Melían CJ. The temporal dynamics of resource use by frugivorous birds: a network approach. Ecology 2009;90(7):1958-1970.

69. Jordano P. Avian Fruit Removal: Effects of Fruit Variation, Crop Size, and Insect Damage. Ecology, 1987:68(6):1711-1723.

70. Carnicer J, Jordano P, Melían CJ. The temporal dynamics of resource use by frugivorous birds: a network approach. Ecology, 2009;90(7):1958-1970.

71. Gonzalez-Varo JP, Carvalho CS, Arroyo JM, Jordano P. Unravelling seed dispersal through fragmented landscapes: Frugivore species operate unevenly as mobile links. Molecular Ecology, 2017;26(16):4309-4321.

72. Green AJ, Elmberg J, Lovas-Kiss A. Beyond Scatter-Hoarding and Frugivory: European Corvids as Overlooked Vectors for a Broad Range of Plants. Front. Ecol. Evol., 2019;7(133).

73. Campo-Celada M, Jordano P, Benítez-López A, Gutiérrez-Expósito C, Rabadán-González J, Mendoza I. Assessing short and long-term variations in diversity, timing and body condition of frugivorous birds. Oikos, 2022;2:e08387

74. Vergara PM. Matrix-dependent corridor effectiveness and the abundance of forest birds in fragmented landscapes. Landscape Ecology, 2011;26:1085

75. Askins RA, Folsom-O'Keefe CM, Hardy MC. Effects of Vegetation, Corridor Width and Regional Land Use on Early Successional Birds on Powerline Corridors. PLoS ONE 2012;7(2):e31520.

76. Piratelli AJ, Piña-Rodrigues FCM, Raedig C. Integrating Biodiversity Conservation into Agroecosystem Management: Using Birds to Bring Conservation and Agricultural Production Together. In: Nehren U, Schlüter S, Raedig C, Sattler D, Hissa H, editors. *Strategies and Tools for a Sustainable Rural Rio de Janeiro*. Cham, Switzerland: Springer; 2019. P. 139-153.

77. Rey PJ. Preserving frugivorous birds in agro-ecosystems: lessons from Spanish olive orchards. Journal of Applied Ecology 2011;48:228-237.

78. Kaur N, Dhanju CK. Food and Feeding Habits of Common Birds of Agroecosystems. Indian Journal of Ecology 2013;40(1):83-86.

79. Morgado R, Pedroso R, Porto M, Herrera JM, Rego F, Moreira F, Beja P. Preserving wintering frugivorous birds in agro-ecosystems under land use change: Lessons from intensive and super-intensive olive orchards. Journal of Applied Ecology 2021;58(12):2975-2986.

80. Van Andel J, Aronson J. Restoration Ecology: The New Frontier. Oxford, UK: Blackwell Scientific Publications; 2006. 400 p.

81. Martínez-López V, Zapata V, De la Rúa P, Robledano F. Uncovering mechanisms of bird seed dispersal in semiarid environments to help to restore them. Ecosphere 2019;10(4):e02673.

82. Shiels AB, Walker LR. Bird perches increase forest seeds on Puerto Rican landslides. Restoration Ecology 2003;11(4):457-465.

83. Mesón M, Montoya M. Selvicultura Mediterránea. Madrid, Spain: Ediciones Mundi Prensa; 1993. 368 p.

84. Gómez-Aparicio L. The role of plant interactions in the restoration of degraded ecosystems: a meta-analysis across life-forms and ecosystems. Journal of Ecology 2009;97:1202-1214.

85. Jankju M. Role of nurse shrubs in restoration of an arid rangeland: Effects of microclimate on grass establishment. Journal of Arid Environments 2013;89:103-109.

86. Padilla FM, Pugnaire FI. The role of nurse plants in the restoration of degraded environments. Frontiers in Ecology and the Environment 2006;4(4):196-202.

Figure 1

Click here to access/download;Figure;Fig1.tif

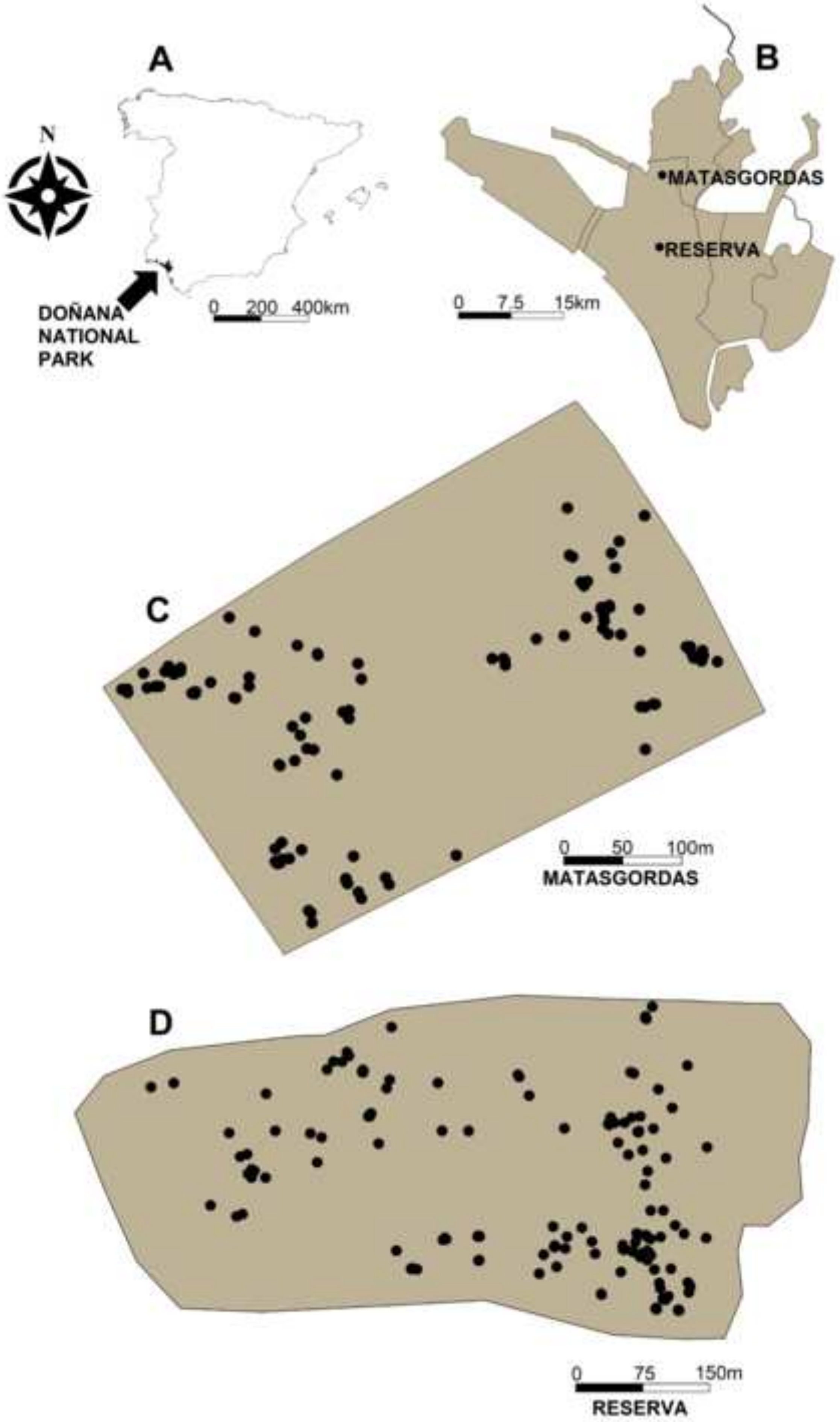

Figure 2

Click here to access/download;Figure;Fig2.tif

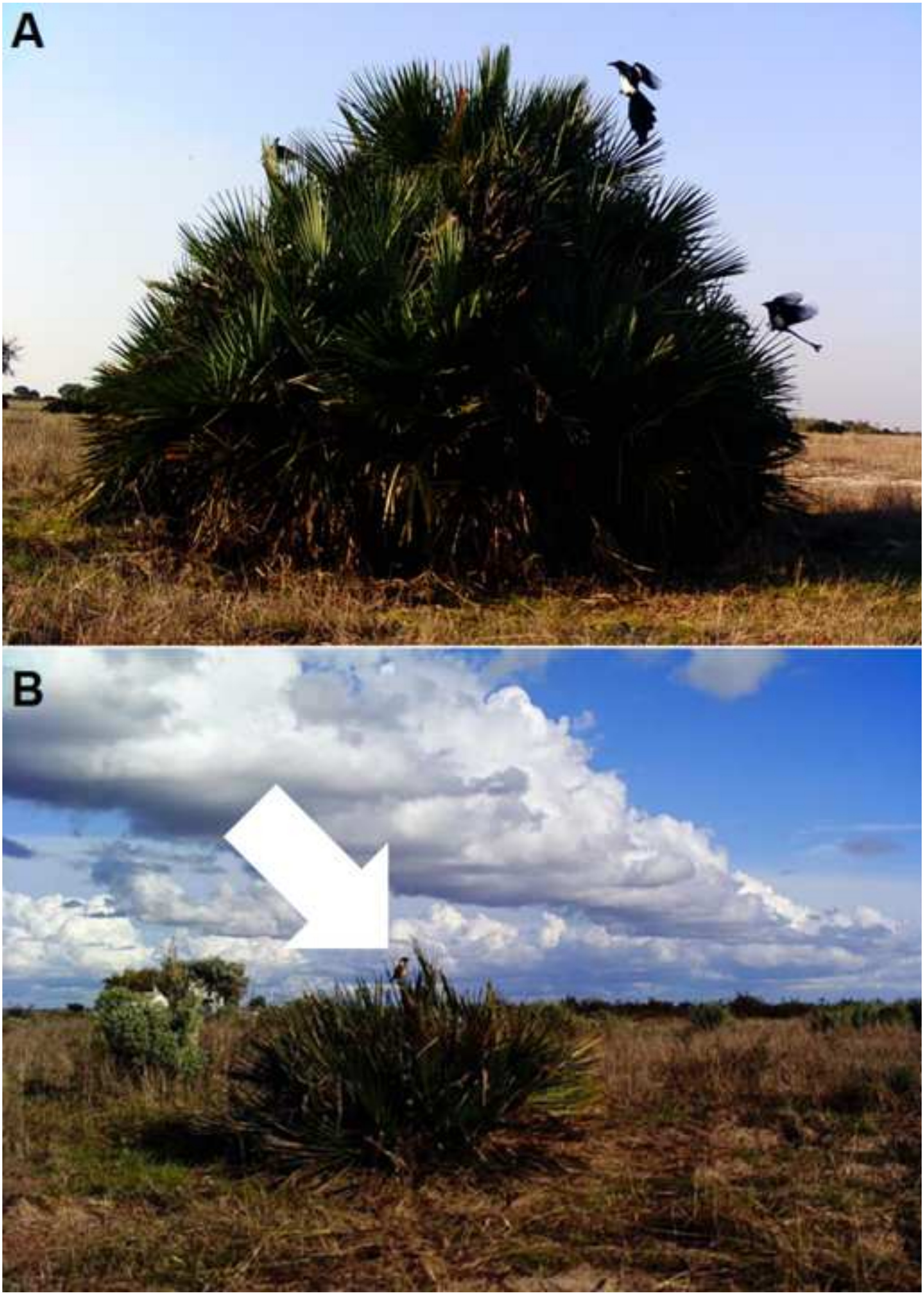

Figure 3

Click here to access/download;Figure;Fig3.tif

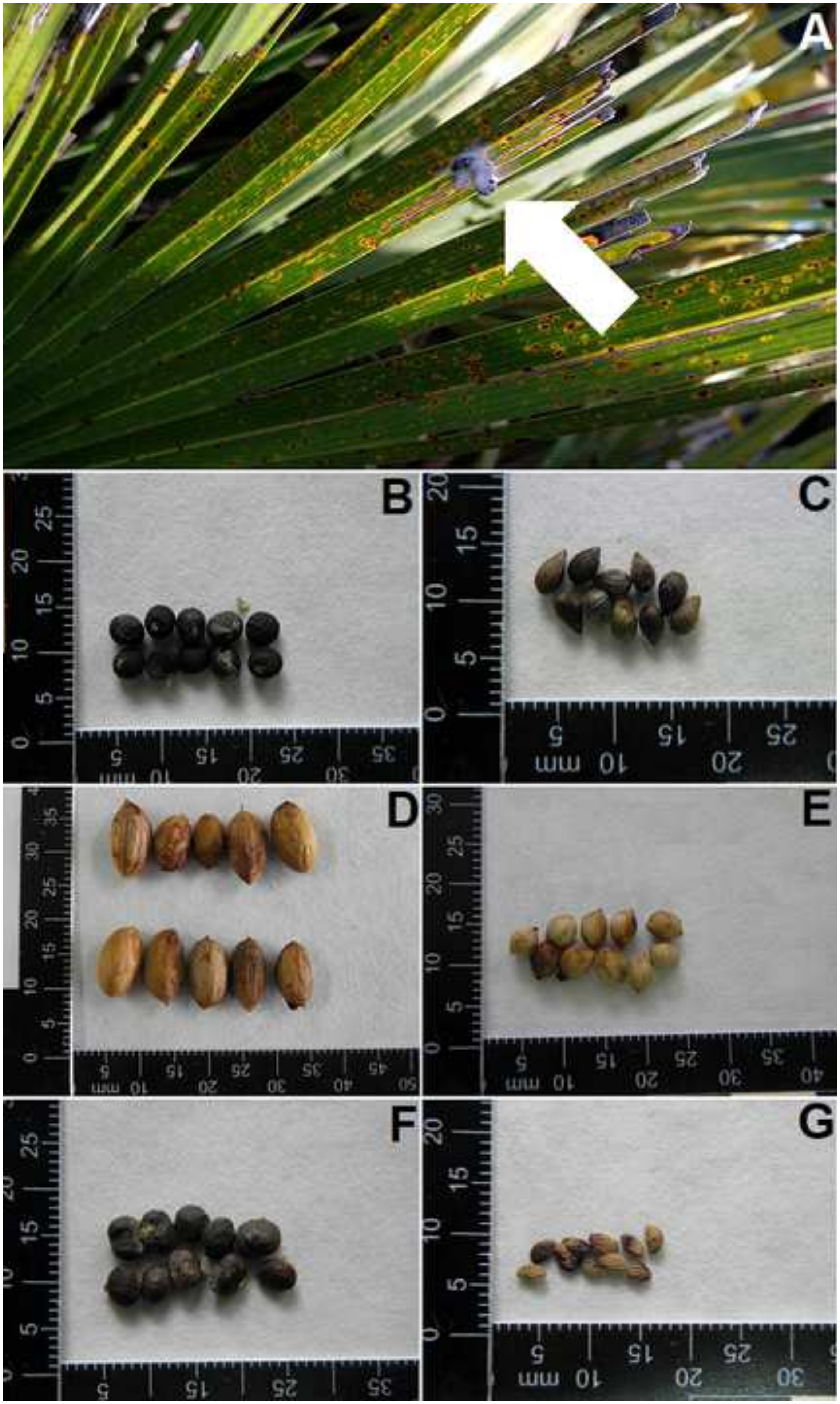

Figure 4

Click here to access/download;Figure;Fig4.tif

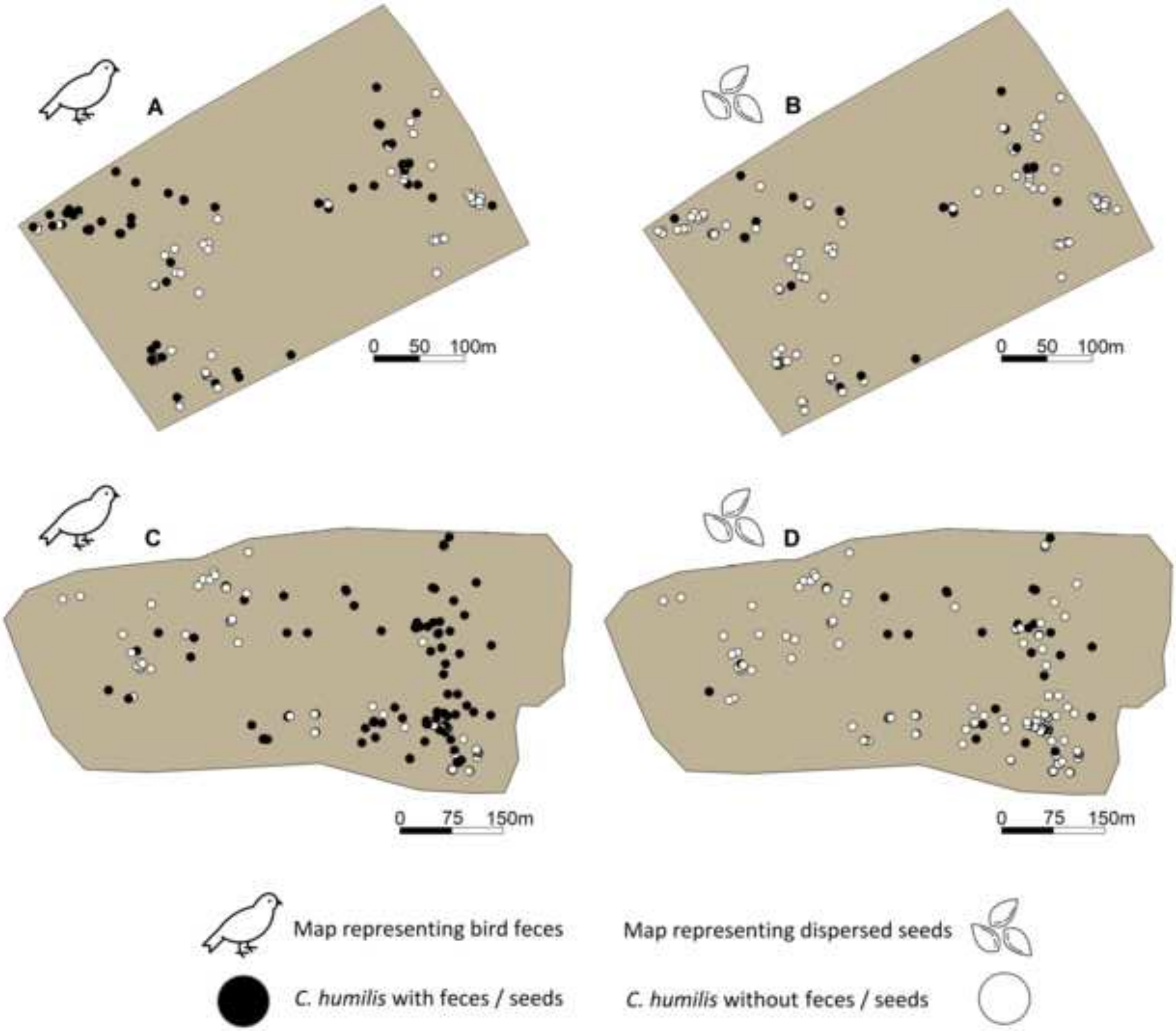

Figure 5

Click here to access/download;Figure;Fig5.tif

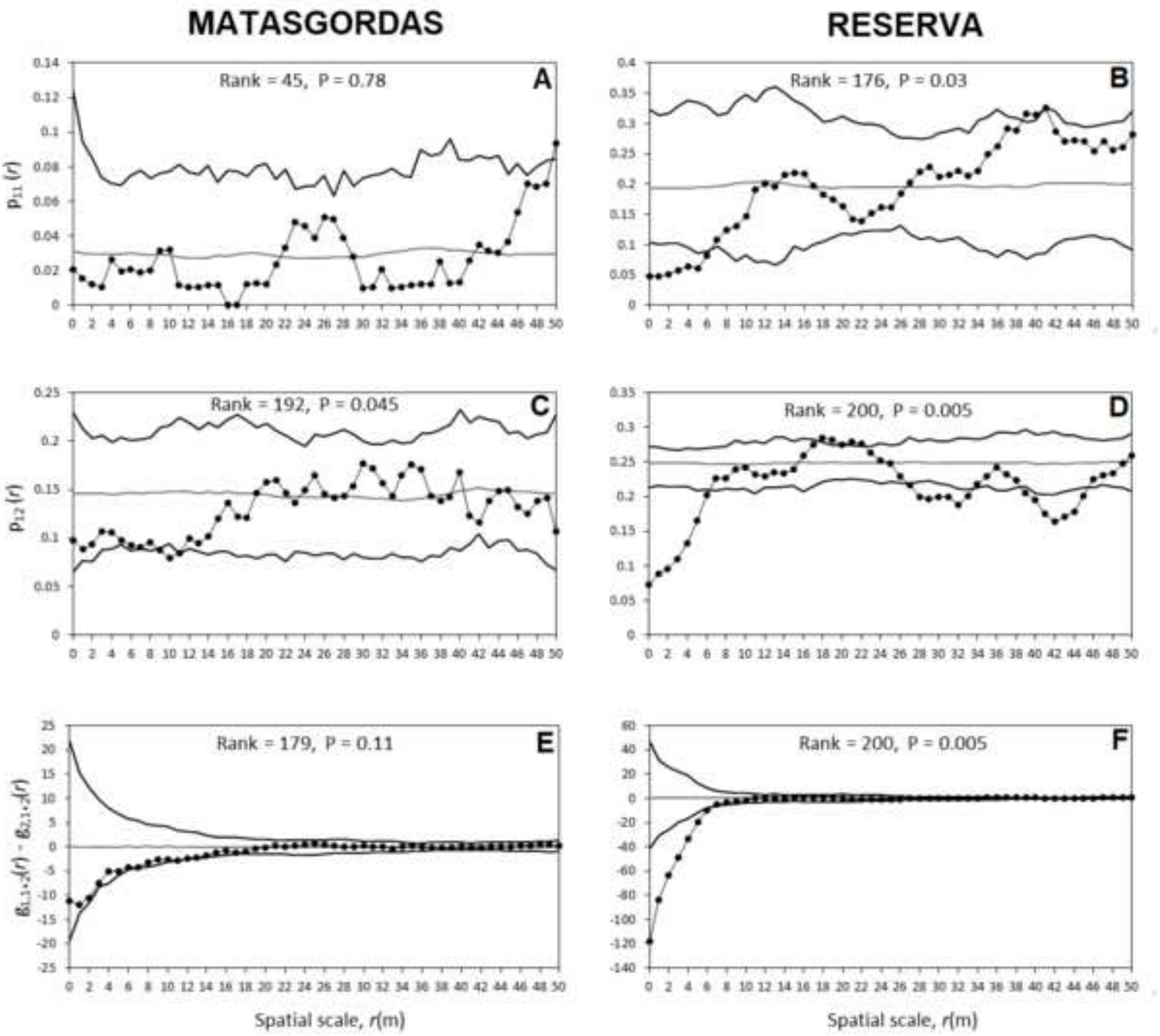

Figure 6

Click here to access/download;Figure;Fig6.tif

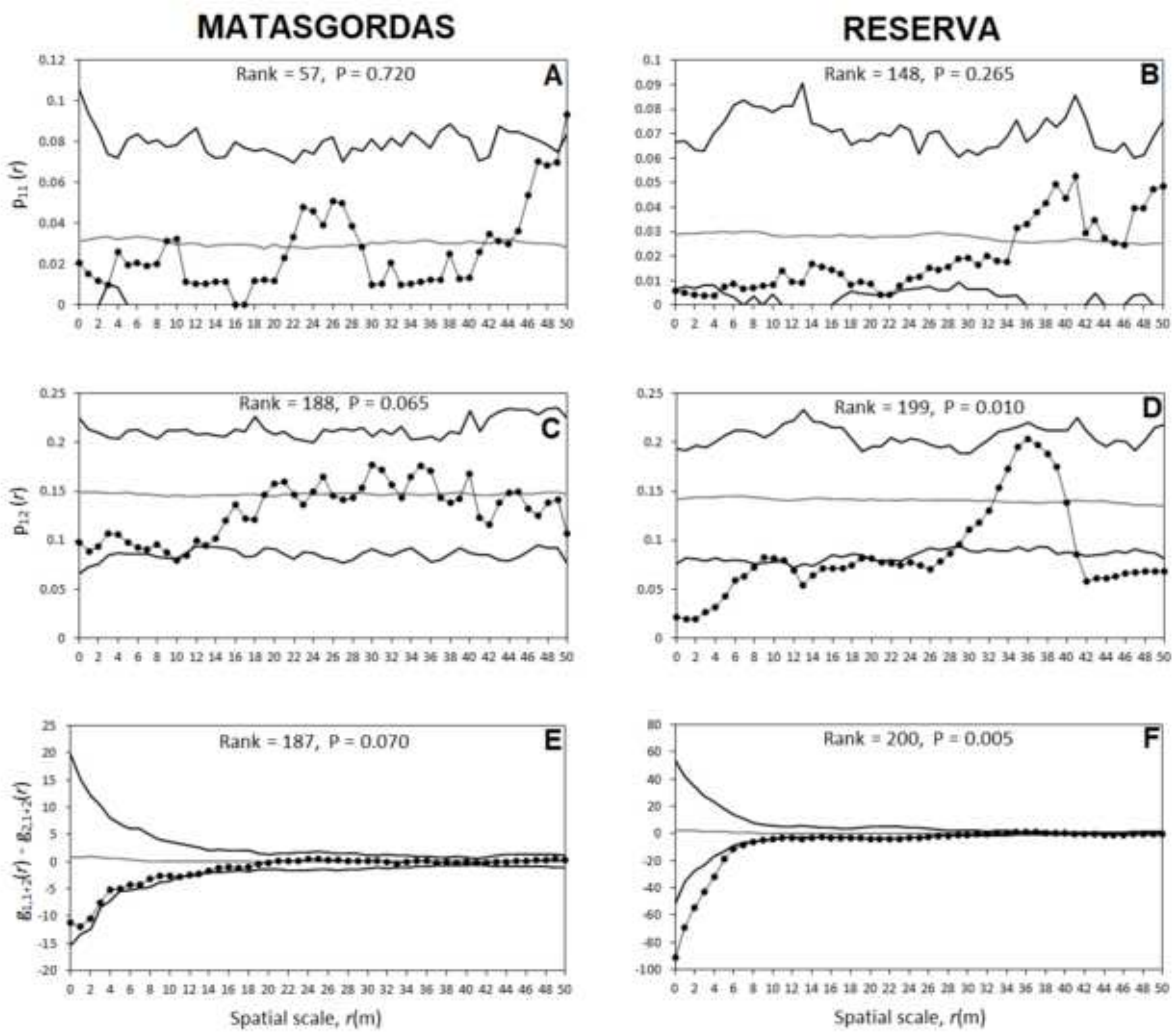

Figure 7

Click here to access/download;Figure;Fig7.tif

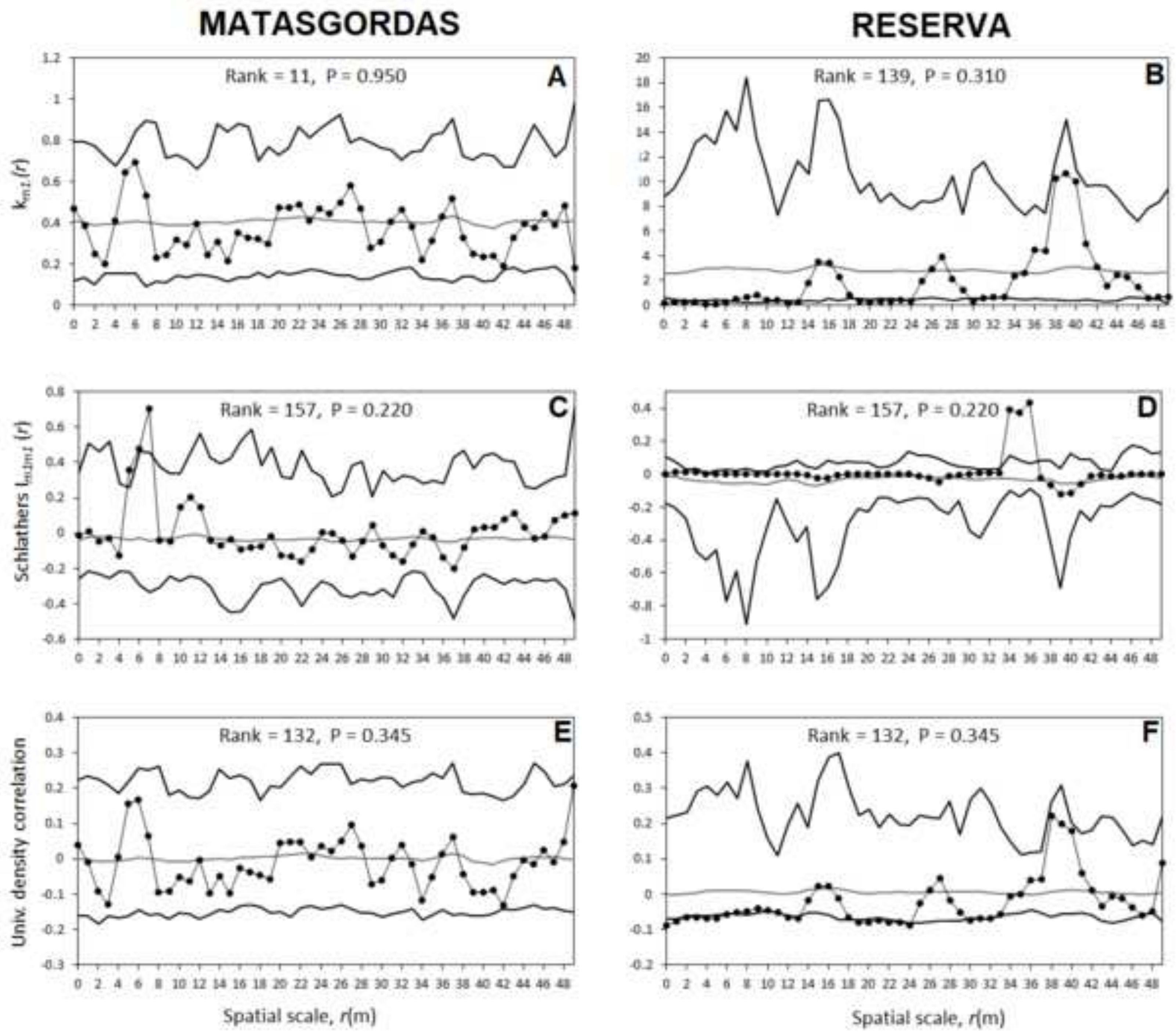

Click here to access/download
Supporting Information
Supporting information.docx

Unmasking the perching effect of the pioneer Mediterranean palm *Chamaerops humilis* L.

Víctor González-García[1]* ¶, Pedro J. Garrote[2] ¶ and Jose M. Fedriani[3, 4] *¶

[1] Atlantic Botanical Gardens, Gijon, Asturias, Spain

[2] Centre for Applied Ecology "Prof. Baeta Neves" (CEABN/InBIO), Institute Superior of Agronomy, University of Lisbon, Tapada da Ajuda, Lisbon, Portugal

[3] Desertification Research Centre CIDE, CSIC UV GV, Moncada, Valencia, Spain

[4] Doñana Biological Station (EBD - CSIC), Seville, Spain

* Corresponding authors

victor.gon.gar@hotmail.com (VG), fedriani@csic.es (JMF)

¶ These authors contributed equally to this work.

Abstract

Although farmlands are the most extended terrestrial biomes, the abandonment of traditional agriculture in many parts of world has brought challenges for the restoration of such human-disturbed habitats. Seed arrival is a crucial necessary ecological process during the (re)colonization that can be enhanced by the use of the so-called "perch plants". Little is known about how spatial aspects of "perching" affect their effectiveness in disturbed habitats. To evaluate several spatial aspects of "perching" effect, including whether the seed arrival via frugivorous birds is associated to the spatial distribution of the perch plants, we used a spatially explicit approach in two disturbed plots within the Doñana National Park (SW Spain). Specifically, we chose as study system the pioneer Mediterranean dwarf palm *Chamaerops humilis* L., which is often used as perch by a variety of frugivorous bird species. A total of 289 *C. humilis* individuals were sampled in search of bird feces (N = 2998) and dispersed seeds (N = 529). Recorded seeds belonged to six different woody species from five different families. Nine bird species from six different families were recorded using *C. humilis* as perches. GLMs analyses indicated that taller *C. humilis* males with high richness of beneficiary woody species received more dispersed seeds. We detected a random spatial structure of bird feces and dispersed seeds in one study plot, while a nonrandom spatial structure was found in the other one, where isolated *C. humilis* received a higher number of bird feces and dispersed seeds than expected under spatial null models. The difference in spatial patterns between both study plots could relate, among other factors, to their different state of development in the ecological succession. Most of dispersed seeds were concentrated in a small number of *C. humilis* individuals (usually male and large ones) that acted as "hotspots" of seed arrival. The fact that frugivorous birds visited most often isolated *C. humilis* (rather than aggregated ones) questions the aggregated spatial structure of revegetation designs typically used in restoration projects. This study reveals novel spatial aspects of the "perching" effect which could be helpful in the restoration of human-disturbed habitats worldwide.

Introduction

Croplands and grazing lands have become the largest terrestrial biomes, occupying almost the 40% of Earth's land surface [1, 2]. However, in the Mediterranean Europe, areas destined to agriculture have been reduced by 34.2% in the last half century. In Spain, specifically, they have decreased by 54.8% [3, 4]. This abandonment of farmlands represents an opportunity to boost biodiversity through natural (and facilitated) revegetation and reforestation [5], and thus to restore habitats to the stages prior to human disturbances. Restoration projects are generally expensive given that they have been usually focused on planting dominant tree species [6]. Although many of such restoration projects have been successfully [7], they could be unaffordable when the affected area is too large, limiting the full recovery of vegetation structure [8]. Natural regeneration acts more widely and is less expensive than tree plantations [5], although its intensity varies by region and habitat. In arid and semiarid areas with low primary productivity such as the Mediterranean basin, natural restoration is clearly slower [7, 9] than in more humid and productive environments like tropics or temperate regions, being the water availability for plants an evident limitation [10]. Some other constrains in human-disturbed habitat restoration are inter- and intraspecific competition from herbaceous plants [11], the border effect or the past landscape pattern [12], and the limited propagule pressure and dispersal capacity of seeds [9, 13, 14].

Seed dispersal plays a key role in habitat dynamics and structure of plant communities [15]. Seed dispersal facilitates plant colonization of vacant areas and thus, to recover habitats that have returned to be suitable [16]. Birds and mammals are the main seed dispersers of most fleshy-fruited plants [17]. Many frugivorous birds often use tree and shrub branches as "perches", i.e. sites where they defecate and regurgitate large number of viable seeds [18, 19, 20]. Repeated visits to the same perches locally increase seed arrival and thus can trigger nucleation processes [20]. Human-disturbed habitats are more prone to these nucleation effects, since they are often vacant habitats where perches are scarce, which promotes a marked seed clumping [21, 22]. However, we are not aware of any study that has quantified the patterns of seed clumping under perches using a spatially explicit approach.

Interestingly, in some cases, the same shrub species acting as perches also act as nurse plants, i.e. facilitating the emergence, growth and survival of other plant species, [23] which are designated as "beneficiary species", promoting the natural (re)colonization [24, 25]. *Chamaerops humilis* L., is a pioneer Mediterranean palm crucial in habitat recovery [26]. Our unpublished data indicate that these palms potentially act as perches for many (frugivorous) birds, which play important roles as seed dispersers and agents of the natural (re)colonization of human-disturbed areas by woody plants (e.g. [27]). The facilitative role of this keystone palm has been recently evaluated [28]. In particular, [4] the dwarf palm has been found positively associated with multiple woody plant species in disturbed vacant habitats. By contrast, the "perching" effect of *C. humilis* and its potential implications for the ecological restoration of human-disturbed landscapes remain unknown. In addition, very little is known about the pervasiveness of the natural "perching" effect and its spatial aspects and scales. On this matter, Spatial Point Pattern Analysis (SPPA) constitute a powerful tool to evaluate this sort of questions [29].

In this study we evaluate spatial patterns on the role of *C. humilis* as perch for frugivorous birds dispersing woody plant seeds in human-disturbed habitats. To achieve our goal, we selected two study plots within Doñana National Park (SW Spain) that differ in their history of human-driven perturbations and vegetation. We used a spatially explicit approach to answer the following questions: (*i*) How pervasive is the "perching" effect in our two studied human-disturbed habitats? (*ii*) Are there differences between the two study plots in the role of *C. humilis* as perch? And, if so, what are the factors underlying such differences? (*iii*) Do aggregated *C. humilis* receive more bird feces comprising dispersed seeds? (*iv*) Do particular *C. humilis* individuals act as "hot spots" of seed arrival, and if so, why? And (*v*) Do the species of dispersed seeds correspond to the woody beneficiary species found spatially associated to the *C. humilis*?

Material and methods

Study Species

Chamaerops humilis L. (Arecaceae), commonly known as "European fan palm" or "Mediterranean dwarf palm", is an evergreen shrub original from Western Mediterranean [30, 31]. This palm can grow up to 10m tall, although individuals are not usually taller than 2m [32]. Leaves are big, fan-shaped and displayed in numerous segments, exhibiting several long thorns on the petiole. It is dioecious, with fruits only growing on female plants. Flowering occurs in spring, from March to May. Spread takes place through seeds (sexually) or, less commonly, through division of adult trees (asexually) [30]. Seeds are mostly dispersed by medium-sized carnivores such as Eurasian badgers *Meles meles* and red foxes *Vulpes vulpes* [33] but also by ungulates (e.g. red deers *Cervus elaphus* or wild boars *Sus scrofa*) some of which occasionally disperse seeds by regurgitation [34, 35].

The facilitative role of *C. humilis* and its capacity of recolonizing disturbed habitats have made it widely used in ecological restorations [36], although its role as nurse plant has only recently been demonstrated [4]. *Chamaerops humilis* protect seeds and seedlings of woody species from high temperatures and drought, as well as from herbivory thanks to the spikes present in its leaves [4, 37]. Frequent visits of some seed dispersers such as red foxes could lessen seed and seedling predation of beneficiary plants by several species of rodents (e.g. *Apodemus sylvaticus*) and lagomorphs (e.g. *Oryctolagus cuniculus*), which use nurse plants as shelters from their predators [35].

In its typical habitat, the Mediterranean scrubland, *C. humilis* is often spatially associated with *Asparagus* L. spp., *Daphne gnidium* L., *Olea europaea* L. var. *sylvestris* (Mill.) Hegi, *Pistacia lentiscus* L. or *Pyrus bourgaeana* Mecde, and other woody species. These spatial associations are thought to be due to a combination of "nursing" and "perching" effects [4, 30], although the "perching" effect has not been tested yet.

Study area and plots

The study was carried out within the Doñana National Park (SW Spain, on the west of the Guadalquivir River estuary). All permits necessary were granted by the National Park Service (ref. 2019/10) and the Junta de Andalucía (ref. 2019107300002261/IRM/MDCG/mes). The National Park presents a Mediterranean sub-humid climate, with most of rainfalls taking place from November to April, showing an average annual rainfall of 500-600mm [33], with hot dry summers (June-September) and template humid winters (October-January).

We selected two study plots 10 km apart called Matasgordas (area sampled: 13.9 ha) and Doñana Biological Reserve (area sampled: 21.4 ha) (Fig 1). Study areas are different in terms of vegetation and human-use history [4, 38]. Matasgordas has been historically and intensively affected by anthropic management of the vegetation and livestock pressure [39]. The eastern portion of this study plot was drastically modified in 1970 when most of woody plants (both shrubs and trees) were removed in order to create a "dehesa", becoming an open area for cattle grazing with scattered trees, mostly *Quercus suber* L. and *O. europaea* var. *sylvestris* [4, 16]. In 1996, the land became owned by Government and happened to be under the protection of the National Park boundaries, putting an end to the cattle grazing. Once livestock was removed from the area, native fleshy-fruited plants such as *C. humilis*, *D. gnidum*, *P. lentiscus* and *P. bourgaeana* started to recolonize their habitat via seed dispersal by birds and mammals [4, 16]. Due to such past human activity, Matasgordas is now formed, mainly, by two habitats: (*i*) Scrubland dominated by *P. lentiscus* shrubs with some *Q. suber* and *O. europaea* var. *sylvestris trees* [4, 33] and (*ii*) an old-field, where our study plot was set, which woody vegetation is mainly composed by animal-dispersed native plants such as *C. humilis, P. lentiscus, D. gnidium* and *P. bourgaeana, Asparagus aphyllus* L., *Halimium halimifolium* (L.) Willk and *Cistus salviifolius* L. and scattered *Q. suber* and *O. europaea* var. *sylvestris* trees [4]. The distribution of *C. humilis* in this plot is very aggregated, and best described by a double-clustered component process with a random component process (with 22% of isolated *C. humilis* individuals; see Supporting Information [S1 Cluster Analysis]).

Figure 1. **The study plots.** (A) Darkened location of the Doñana National Park within (NW) Spain. (B) Doñana Natural Park with the two study plots highlighted in black, being Matasgordas the northern one and Reserva the southern one. (C) Matasgordas and (D) Reserva study plots located within the Doñana National Park. Every occurring *C. humilis* was georeferenced (black solid points).

Doñana Biological Reserve is located in the centre of the National Park. It is laid on a coastal plain distinguished for its flatness and being separated from the sea by lagoons and mobile dune systems. However, it is this closeness to the sea which allows a humid and mild climate [40] and enables the existence of three different types of habitats: marshland, dunes and scrubland; being the ecotone between the scrubland and the marshes (locally known as "La Vera") where we set our study plot. This area has been historically managed by human for agriculture, hunting, cattle grazing and tree felling, especially *O. europaea* var. *sylvestris* and *Q. suber* [41]. Reserva was protected earlier than Matasgordas, in 1964 and it has been recovering ever since, being recolonised by animal-dispersed plants such as *A. aphyllus*, *C. humilis*, *Phillyrea angustifolia* L. or *R. ulmifolius* [4]. This scrubland is dominated by *H. halimifolium* and *Stauracanthus genistoides* (Brot.) Samp. with scattered trees of *Q. suber, O. europaea* var. *sylvestris* and *Pinus pinea* [33]. The study plot at Reserva presents higher density of shrubs than the one at Matasgordas. Such a difference in shrub cover led to a greater abundance of fleshy fruits in Reserva, which likely makes this site more attractive to frugivorous birds [4]. The distribution of *C. humilis* in this plot is very aggregated, and best described by a double-clustered component process with a random component process (with 9% of isolated *C. humilis* individuals; see Supporting Information [S1 Cluster Analysis]).

Data Collection

Every target *C. humilis* (109 and 180 in Matasgordas and Reserva, respectively) was individually georeferenced with a submetric GPS Leica 1200. The density of *C. humilis* plants in Matasgordas' plot (7.78 individuals/ha) was pretty similar than in Reserva' plot (8.41 individuals/ha). Each study plot was visited eight and five times along the seed dispersal seasons (i.e. from September to November) of 2019 and 2020, respectively. To control for potential temporal variations on bird abundance and seed dispersal along the dispersal seasons, both study plots were sampled during the same sampling days. Rainy days were avoided because of the low bird activity and the feces wash out on *C. humilis*.

During the sampling, which lasted about 3h per day in Matasgordas and about 2h in Reserva, any observed bird perching on focal *C. humilis* individuals was recorded. Overall, nine bird species were recorded perching: *Cisticola juncidis*, *Lanius senator*, *Phoenicurus ochruros*, *Phylloscopus collybita*, *Pica pica*, *Saxicola rubicola*, *Sturnus unicolor*, *Sylvia atricapilla* and *Sylvia melanocephala* (Fig 2, Table 1).

Figure 2. Birds using *Chamaerops humilis* as perches in Doñana National Park. (A) Eurasian magpie (*Pica pica*). (B) Common stonechat (*Saxicola rubicola*). (Photo credit: Pedro J. Garrote)

Table 1. **Summary of collected data**.

	MATASGORDAS		RESERVA	
	2019	2020	2019	2020
Chamaerops humilis (individuals)	109		180	
Number of feces	705	206	1560	527
Number of seeds	28	16	340	145
C. humilis with feces (%)	42.2	37.6	43.9	27.8
Feces with seeds (%)	3.3	17.8	7.5	7.8
C. humilis with seeds (%)	8.3	11.9	13.9	7.8
Asparagus aphyllus (seeds)	1	7	16	3
Daphne gnidium (seeds)	7	5	1	2
Olea europaea var. *sylvestris* (seeds)	0	0	26	8
Phillyrea angustifolia (seeds)	0	0	4	0
Pistacia lentiscus (seeds)	20	4	2	0

Rubus ulmifolius (seeds)	0	0	291	132
**Perching birds**				
Cisticola juncidis	1	1	0	2
Lanius senator	0	0	2	0
Phoenicurus ochruros	0	0	3	1
Phylloscopus collybita	0	0	1	0
Pica pica	0	0	2	0
Saxicola rubicola	2	1	7	4
Sturnus unicolor	0	0	2	0
Sylvia atricapilla	1	1	1	2
Sylvia melanocephala	3	3	14	4
**Beneficiary species**				
Asparagus aphyllus	35		96	
Cistus sakviifolius	16		0	
Daphne gnidium	1		0	
Olea europaea var. *sylvestris*	6		20	
Pistacia lentiscus	3		0	
Pyrus bourgaeana	1		0	
Quercus suber	1		5	
Rubus ulmifolius	3		7	
Stauracanthus genistoides	31		17	

Number of samples (feces, seeds) collected in each study plot, the number of seeds found for each plant species, as well as the number and species of bird recorded perching on *C. humilis*.

Every target *C. humilis* was checked for bird feces and regurgitations by carefully checking the palm foliage surface. We also evaluated whether seeds arrive at open spaces without palms. To this aim, we checked for the presence of bird feces and regurgitations in an open area associated to each focal *C. humilis*. These areas were of similar surface and mostly located in bare soils with scarce vegetation (mainly grasses) and 3-4m from the associated *C. humilis*. Every found excrement and regurgitated seed was collected and stored inside of an individual Eppendorf tube. In the lab, feces were dried to make their handling easier. Once dried, every sample was checked for seeds by crumbling the excrement and looking for hard matter, either of vegetable (seeds, bark) or animal (elytra and other arthropod parts) origin. Found seeds were identified at the species level using images of a reference collection (Fig 3), considering its shape, size and the surface design. All collected seeds (N = 529) were positively identified.

Figure 3. Examples of collected feces and identified dispersed seeds. (A) Bird excrement on a leaf of *Chamaerops humilis* in Matasgordas (B) *Asparagus aphyllus.* (C) *Daphne gnidium.* (D) *Olea europaea* var. *sylvestris*. (E) *Phillyrea angustifolia*. (F) *Pistacia lentiscus*. (G) *Rubus ulmifolius*. (Photo credit: Pedro J. Garrote).

To assess the potential selection by birds of particular traits of *C. humilis* individuals, we measured the following palm variables: gender (male, female, undetermined), size (height, area and volume), presence and richness of beneficiary woody species within them and beneficiary height (<25cm, 25-100cm, >100cm). Beneficiary species were woody plants that grow under dwarf palms and emerge from the top of their surface. These species often benefit from *C. humilis* due to microclimatic improvement and protection against herbivory [42].

Spatial analyses

Aggregation of *C. humilis* used as perches within the overall pattern *of C. humilis*

Every *C. humilis* was characterized with its spatial location and a mark (presence/absence of bird feces in this case). To find out if *C. humilis* used as perches by birds (i.e. receiving bird feces) show a spatial pattern (aggregated, isolated) or they instead are randomly distributed we used qualitatively marked point patterns techniques and the random labelling null models to evaluate lack of any spatial structure. The random labelling null model consists in giving the mark "with feces" or "without feces" over all *C. humilis* (having or not feces) randomly, conducting a total of 199 simulations [38].

In order to quantify the spatial pattern of *C. humilis* used as perches within the overall pattern of *C. humilis* we used mark connection functions [43, 44, 45] as summary statistics [38]. Whether *C. humilis* contained feces they were assigned with a mark of value 1 and a mark of

value 0 if they had no feces. The mark connection function $p_{ij}(r)$ gives the probability that two *C. humilis*, separated by a distance *r*, the first is type *i* (i.e. with feces) and the second is type *j* (without feces).

Finally, to test if *C. humilis* with feces are located in areas of overall high density of neighbouring *C. humilis* (with feces or without them) we used the test statistic $g_{i,i+j}(r) - g_{j,i+j}(r)$ [44], which consist in comparing the density of *C. humilis* ($i + j$) around *C. humilis* with feces (type *i*) with the density of *C. humilis* ($i + j$) around *C. humilis* without feces (type *j*). Under random labelling, the expected value for this statistic is zero: in case that feces tend to be delivered in palm aggregations we expected $g_{i,i+j}(r) > g_{j,i+j}(r)$ and, if not, if there is an isolation of feces, the we expected $g_{i,i+j}(r) < g_{j,i+j}(r)$.

To test the fit of data with the point process and departures from the null model, we conducted 199 simulations (for every analysis) of the fitted point processes and estimated envelopes with an approximate error rate of $\alpha = 0.05$ [46] which are the fifth lowest and highest values of the summary statistics of the simulated point process. Observed values above the top or below the bottom simulation envelopes indicate higher or lower than expected spatial aggregation, respectively. Observed values within the simulation envelopes indicate a level of spatial aggregation compatible with the stochasticity of the point process model. For all point pattern analyses, we used the grid-based software *Programita* [47].

Aggregation of *C. humilis* receiving dispersed seeds within the overall pattern of *C. humilis*

To assess if *C. humilis* containing (feces with) dispersed seeds were randomly distributed within the pattern of all *C. humilis* (with and without seeds) or they follow a particular spatial pattern (aggregation, dispersion), we also used qualitatively marked point pattern techniques and the random labelling null model to represent lack of any spatial structure. The same process and analysis were conducted as in the previous case but, in this occasion, the used mark was the

presence (1) or absence (0) of seed in each individual *C. humilis*. In parallel, these same analyses were carried out but excluding the *Rubus'* seeds since their great number could distort the spatial pattern for the presence of seeds on *C. humilis* (see Supporting Information, S2 Spatial analysis excluding *Rubus'* seeds). Since results were rather consistent whether we considered or not *Rubus*'s seeds, here we provide the results when all seed species are included.

Association of the number of dispersed seeds on *C. humilis*

To find out whether the number of dispersed seeds within bird feces deposited on *C. humilis* has a spatial association among different *C. humilis* we used quantitative marked point patterns techniques and the random labelling null model to represent lack of any spatial structure.

To quantify potential spatial associations in the number of seeds on different *C. humilis* we used the univariate mark correlation function as summary statistic [38, 43, 47, 48], ergo we wanted to find out if closer *C. humilis* had a higher number of seeds than isolated ones. In addition to its location, each *C. humilis* was characterized by a quantitative mark: (i.e. the number of dispersed seeds (of any species).

Mark correlation functions are based on pairs of study units (in this case *C. humilis* individuals) and the idea of estimating the mean value of a test function $t(m_i, m_j)$ of the two marks m_i and m_j, taken over all the ordered pairs *i-j* of *C. humilis* which have interpoint distances of $r \pm h$, being h the parameter bandwidth, which must be wide enough to produce a sufficient number of pairs in each distance class r but small enough to reveal significant biological detail [43, 49]. From all possible test functions $t(m_i, m_j)$ [43], we selected the following three:

(*i*) The r-mark correlation function $k_{m1.}(r)$ which is based on the test function: $t(m_i, m_j) = m_i$. $k_{m1.}(r) > 1$ indicates that the number of seeds on *C. humilis* that have neighbouring *C. humilis* at distance r is larger than the average of seeds, showing a positive effect of *C. humilis* aggregation on seed number. By contrast, if $k_{m1.}(r) < 1$, it would indicate a negative effect of aggregation and

a positive effect of isolation of *C. humilis* on seed number [49]. Finally, if $k_{m1.}$ (r) ~ 1, indicates that the number of seeds is not affected by the distance to *C. humilis* individuals.

(*ii*) Another mark correlation function was calculated to characterize the spatial covariance in number of seeds of two *C. humilis* separated by distance r. This test function is known as Schlather's: $t(r, m_i, m_j) = [m_i - \mu(r)][(m_j - \mu(r)]$ which results in a Moran's I like summary statistic $I_{mm}(r)$, a spatial variant of the Pearson correlation coefficient between the variables m_i and m_j defined by the ordered *i-j* pairs of *C. humilis* separated by distance $r \pm h$.

(*iii*) Lastly, we used a function that relates the number of seeds on a *C. humilis* directly to the density of neighbouring *C. humilis*, called "density correlation function" [49]: $C_{m,K}(r)$. This function estimates the Pearson correlation coefficient between the number of seeds m_i and the number of neighbours within distance r, based on the following equation: $t(r, m_i, K_i) = [m_i = \mu][(\lambda K_i(r) - \lambda K_i(r)]$. Where m_i is the number of seeds of the focal *C. humilis i*, λ is the overall density of *C. humilis* in the study area, μ is the mean number of seeds of the population, $\lambda K_i(r)$ is the number of neighbours around the focal *C. humilis i* within distance r for all *C. humilis*.

To test whether the data show spatial correlation, the observed values of the three functions mentioned above were contrasted to the data obtained from simulations of a null model without spatial correlation in the marks. The null model consisted in shuffling randomly the marks (number of seeds) over all *C. humilis*, conducting a total of 199 simulations. These simulations were also useful to estimate the simulation envelopes for the three functions, being the fifth highest and lowest values of the summary statistics among all simulations of the null mode [38, 49].

Effect of palm traits

To analyse how our response variables (presence of bird feces, presence of dispersed seeds and number of dispersed seeds) were influenced by the size (height, area and volume) and gender (male, female) of *C. humilis* individuals, as well as by the presence (and height: <25cm, 25-

100cm, >100cm) of beneficiary species, three sets of generalized linear models (GLMs) were fitted per study plot.

Model 1 assessed the effect of palm traits on the likelihood of presence of bird feces, Model 2 evaluated the probability of finding dispersed seeds depending on such characteristics and Model 3 appraised the number of seeds based on those characteristics. Both Model 1 and Model 2 have a binary response variable (presence = 1, absence = 0), and thus were fitted with a binomial distribution and a logit link. Finally, Model 3, was fitted with a Poisson distribution with logit link.

A set of competing models were fitted and the best candidate model was selected based on Akaike's Information Criterion (AIC), allowing to compare different hypothesis and know their supportive level on the data by weighting them (AICc; [50]). All analyses were performed in R 4.0.3 statistical software [51]. Additionally, these same models were carried out but using the interaction between *C. humilis* Height (m) and Area (m^2) instead of the variable Volume (m3). Results were rather consistent with those from the previous analysis (see Supporting Information, S3 Effect of palm traits).

Results

Overall patterns

We studied total of 109 *C. humilis* in Matasgordas' plot, from which were collected 705 bird feces and 28 seeds in 2019 and 206 bird feces and 16 seeds in 2020 (Fig 4). In Reserva's plot, we monitored 180 *C. humilis*, having been collected 1560 feces and 340 seeds in 2019, and 527 feces and 145 seeds in 2020. Nevertheless, not all dispersed seeds were contained within the feces since a small fraction was regurgitated by the birds. Six shrub species were identified among the collected seeds: *A. aphyllus*, *D. gnidium*, *O. europaea* var. *sylvestris*, *P. angustifolia*, *P. lentiscus* and *R. ulmifolius*. Finally, a large fraction of *C. humilis* acted as perch for birds and

contained their feces: 42.2% (2019) and 37.6% (2020) in Matasgordas and 43.9% (2019) and 27.8% (2020) in Reserva (Table 1).

Figure 4. **Distribution of *C. humilis* in Matasgordas (upper) and Reserva (lower) plots.** (A) *C. humilis* in Matasgordas with (black solid points) and without (white circles) bird feces or regurgitations. (B) *C. humilis* in Matasgordas with (black solid points) and without (white circles) dispersed seeds. (C) *C. humilis* in Reserva with (black solid points) and without (white circles) bird feces. (D) *C. humilis* in Reserva with (black solid points) and without (white circles) dispersed seeds.

Besides, some *C. humilis* had beneficiary woody plants growing within them. In Reserva and Matasgordas 104 out of 180 individuals (57.78%) and 71 out to 109 (65.14%) had beneficiary plants, respectively. A total of nine different beneficiary species were found, all of them present in Matasgordas but just five were recorded in Reserva (Table 1).

Spatial patterns

Aggregation of *C. humilis* used as perches within the overall pattern of *C. humilis*

Analysis of how *C. humilis* used as perches by birds (i.e. receiving bird feces) were distributed within the overall pattern of *C. humilis* showed the following results. In Matasgordas, the probability that two *C. humilis*, separated by a distance *r*, both used as perches (statistic $p_{11}(r)$), was contained within the expectation of random labelling (Fig 5A). The probability that two separated *C. humilis*, with just one comprising feces (statistic $p_{12}(r)$), was included within the simulation envelops generated by random labelling (Fig 5C). Finally, the observed density of neighbouring *C. humilis* (regardless of having feces or not; statistic $g_{i,i+j}(r) - g_{j,i+j}(r)$) around *C. humilis* with feces was within the simulation envelops generated by a random labelling mode (Fig

5E). Thus, in Matasgordas, the observed probabilities described by the three statistics were within the envelops generated by random labelling models, which indicates they are compatible with the stochasticity of the process model.

On the contrary, in Reserva, the probability that two *C. humilis* separated for distances 1-6m, both having feces ($p_{11}(r)$ statistic), was lower than the expectation under random labelling. The bivariate $p_{12}(r)$ showed that *C. humilis* with and without feces were further away than expected under random labelling up to scales of 6m (Fig 5D). Finally, the density of neighbouring *Chamaerops humilis* (having or not feces) around *C. humilis* with feces was significantly lower than expected by random labelling for distances up to scales of 7m (Fig 5F). Overall, these results indicate a tendency by frugivorous birds in Reserva towards using and defecating in isolated *C. humilis* individuals.

Figure 5. Analysis of *C. humilis* with feces in the two study plots (Matasgordas and Reserva) areas using mark connection functions as summary statistics. (A, B) The mark connection function $p_{11}(r)$ gives the conditional probability that, from two *C. humilis* that are separated by distance *r*, both are type 1 (i.e., with feces). (C, D) The mark connection function $p_{12}(r)$ gives the conditional probability that, from two *C. humilis* that are separated by distance *r*, the first is type 1 (i.e., with feces) and the second is type 2 (i.e., without feces). (E, F) The test statistic $g_{1,1+2}(r)$ - $g_{2,1+2}(r)$ compares the density of *C. humilis* (i.e., 1+2) around *C. humilis* with feces (i.e., type 1) with the density of *C. humilis* (i.e., 1+ 2) around *C. humilis* without feces (i.e., type 2). The expected mark connection function statistics (gray line) and the corresponding simulation envelopes (black lines), being the fifth lowest and highest values of the functions created by 199 simulations under random labelling, are also shown.

Distribution of *C. humilis* receiving dispersed seeds within the overall pattern of *C. humilis*

In Matasgordas, the probability $p_{11}(r)$, where two *C. humilis* separated by distance *r*, both having seeds, was contained within the envelopes of random labelling for all spatial scales (Fig

6A). The bivariate $p_{12}(r)$, the probability for two neighbouring *C. humilis*, one with seeds and other without them, was also contained between random labelling's envelopes (Fig 6C). Finally, the test statistic $g_{1,1+2}(r)$ - $g_{2,1+2}(r)$ shows that the density of neighbouring *C. humilis* around *C. humilis* receiving seeds did not differ from the mean *C. humilis* density. (Fig 6E).

Results in Reserva substantially differed from results from Matasgordas. The probability of two *C. humilis* separated by distance *r*, having both seeds ($p_{11}(r)$ statistic), was similar to expected of random labelling for all spatial scales (Fig 6B). However, the probability for two neighbouring *C. humilis*, just one of them with seeds ($p_2(r)$ statistic), was significantly lower (P = 0.01) that the obtained by using random labelling for distances up to 6m, between 12 and 29m and higher than 41m (Fig 6D). Neighbourhood density of *C. humilis* around *C. humilis* receiving seeds was significantly lower (P = 0.005) than the mean *C. humilis* density at scales of 1 to 6m (Fig 6F).

In short, the analysis of how *C. humilis* with seeds were distributed within the overall pattern of *C. humilis* showed that *C. humilis* with seeds were randomly distributed in Matagordas (Fig 6A, C, E) while, in Reserva, *C. humilis* with seeds were spatially isolated at small spatial scale (Fig 6D, F).

Figure 6. **Analysis of feces with seeds in the two study plots (Matasgordas and Reserva) using mark connection functions as summary statistics**. (A, B) The mark connection function $p_{11}(r)$ gives the conditional probability that, from two *C. humilis* that are separated by distance *r*, both are type 1 (i.e., with seeds). (C, D) The mark connection function $p_{12}(r)$ gives the conditional probability that, from two *C. humilis* that are separated by distance *r*, the first is type 1 (i.e., with seeds) and the second is type 2 (i.e., without seeds). (E, F) The test statistic $g_{1,1+2}(r)$ - $g_{2,1+2}(r)$ compares the density of *C. humilis* (i.e., 1+2) around *C. humilis* with seeds (i.e., type 1) with the density of *C. humilis* (i.e., 1+ 2) around *C. humilis* without seeds (i.e., type 2). The expected mark connection function statistics (gray line) and the

corresponding simulation envelopes (black lines), being the fifth lowest and highest values of the functions created by 199 simulations under random labelling, are also shown.

Distribution of the number of dispersed seeds across *C. humilis*

The observed r- mark correlation function for the number of seeds was included within the simulation envelop for all scales, indicating no relationship between distance among individual *C. humilis* and the number of dispersed seeds neither in Matasgordas (*P* = 0.95, Fig 7A) nor in Reserva (*P* = 0.31, Fig 7B). Similarly, the Schlather's correlation function did not show a correlation between number of seeds and distance among individuals at any spatial scales, neither in Matasgordas (*P* = 0.22, Fig 7C) nor in Reserva (*P* = 0.22, Fig 7D). Finally, the density correlation function did not indicate correlation between the number of seeds and the density of *C. humilis* at any scale neither in Matasgordas (*P* = 0.35, Fig 7E) nor in Reserva (*P* = 0.35, Fig 7F).

Figure 7. **Mark correlation functions to evaluate a potential spatial structure in the number of dispersed seeds found on *C. humilis* in Matasgordas and Reserva study plots.** (A, B) The r- mark correlation function describes the mean number of seeds (m_i) on a *C. humilis* at distance r of another *C. humilis*. (C, D) Schlather's correlation function quantifies the correlation between the number of seeds on two different *C. humilis* separated by distance r. (E, F) Density correlation function assesses the correlation between the number of seeds and the number of neighbours located at a distance r. The expected mark connection function statistics (gray line) and the corresponding simulation envelopes (black lines), being the fifth lowest and highest values of the functions created by 199 simulations under random labelling, are also shown.

Effect of palm traits

We found that *C. humilis* in Matasgordas were on average (8.80 m^2) smaller than in Reserva (15.26 m^2). Also, the richness of beneficiary species beneath *C. humilis* was slightly lower in Matasgordas than in Reserva, with 0.89 and 1.07 beneficiary species per individual, respectively.

For Matasgordas, the best model (AIC = 133.97) showed that presence of bird feces on *C. humilis* were positively related to their height and area, but negatively to volume, although none of these variables were found significant (Table 2. Model 1). The best model for Reserva (AIC = 196.63) indicated that presence of bird feces on *C. humilis* was higher in male than females and positively influenced by palm area, but negatively to the richness of beneficiary species contained within them. Nevertheless, only the palm area was found significant ($P < 0.001$).

Table 2. GLM carried out to analyze the palm traits Matasgordas' plot.

Explanatory variable	Competing models		β	SE	AICc	ΔAIC	Weighted AIC
Model 1							
Presence of Bird Feces	Height (m) + Area (m^2) + Volume (m^3)				133.97	0	0.62
		Intercept*	-4.5863	2.2444			
		Height (m)	5.9296	3.5128			
		Area (m^2)	0.4108	0.2187			
		Volume (m^3)	-0.4497	0.2822			
	Gender + Height (m) + Area (m^2) + Volume (m^3)				135.67	1.7	0.27
	Gender + Height (m) + Area (m^2) + Volume (m^3) + Richness				137.54	3.57	0.10
	Gender + Height (m) + Area (m^2) + Volume (m^3) + Richness + Beneficiary height (cm)				143.11	9.14	0.01
Deviance = 90.343							
Model 2							
Presence of Dispersed Seeds	Gender + Height (m)				96.343	0	0.05
		Intercept*	-4.9694	1.4405			
		Gender (Male)	0.9151	0.557			
		Height (m)*	4.2936	1.8368			
	Gender + Height (m) + Volume (m^3)				98.21	1.87	0.02
	Gender + Height (m) + Area (m^2) + Volume (m^3)				99.03	2.69	0.01
	Gender + Height (m) + Area (m^2) + Volume (m^3) + Richness				100.73	4.39	< 0.01
	Gender + Height (m) + Area (m^2) + Volume (m^3) + Richness + Beneficiary height (cm)				107.56	11.2	< 0.01
Deviance = 126.52							
Model 3							
Number of Dispersed Seeds	Gender + Height (m) + Volume (m^3)				191.41	0	0.11
		Intercept*	-5.9884	1.15268			
		Gender(Male)*	0.82204	0.35402			
		Height (m)*	7.7374	2.03287			
		Volume (m^3)*	-0.1341	0.06466			
	Gender + Height (m) + Area (m^2) + Volume (m^3)				192.77	1.36	0.06
	Gender + Height (m) + Area (m^2) + Volume (m^3) + Richness				194.47	3.06	0.02

					AICc	ΔAIC	Weighted AIC
	Gender + Height (m) + Area (m^2) + Volume (m^3) + Richness + Beneficiary height (cm)				196.18	4.77	0.01
Deviance = 133.75							

Summary of fitted parameters and models employed to analyse the presence of bird feces (model 1), the presence of dispersed seeds (model 2) and the number of dispersed seeds (model 3) on *C. humilis* in Matasgordas study plot. Competitive models are ranked from the lowest AICc value (best model) to the highest one, being significant variables indicated with asterisks (*).

Presence of dispersed seeds in Matasgordas depended on *C. humilis* gender and height (Table 2. Model 2), according to the best model obtained (AIC = 96.343). Nonetheless, only the height was significant ($P < 0.05$) with larger *C. humilis* individuals having a higher number of dispersed seeds. In Reserva, the best model obtained (AIC = 137.98) showed that height and richness of beneficiary species had a positive effect on the number of dispersed seeds, although just the last one was significant in this case ($P < 0.01$) (Table 3. Model 2).

Table 3. GLM carried out to analyze the palm traits Reserva's plot.

**Explanatory variable**	**Competing models**		**β**	**SE**	**AICc**	**ΔAIC**	**Weighted AIC**
Model 1							
Presence of Bird Feces	Gender + Area (m^2) + Richness				196.63	0	0.54
		Intercept*	-0.7758	0.30364			
		Gender (Male)	0.55831	0.35223			
		Area (m^2)*	0.0891	0.02394			
		Richness	-0.4846	0.26617			
	Gender + Area (m^2) + Volume (m^3) + Richness				197.88	1.25	0.29
	Gender + Height (m) + Area (m^2) + Volume (m^3) + Richness				199.49	2.86	0.13
	Gender + Height (m) + Area (m^2) + Volume (m^3) + Richness + Beneficiary height (cm)				201.76	5.13	0.04
Deviance = 188.63							
Model 2							
Presence of Dispersed Seeds	Height (m) + Richness				137.98	0	0.62
		Intercept*	-3.3834	0.5486			
		Height (m)	0.4797	0.2728			
		Richness*	1.9713	0.7313			
	Height (m) + Volume (m^3) + Richness				139.86	1.88	0.24
	Height (m) + Area (m^2) + Volume (m^3) + Richness				141.64	3.66	0.10
	Gender + Height (m) + Area (m^2) + Volume (m^3) + Richness				143.5	5.52	0.04
	Gender + Height (m) + Area (m^2) + Volume (m^3) + Richness + Beneficiary height (cm)				147.44	9.46	< 0.01
Deviance = 131.98							
Model 3							
Number of Dispersed Seeds	Gender + Height (m) + Area (m^2) + Volume (m^3) + Richness + Beneficiary height (cm)				1273.09	0	1
		Intercept*	-2.8228	1.07845			
		Gender (Male)*	1.80173	0.12685			
		Height (m)*	-1.9455	0.45779			
		Area (m^2)*	-0.1135	0.01363			
		Volume (m^3)*	0.13377	0.01437			
		Richness*	0.95673	0.0926			

	Beneficiary height (25-100cm)	0.7794	1.03393
	Beneficiary height (>100cm)*	2.59102	1.0217
Deviance = 1155.7			

Summary of fitted parameters and models employed to analyse the presence of bird feces (model 1), the presence of dispersed seeds (model 2) and the number of dispersed seeds (model 3) on *C. humilis* in Reserva study plot. Competitive models are ranked from the lowest AICc value (best model) to the highest one, being significant variables indicated with asterisks (*).

According to the best model in Matasgordas (AIC = 191.41), male *C. humilis* had a higher number of dispersed seeds than females ($P < 0.05$) and height had a positive and significant ($P < 0.001$) influence on the number of seeds found on *C. humilis*. Palm volume had a negative effect on the number of seeds ($P < 0.05$) (Table 2. Model 3). Finally, in Reserva, just one model was fitted (AIC = 1273.09). This model predicted: (*i*) a negative relationship between height of *C. humilis* and the number of dispersed seeds ($P < 0.0001$); (*ii*) a higher number of dispersed seeds in male *C. humilis* than in females ($P < 0.0001$); (*iii*) a positive relationship between *C. humilis* size and the number of dispersed seeds ($P < 0.0001$ for both area and volume); (*iv*) a positive relationship between the richness of beneficiary species in *C. humilis* and the number of dispersed seeds ($P < 0.0001$); and (*v*) a higher number of dispersed seeds in *C. humilis* holding tall (> 100 cm in height) beneficiary woody individuals as compared to both small (< 25 cm) and intermediate (25-100 cm) beneficiary individuals ($P < 0.05$; Table 3, Model 3).

Discussion

The role of pioneer plants acting as perches for seed dispersers is crucial for the restoration of many human-disturbed habitats by increasing seed arrival and eventually seedling establishment [52, 53, 54]. Little is known concerning how the spatial distribution of perches influences the spatial patterns of seed dispersal by frugivorous birds, and the subsequent patterns of establishment of woody species [55]. Using a spatially explicit approach, we detected a random spatial structure in both the number of visits (bird feces) and the dispersed seeds found on *C. humilis* in one study plot (Matasgordas) while an aggregated pattern in the other one (Reserva).

Frugivorous birds tended to disperse seeds into a small number of *C. humilis* individuals (usually male and large-sized ones) that acted as “hotspots” of seed arrival. Our study also revealed that the species of dispersed seeds partially matched the woody beneficiary species growing inside *C. humilis*, reinforcing the idea that this palm species plays a key role in the restoration of disturbed habitats.

Widespread perching effect by *C. humilis*

Considering that our sampling period did not take place throughout the year and that an unknown fraction of the bird feces presents in target *C. humilis* individuals surely went unnoticed, the data obtained could be considered as a hint of how widespread the "perching” effect in both study plots. Therefore, the high presence of (frugivorous) bird feces and dispersed seeds in both study plots is remarkable.

Besides, it is apparent that some seeds can emerge seedling beneath *C. humilis*, since all seeds collected belonged to beneficiary woody species found within *C. humilis*. Nevertheless, we did not find seeds of the beneficiary species *C. salviifolius*, *P. bourgaeana*, *Q. suber* and *S. genistoides*, which are usually associated to *C. humilis*. Most of these plant species are seldom dispersed by birds. Other animals are more likely to disperse them, such as insects for *C. salviifolius* or *S. genistoides* seeds [56, 57, 58] or mammals, such as ungulates (red deer, wild boars, domestic cattle) and carnivores (Eurasias badger, red fox) for *P. bourgaeana* seeds [59]. Therefore, the presence of these woody species associated to *C. humilis* reveals that it does not attract just bird-dispersed plants but also mammal-dispersed plants, accentuating its role in habitat restoration.

Spatial patterns of the perching effect

Our study has revealed different spatial patterns in both study plots at the three target organizational levels studied: (*i*) *C. humilis* receiving bird feces, (*ii*) *C. humilis* receiving (feces with) dispersed seeds, and (*iii*) number of dispersed seeds arriving to *C. humilis*.

No spatial pattern was found in Matasgordas' plot, where the distribution of *C. humilis* did not seem to play a decisive role in attracting frugivorous birds. By contrast, in Reserva's plot, where isolated palms were more frequent than in Matasgordas, there was strong evidence that birds preferred isolated *C. humilis*, at least for those separated from conspecifics by a distance up to 7 m. Aggregated perches could be found attractive for seed dispersers and, therefore, more visited by them [60], since they would act as shelters against predators [59]. This premise has been used for designing ecological restoration and conservation actions in mixed ecosystems, where trees or other woody plants, acting as perches and nurses, are planted following aggregated spatial patterns [62, 63, 64, 65]. However, by concentrating frugivorous activity to aggregated planted trees, seed dispersal could be limited [16, 55]. Therefore, our results supporting the role of isolated perches diverge from the most widespread tendency in ecological restoration of aggregating trees and shrubs during revegetation efforts [65].

Spatial patterns of perching effect differed between study plots. On the one hand, these differences could be attributed to the changing distance that frugivorous birds must fly between the main seed source area (Mediterranean scrubland) and our study plots where target *C. humilis* individuals are located. This distance is longer in Matasgordas (up to 350m) than in Reserva (150-200m). Besides, it seems reasonable to expect that birds select *C. humilis* located on the edge of the plot, since these individuals are closer to the Mediterranean scrubland and thus allow birds to move between habitats with a minimal consumption of energy. On the other hand, both plots are open habitats and have been intensively disturbed by humans in the past, but with some decades apart [16, 41]. Thus, study plots are in different stages of ecological succession and the vegetation surrounding them is different too. For instance, in Matasgordas' plot there are more scattered trees (mostly *Q. suber* and *O. europaea* var. *sylvestris*) than in Reserva's plot. The reduced number of

feces and seeds and the lack of spatial patterns found in Matasgordas could relate to the bird's intensive usage of such scattered trees. A recent study has shown that in places where ecological succession is more advanced (Reserva) the "nursing" effect of *C. humilis* may be diluted among different shrub species [4], since the landscape is dominated by shrub-encroached communities. The "perching" effect of *C. humilis*, however, seems to follow the opposite trend, being enhanced in the most shrub-encroached landscape (i.e. Reserva) probably because of the few available trees for perching.

Interestingly, we found that most of dispersed seeds were concentrated in a small number of *C. humilis* individuals that acted as "hotspots" of seed arrival. For example, in Reserva there was a single large *C. humilis* individual receiving 47.4% (N = 485) of the total seeds found in that plot. Such a strong concentration of dispersed seeds within a few *C. humilis* individuals likely explains that fact that we did not find further spatial pattern of the number of dispersed seeds. Features (other than spatial patterns) that make particular *C. humilis* individuals hotspots of seed arrival are discussed below.

Palm trait's influence on perching effect

This investigation identified some features that made particular *C. humilis* attractive "perching" sites for frugivorous birds and, therefore, hotspots for seed arrival. However, this pattern was found only in Reserva's plot, and not in Matasgordas' plot. In Reserva, larger *C. humilis* individuals received more seed as compared to small ones. The greater surface of large individuals could attract more birds since it implies a greater number of entry points to *C. humilis* interior, where the bird could be safe from predators and adverse weatherly conditions (e.g. high temperatures, rainfall).

The use of *C. humilis* as "perching" site by birds does not necessarily implies seed arrival since not all recorded birds are seed dispersers [66]. However, we sampled during the seed dispersal season, time when some birds change their diet into a more fruit-based one. For instance,

it has been reported that *P. ochruros*, *S. atricapilla* and *S. malenocephala* are able to quickly change their diet when fleshy fruits become available, while other species such as *S. rubicola* show a more gradual change to a frugivorous diet [67, 68]. Also, *S. unicolor*, *P. ochruros*, *P. collybita*, *P. pica*, and *S. rubicola* has been reported as fruit-eater, although in lesser quantities [69, 70, 71, 72, 73]. Therefore, although not all recorded bird species contribute equally to seed dispersal, fleshy fruits represent a considerable fraction of most of their diets during the seed dispersal season.

Therefore, although not all recorded bird species contribute equally to seed dispersal, fleshy fruits represent a considerable fraction of their diet during this the seed dispersal season.

Secondly, the preference of taller *C. humilis* by frugivorous birds could relate to their own requirements as well as to characteristics of the surrounding landscape. Taller *C. humilis* could suppose safer places since they would provide greater visual fields to birds to spot potential predators. Also, taller perches are likely better sites to take flight towards other habitats (e.g. the Mediterranean scrubland) adjacent to the studied site, which may be more suitable habitats for them [64, 75, 76].

The detected positive effect of richness of beneficiary species within *C. humilis* in Reserva could be related to some of them producing fleshy fruits (e.g. *Rubus ulmifolius*, *Asparagus aphyllus*), acting thus as an additional attractant for frugivorous birds [77, 78, 79]. Richness of beneficiaries was also found positively related to the number of seeds found on *C. humilis*, which is likely a consequence of the increased number of frugivorous bird visits. Additionally, beyond the fact of bearing fleshy fruits, the own beneficiary species could be used as a perch and thus attract birds. Therefore, a similar reasoning explained earlier about how *C. humilis* height influences seed dispersal, could be applied for the revealed positive effect of height of beneficiary plants. Finally, it seemed that male *C. humilis* were more likely to receive dispersed seeds than females. We speculate that male *C. humilis* could be selected by birds over females because the former have a lower associated predation risk. Females *C. humilis* produce fruits that are too big for birds, but they are often eaten by medium-sized carnivores such as the Eurasian badger and

the red fox [33]. Although these mammals are mainly nocturnal, their presence likely prevents birds from using female *C. humilis* in fruit as shelter at night, and thus may dissuade them at day too. The use of *C. humilis* in ecological restoration should follow a balance between male and female plants. Male plants would attract preferably birds that would disperse seeds of woody species present in the surrounding area, while female plants would attract medium-sized carnivores and ungulates that would mostly disperse seed of species such as *P. bourgaena* and *C. humilis*.

In summary, taller (and isolated) male *C. humilis* with a higher diversity of beneficiary species seemed to be the most suitable for attracting frugivorous birds and, therefore, received higher number of dispersed seeds. However, this pattern was not entirely confirmed in Matasgordas' plot, likely because of a much smaller sample sizes (i.e. lesser numbers of both *C. humilis* used as perches and of dispersed seeds).

Implications for ecological restoration

In the last 30 years, restoration ecology has undergone considerable development thanks to the emerging new tools and techniques that facilitate the retrieval of biological properties of disturbed habitats [80]. Our study plots are a good representation of Mediterranean old fields, which have been targets for habitat restoration [5, 16]. Previous studies have demonstrated the value of *C. humilis* in these programs since it facilitates recruitment of numerous woody species dispersed by frugivores [4, 36]. Here, we have demonstrated that *C. humilis* also exerts a marked "perching" effect, crucial in restoration and ecological succession [27, 52, 81, 82]. The combination of artificial and natural perches with nurse plants could increase the seed arrival and recruitment leading to nucleation processes around them [4, 19, 20]. In this sense, to our knowledge, this study represents the first spatially explicit assessment of the relationship between natural perches and seed arrival via frugivorous birds in Mediterranean human-disturbed

landscapes. Our results could be used by managers to decide, for example, whether particular areas are adequate to promote seed arrival using natural or artificial perches.

Finding that dispersed seeds are concentrated in a few *C. humilis* individuals, which tend to be isolated from the rest, breaks surprisingly with the general trend of revegetating under an aggregation design (e.g. [64, 65]. Thus, ideas that were taken for granted in the past have changed thanks to further study and understanding (see [16]). For instance, traditionally, most of shrubs were considered as potential competitors for tree species in habitat restoration [83] and, therefore, as retarders of ecological succession, meaning that they were frequently removed from the area before the revegetation started [6]. This idea has changed during the last few decades and now it is widely known that many shrub species, under different circumstances, can act as nurses for late-successional species, remarking their important role in ecological restoration [28, 62, 84, 85, 86]. Furthermore, the pre-existing vegetation does not act only on flora, but also on the environment conditions (e.g. by reducing soil erosion) making restoration programs more affordable from an economic perspective [23, 63]. Thus, the strategy of using aggregated perches for increasing seed arrival and habitat restoration should not be advocated under all ecological circumstances.

Final conclusions

This investigation supports previous studies about the importance of the pioneer *C. humilis* for the restoration of human-disturbed Mediterranean landscapes. We prove for the first time its key role as natural perch, an ecological function that, though it is shared with some fleshy-fruited woody plants, is especially relevant in early stages of ecological succession when few pioneer species occur. Birds tended to disperse seeds into a small number of *C. humilis* individuals (usually male and large-sized ones) that acted as "hotspots" of seed arrival. Thanks to the double role of *C. humilis*, which attracts frugivorous birds that deliver considerable numbers of seeds of late-successional woody species and provides improved conditions for seedling emergence and

survival, it is likely to become a successful tool for ecological restoration in many habitats of the western Mediterranean basin.

References

1. Asner GP, Elmore AJ, Olander LP, Martin RE, Harris AT. Grazing systems, ecosystem responses, and global change. Annual Review of Environment and Resources 2004;29:261-99.
2. Foley JA, DeFries R, Asner GP, Barford C, Bonan G, Carpenter SR, et al. Global consequences of land use. Science 2005;309:570-574.
3. FAO (Food and Agriculture Organization of the United Nations) [Internet]. FAOSTAT; 2021 [updated 2021 June 29; cited 2021 June 29] Available from: http://faostat.fao.org/site/377/default.aspx
4. Garrote PJ, Castilla AR, Fedriani, JM. The endemic Mediterranean dwarf palm boosts the recolonization of old fields: Implications for restoration. Journal of Environmental Management 2019;250:109478.
5. Rey-Benayas JM, Martins A, Nicolau JM, Schulz JJ. Abandonment of agricultural land: an overview of drivers and consequences. Perspectives in Agriculture, Veterinary Science, Nutrition and Natural Resources 2007;2(57):1-14.
6. Rey PJ, Siles G, Alcántara JM. Community-level restoration profiles in Mediterranean vegetation: nurse-based vs. traditional reforestation. Journal of Applied Ecology 2009;46:937-945.
7. Brunet J. Plant colonization in heterogeneous landscapes: an 80-year perspective on restoration of broadleaved forest vegetation. Journal of Applied Ecology 2007;44:563-572.
8. Cramer VA, Hobbs RJ, Standish RJ. What's new about old fields? Land abandonment and ecosystem assembly. Trends in Ecology & Evolution 2008;23:104-112.
9. Bullock JM, Moy IL, Pywell RF, Coulson SJ, Nolan AM, Caswell H. Plant dispersal and colonization processes at local and landscape scales. In: Bullock JM, Kenward RE, Hails RS, editors. Dispersal Ecology. Oxford, UK: Blackwell Science; 2002. p. 279-302.
10. Rey-Benayas, J. M. Growth and mortality in Quercus ilex L. seedlings after irrigation and artificial shading in Mediterranean set-aside agricultural lands. Annals of Forest Science 1998;55:801-807.
11. Rey-Benayas JM, Navarro J, Espigares T, Zavala MA, Nicolau JM. Effects of artificial shading and weed mowing in reforestation of Mediterranean abandoned cropland with contrasting Quercus species. Forest Ecology and Management 2005;212:302-314.
12. Mouillot F, Ratte JP, Joffre R, Moreno JM, Rambal S. Some determinants of the spatio-temporal fire cycle in a Mediterranean landscape (Corsica, France). Landscape Ecology 2003;18:665-674.
13. Lindskog P, Mando A. (1992) *The relationship between research institutes and NGOs in the field of soil and water conservation in Burkina Faso.* Drylands Issue Paper Nº 39. International Institute for Environment and Development (IIED) London, UK.

14. Vogel, H. Soil erosion interrace farming: destruction of farming land induced by changes in land use in the Haraz mountains of Yemen. Eschborn, Germany. Deutsche Gesellschaft für Technische Zusammenarbeit (GIZ); 1992. 190 p.

15. Levine JM, Murrell DJ. The Community-Level Consequences of Seed Dispersal Patterns. Annual Review of Ecology, Evolution, and Systematics 2003;34(1):549-574.

16. Fedriani JM, Wiegand T, Ayllón D, Palomares F, Suárez-Esteban A, Grimm V. Assisting seed dispersers to restore oldfields: An individual-based model of the interactions among badgers, foxes and Iberian pear trees. Journal of Applied Ecology 2018;55(2):600-611.

17. Debussche M, Escarré J, Lepart J. Ornithochory and plant succession in Mediterranean abandoned orchards. Vegetatio 1982;48:255-266.

18. Howe HF, Smallwood J. Ecology of Seed Dispersal. Annual Review of Ecology and Systematics 1982;13(1):201-228.

19. Pausas JG, Bonet A, Maestre FT, Climent A. The role of the perch effect on the nucleation process in Mediterranean semi-arid oldfields. Acta Oecologica 2006;29(3):346-352.

20. Verdú M, García-Fayós P. Nucleation processes in Mediterranean bird-dispersed plant. Functional Ecology 1996;10:275-280.

21. Izhaki I, Walton PB, Safriel UN. Seed shadows generated by frugivorous birds in an Eastern Mediterranean scrub. Journal of Ecology 1991;79:575-590.

22. Herrera CM, Jordano P, López-Soria L, Amat JA. Recruitment of a mast-fruiting, bird-dispersed tree: bridging frugivore activity and seedling establishment. Ecological Monographs 1994;64:314-344.

23. Castro J, Zamora R, Hódar JA, Gómez JM, Gómez-Aparicio L. Benefits of Using Shrubs as Nurse Plants for Reforestation in Mediterranean Mountains: A 4-Year Study. Restoration Ecology 2004;12(3):352-358.

24. Connell JH, Slatyer RO. Mechanisms of Succession in Natural Communities and Their Role in Community Stability and Organization. The American Naturalist 1977;111(982):1119-1144.

25. Cruz-Alonso V, Villar- Salvador P, Ruiz- Benito P, Ibáñez I, Rey- Benayas JM. Long-term dynamics of shrub facilitation shape the mixing of evergreen and deciduous oaks in Mediterranean abandoned fields. Journal of Ecology 2019;108(3):1125-1137.

26. Debussche M, Lepart J. Establishment of woody plants in mediterranean old fields: opportunity in space and time. Landscape Ecology 1992;6:133-145.

27. Lamb P, Erskine P, Parrota J. Restoration of Degraded Tropical Forest Landscapes. Science 2005;310:1628-1632.

28. Garrote PJ, Castilla AR, Fedriani JM. Assessing the relative importance of nurse species on Mediterranean human- altered areas. Restoration Ecology 2021;29(4):e13402.

29. Wiegand T, Moloney KA. *Handbook of spatial point-pattern analysis in ecology*. Boca Raton, FL, USA: Chapman & Hall/CRDC applied environmental statistics. CRC Press/Taylor & Francis; 2014. 538 p.

30. López-González GA. Los árboles y arbustos de la Península Ibérica e Islas Baleares. 2nd ed. Madrid, Spain: Ediciones Mundi-Prensa; 2006. 1731 p.

31. Guzmán B, JM Fedriani, Delibes M, Vargas P. The colonization history of the Mediterranean dwarf palm (*Chamaerops humilis* L., Palmae). Tree Genetics and Genomics 2017;13:1-10.

32. Charco J, Becerra M, Santa-Barbara C, Fernández C, García FJ, Triano E, Vizoso MT, Baena L. Árboles y arbustos autóctonos de Andalucía. Valencia, Spain: Centro de Investigaciones Ambientales del Mediterráneo; 2014. 593 p.

33. Fedriani JM, Ferreras P, Delibes M. Dietary response of the Eurasian badger, Meles meles, to a decline of its main prey in the Doñana National Park. Journal of Zoology 1998;245:214-218.

34. Castañeda I, Fedriani JM, Delibes M. Red deer (Cervus elaphus) disperse viable seeds spitting from the cud. Mammalian Biology 2018;90:89-91.

35. Selwyn M, Garrote PJ, Castilla AR, Fedriani JM. Interspecific interactions among functionally diverse frugivores and their outcomes for plant reproduction: A new approach based on camera-trap data and tailored null models. PloS one 2020;15(10):e0240614.

36. García-Novo F, Escudero-García JC, Carotenuto L, García-Sevilla D, Fernández-Lo Faso RP. The restoration of El Partido stream watershed (Doñana Natural Park): A multiscale, interdisciplinary approach. Ecological Engineering 2007;30(2):122-130.

37. Soliveres L, DeSoto L, Maestre FT, Olano JM. Spatio-temporal heterogeneity in abiotic factors modulate multiple ontogenetic shifts between competition and facilitation. Perspectives in Plant Ecology 2010;12(3):227-234.

38. Fedriani JM, Wiegand T. Hierarchical mechanisms of spatially contagious seed dispersal in complex seed-disperser networks. Ecology 2014;96(2):514-526.

39. Allier C, González-Bernáldez F, Ramírez-Díaz L. Mapa ecológico de la Reserva Biológica de Doñana. Sevilla. Spain: Consejo Superior de Investigaciones Científicas; 1974. 7 p.

40. Martín R, Rodríguez A, Delibes M. Local feeding specialization by badgers (Meles meles) in a mediterranean environment. Oecologia 1995;101:45-50.

41. Granados-Corona M, Martin-Vicente A, García Novo F. Long-term vegetation changes on the stabilized dunes of Doñana National Park (SW Spain). Vegetatio 1998;75(1-2): 73–80.

42. Garrote PJ, A Castilla, JM Fedriani. Coping with changing plant-plant interactions in restoration ecology: effect of species, site, and individual variation. Applied Vegetation Sciences. 2022;25(1): e12644.

43. Illian J, Penttinen A, Stoyan H, Stoyan D. Statistical analysis and modelling of spatial point patterns. Hoboken, NJ, USA: John Wiley & Sons; 2008. 560 p.

44. Jacquemyn H, Endels P, Honnay O, Wiegand T. Spatio-temporal analysis of seedling recruitment, mortality and persistence into later life stages in the rare Primula vulgaris. Journal of Applied Ecology 2010;47: 431-440.

45. Raventós J, Wiegand T, De Luis M. Evidence for the spatial segregation hypothesis: a test with nine-year survivorship data in a Mediterranean fire-prone shrubland show that interspecific and density-dependent spatial interactions dominate. Ecology 2010;91:2110-2120.

46. Stoyan D, Stoyan H. Fractals, random shapes and point fields: methods of geometrical statistics (Vol. 302). Hoboken, NJ, USA: Wiley-Blackwell; 1994. 408p.

47. Wiegand T, Moloney KA. Handbook of spatial point-pattern analysis in ecology. New York, NY, USA: Chapman and Hall/CRC. 2014. 540p.

48. Getzin S, Worbes M, Wiegand T, Wiegand K. Size dominance regulates tree spacing more than competition within height classes in tropical Cameroon. Journal of Tropical Ecology 2011;27:93-102.

49. Fedriani JM, Wiegand T, Calvo G, Suárez-Esteban A, Jácome M, Żywiec M, et al. Unravelling conflicting density- and distance-dependent effects on plant reproduction using a spatially explicit approach. Journal of Ecology 2015;103:1344-1353.

50. Burnham KP, Anderson DR. Model selection and multimodel inference: a practical information-theoretic approach. 2nd ed. New York, USA: *Springer-Verlag*; 2002. 488 p.

51. R Core Team. R: A language and environment for statistical computing. Vienna, Austria: R Foundation for Statistical Computing, 2021. Available from: https://www.R-project.org/.

52. Graham LLB, Page SE. Artificial bird perches for the regeneration of degraded tropical peat swamp forest: a restoration tool with limited potential. Restoration Ecology 2012;20:631-637.

53. La Mantia T, Rühl J, Massa B, Pipitone S, Lo Verde G, Bueno RS. Vertebrate-mediated seed rain and artificial perches contribute to overcome seed dispersal limitation in a Mediterranean old field. Restoration Ecology 2019;27(6):1393-1400.

54. Zanini L, Ganade G. Restoration of Araucaria Forest: The Role of Perches, Pioneer Vegetation, and Soil Fertility. Restoration Ecology 2005;13(3):507-514

55. Pegman APM, Perry GLW, Clout MN. Exploring the interaction of avian frugivory and plant spatial heterogeneity and its effect on seed dispersal kernels using a simulation model. Ecography 2017;40:1098-1109.

56. Bastida F, Talavera S. (2002) Temporal and Spatial Patterns of Seed Dispersal in Two Cistus Species (Cistaceae). Annals of Botany 2002;89(4):427-434.

57. Talavera S, Gibbs PE, Herrera J. Reproductive biology of Cistus ladanifer (Cistaceae). Plant Systematics and Evolution 1993;186:123-134.

58. Melendo M, Giménez E, Cano E, Gómez-Mercado F, Valle F. The endemic flora in the south of the Iberian Peninsula: taxonomic composition, biological spectrum, pollination, reproductive mode and dispersal. Flora. Morphology, Distribution, Functional Ecology of Plants 2003;198(4):260-276.

59. Fedriani JM, Delibes M. Seed dispersal in the Iberian pear, Pyrus bourgaeana: A role for infrequent mutualists. Écoscience 2009;16(3):311-321.

60. Carlo TA, Morales JM. Inequalities in fruit-removal and seed dispersal: consequences of bird behavior, neighborhood density and landscape aggregation. Journal of Ecology 2008;96(4):609-618.

61. Howe HF. Fear and frugivory. American Naturalist, 1979;114:925-931.

62. Callaway RM, Pugnaire FI. Facilitation in plant communities. In Pugnaire FI, Valladares F, editors. *Handbook of functional plant ecology*. New York, USA: Marcel Dekker; 1999. p. 623-648.

63. Gómez-Aparicio L, Zamora R, Gómez JM, Hódar JA, Castro J, Baraza E. Applying plant facilitation to forest restoration: a meta-analysis of the use of shrubs as nurse plants. Ecological Applications 2004;14(4):1128-1138.

64. Pueyo Y, Moret-Fernández D, Saiz H, Bueno CG, Alados CL. Relationships Between Plant Spatial Patterns, Water Infiltration Capacity, and Plant Community Composition in Semi-arid Mediterranean Ecosystems Along Stress Gradients. Ecosystems 2013;16:452-466.

65. Rey-Benayas JM, Bullock JM, Newton AC. Creating woodland islets to reconcile ecological restoration, conservation, and agricultural land use. Frontiers in Ecology and the Environment 2008;6:329-336.

66. Schupp EW, Jordano P, Gómez JM. Seed dispersal effectiveness revisited: a conceptual review. New Phytologist 2010;188(2):333-353.

67. Campo-Celada M, Jordano P, Benítez-López A, Gutiérrez-Expósito C, Rabadán-González J, Mendoza I. Assessing short and long-term variations in diversity, timing and body condition of frugivorous birds. Oikos 2022;2:e08387

68. Carnicer J, Jordano P, Melían CJ. The temporal dynamics of resource use by frugivorous birds: a network approach. Ecology 2009;90(7):1958-1970.

69. Jordano P. Avian Fruit Removal: Effects of Fruit Variation, Crop Size, and Insect Damage. Ecology, 1987:68(6):1711-1723.

70. Carnicer J, Jordano P, Melían CJ. The temporal dynamics of resource use by frugivorous birds: a network approach. Ecology, 2009;90(7):1958-1970.

71. Gonzalez-Varo JP, Carvalho CS, Arroyo JM, Jordano P. Unravelling seed dispersal through fragmented landscapes: Frugivore species operate unevenly as mobile links. Molecular Ecology, 2017;26(16):4309-4321.

72. Green AJ, Elmberg J, Lovas-Kiss A. Beyond Scatter-Hoarding and Frugivory: European Corvids as Overlooked Vectors for a Broad Range of Plants. Front. Ecol. Evol., 2019;7(133).

73. Campo-Celada M, Jordano P, Benítez-López A, Gutiérrez-Expósito C, Rabadán-González J, Mendoza I. Assessing short and long-term variations in diversity, timing and body condition of frugivorous birds. Oikos, 2022;2:e08387

74. Vergara PM. Matrix-dependent corridor effectiveness and the abundance of forest birds in fragmented landscapes. Landscape Ecology, 2011;26:1085

75. Askins RA, Folsom-O'Keefe CM, Hardy MC. Effects of Vegetation, Corridor Width and Regional Land Use on Early Successional Birds on Powerline Corridors. PLoS ONE 2012;7(2):e31520.

76. Piratelli AJ, Piña-Rodrigues FCM, Raedig C. Integrating Biodiversity Conservation into Agroecosystem Management: Using Birds to Bring Conservation and Agricultural Production Together. In: Nehren U, Schlüter S, Raedig C, Sattler D, Hissa H, editors. *Strategies and Tools for a Sustainable Rural Rio de Janeiro*. Cham, Switzerland: Springer; 2019. P. 139-153.

77. Rey PJ. Preserving frugivorous birds in agro-ecosystems: lessons from Spanish olive orchards. Journal of Applied Ecology 2011;48:228-237.

78. Kaur N, Dhanju CK. Food and Feeding Habits of Common Birds of Agroecosystems. Indian Journal of Ecology 2013;40(1):83-86.

79. Morgado R, Pedroso R, Porto M, Herrera JM, Rego F, Moreira F, Beja P. Preserving wintering frugivorous birds in agro-ecosystems under land use change: Lessons from intensive and super-intensive olive orchards. Journal of Applied Ecology 2021;58(12):2975-2986.

80. Van Andel J, Aronson J. Restoration Ecology: The New Frontier. Oxford, UK: Blackwell Scientific Publications; 2006. 400 p.

81. Martínez-López V, Zapata V, De la Rúa P, Robledano F. Uncovering mechanisms of bird seed dispersal in semiarid environments to help to restore them. Ecosphere 2019;10(4):e02673.

82. Shiels AB, Walker LR. Bird perches increase forest seeds on Puerto Rican landslides. Restoration Ecology 2003;11(4):457-465.

83. Mesón M, Montoya M. Selvicultura Mediterránea. Madrid, Spain: Ediciones Mundi Prensa; 1993. 368 p.

84. Gómez-Aparicio L. The role of plant interactions in the restoration of degraded ecosystems: a meta-analysis across life-forms and ecosystems. Journal of Ecology 2009;97:1202-1214.

85. Jankju M. Role of nurse shrubs in restoration of an arid rangeland: Effects of microclimate on grass establishment. Journal of Arid Environments 2013;89:103-109.

86. Padilla FM, Pugnaire FI. The role of nurse plants in the restoration of degraded environments. Frontiers in Ecology and the Environment 2006;4(4):196-202.

Dear Dr. Karen Root,

We have now prepared a substantially revised version of our manuscript "**Unmasking the perching effect of the pioneer Mediterranean palm *Chamaerops humilis* L.**" (PONE-D-22-01669) submitted to *Plos One*.

The abandonment of traditional agriculture in most parts of Europe has brought challenge for the restoration of such human-disturbed habitats. Seed arrival is a crucial necessary event in the recolonization process that can be enhanced, by the so-called “perch plants”. Little is known yet about “perching” effect in most disturbed habitats needed of restoration actions. To evaluate for the first time several spatial aspects of the “perching effect” and whether the seed arrival via frugivorous bird is related to the spatial distribution of the perch plants, we used a spatially explicit approach in two disturbed sites (Matasgordas and Reserva) within the Doñana National Park (SW Spain). Specifically, we chose as study system the pioneer Mediterranean palm *Chamaerops humilis* L., which is often used as perch by a variety of frugivorous bird species. A total of 289 *C. humilis* were sampled (109 in Matasgordas and 180 in Reserva) in search of bird feces (N = 2998) and the dispersed seeds that they could have contained (N = 529). We detected a random spatial structure of bird feces and dispersed seeds in one study site, while a nonrandom spatial structure was found in in the second site, where isolated *C. humilis* showed a higher amount of bird feces and dispersed seeds than expected under spatial null models. The difference in spatial patterns between both study sites could be due, among other factors, to the different state of development in the ecological succession. The fact that the most visited *C. humilis* were isolated (rather than aggregated) questions the typical aggregated spatial structure of revegetation designs of many restoration projects. This novel study reveals novel spatial aspects of the “perching” effect and emphasize their importance, as well as that of *C. humilis*, in the restoration of human-disturbed habitats. Thus, our contribution will be of much interest for a large fraction of *Plos One* readership, and in particular to those interested on ecological interactions, plant dynamics and restoration.

We would like to thank you and the reviewers for your thoughtful comments, and we are happy with the careful and constructive criticisms and helpful suggestions. In response to the comments, we have introduced the following three main changes:

1. As suggested by the editor and both reviewers, the manuscript has been carefully revised several times to improve the English grammar and flow of logic.

2. Following suggestion by reviewer #1, the role of birds as frugivorous and seed dispersers have been described in further detail. Also, some spatial analyses have been repeated excluding data from *Rubus ulmifolius* and the result are now provided in a new appendix.

3. As suggested by reviewer #2, we have conducted cluster analyses to estimate the distribution of *C. humilis* in both study plots. Also, we fitted a new GLM to estimate the effect of the interaction between palm height and palm area on seed arrival to *C. humilis*. Results of these new analyses are provided in the corresponding new appendices.

Please, find below an itemized list detailing how we have responded to each of the items in the reports. The comments are in black font, our response follows in blue font, and changed phrases in response to the comment are provided in blue font and between quotation marks and.

Thank you again for your kind assistance and the time devoted to our manuscript.

Sincerely,

Jose M. Fedriani

1. Changes made to address editor comments:

The discussion, though, needs substantial revision to increase clarity and improve the interpretation of your results, especially in terms of language. It contains many awkwardly worded sentences that are challenging to understand (e.g., lines 459-462; 465-467; 493-497; 508-511; 550-552; 565-566; 594-596) and/or use poor grammar (e.g., lines 268-269; 502-503; 536-537; 559-561). Some of these issues could be minimized if you use more active rather than passive voice.

Thank you for your suggestions. To address them, we made the following changes:

Lines 495-499 (previously 459-462): "Considering that our sampling period did not take place throughout the year and that an unknown fraction of the bird feces presents in target *C. humilis* individuals surely went unnoticed, the data obtained could be considered as a hint of how widespread the "perching" effect in both study plots. Therefore, the high presence of (frugivorous) bird feces and dispersed seeds in both study plots is remarkable.".

Lines 501-504 (previously 465-467): "Nevertheless, we did not find seeds of the beneficiary species *C. salviifolius*, *P. bourgaeana*, *Q. suber* and *S. genistoides*, which are usually associated to *C. humilis*. Most of these plant species are seldom dispersed by birds".

Lines 516-519 (previously 493-497): "However, by concentrating frugivorous activity to aggregated planted trees, seed dispersal could be limited [16, 55]. Therefore, our results supporting the role of isolated perches diverge from the most widespread tendency in ecological restoration of aggregating trees and shrubs during revegetation efforts [65]".

Lines 523-526 (previously 508-511): "However, by concentrating frugivorous activity to aggregated planted trees, seed dispersal could be limited [16, 55]. Therefore, our results supporting the role of isolated perches diverge from the most widespread tendency in ecological restoration of aggregating trees and shrubs during revegetation efforts [65]".

Lines 583-587 (previously 550-552): "Additionally, beyond the fact of bearing fleshy fruits, the own beneficiary species could be used as a perch and thus attract birds. Therefore, a similar reasoning explained earlier about how *C. humilis* height influences seed dispersal, could be applied for the revealed positive effect of height of beneficiary plants".

Line 605-607: (previously 565-566): "In the last 30 years, restoration ecology has undergone considerable development thanks to the emerging new tools and techniques that facilitate the retrieval of biological properties of disturbed habitats [80]".

Lines 635-638 (previously 594-596): "We prove for the first time its key role as natural perch, an ecological function that, though it is shared with some fleshy-fruited woody plants, is especially relevant in early stages of ecological succession when few pioneer species occur".

Lines 288-289 (previously 268-269): "Finally, if km1. (r) ~ 1, indicates that the number of seeds is not affected by the distance to *C. humilis* individuals".

Lines 531-533 (previously 502-503): "Besides, it seems reasonable to expect that birds select *C. humilis* located on the edge of the plot, since these individuals are closer to the Mediterranean scrubland and thus allow birds to move between habitats with a minimal consumption of energy".

Previous lines 536-537: deleted.

Lines 598-600 (previously 559-561): "In summary, taller (and isolated) male *C. humilis* with a higher diversity of beneficiary species seemed to be the most suitable for attracting frugivorous birds and, therefore, received higher number of dispersed seeds".

In particular, it will be important to address the concerns of the both of the reviewers who highlight a number of areas that need further exploration. Reviewer #1 has some concerns about the foraging and movement ecology of the dispersers and the potential impact on the seed dispersal patterns as well as the large differences in the types of seeds potentially dispersed. Please also note that Reviewer #1 has some specific questions that should be addressed to improve clarity, particularly in the study design and motivations. Reviewer #2 also provides some valuable recommendations for each section and identifies a number of places where more explanation or revision is needed. Some additional issues are identified below:

Regarding Reviewer #1's concerns: we have improved our manuscript according to the comments received. We answered the questions related to the ecology of dispersers and did some new analyses without considering the *Rubus'* seeds. We found that the results obtained when we excluded these seeds barely differed from the results obtained when those seed were included.

In respect of Reviewer #2's comments, we have considerably improved the clarity and readability of the whole manuscript. We also added some information that Reviewer #2 considered important to clarify some aspects (*C. humilis* distribution, replicability, area of study, origin and identification of feces, etc.). We also run a new GLM including the interaction between *C. humilis* height (m) and area (m^2) instead of the variable volume (m^3), as suggested. The GLM obtained for both study plots (Matasgordas an Reserva) and for the three studied levels (presence of feces, presence of seeds and number of seeds) were pretty similar to the models obtained when the variable Volume was used. So, we provide the new model in the appendixes and kept the original one in the main text. We also carried out the analysis to study the distribution and level of aggregation of *C. humilis*. Our cluster analyses showed that Reserva has a greater percentage of isolated C. *humilis*, which is consistent with our results showing that birds prefer isolated *C. humilis* for perching in Reserva.

Line 63: "template" should be "temperate"

Thank you. Done.

Line 99: The use of the term "beneficiary" requires some type of description/definition or a reference source.

To address your comment have made the following change at Line 79-81: "Interestingly, in some cases, the same shrub species acting as perches also act as nurse plants, i.e. facilitating the emergence, growth and survival of other plant species, [23] which are designated as "beneficiary species", promoting the natural (re)colonization [24, 25]."

Also, at Line 220-222, we now state: "Beneficiary species were woody plants that grow under dwarf palms and emerge from the top of their surface. These species often benefit from C. *humilis* due to microclimatic improvement and protection against herbivory [42]."

What characteristics define this group and is it context-dependent?

Thank you for such an interesting point. In Garrote *et al* (2019), some of us proved that there is a consistent and strong positive spatial association between *C. humilis* and several woody species. This result suggested that the interaction is constant rather than context dependent. Most recently (Garrote *et al.* 2022), however, we have showed through field experiments that the strength and even the sign of such interaction changes mostly with plant stages (seed survival, seedling emergence and survival), which would suggest context-dependency. However, the overall effect *C. humilis* on woody species was always either positive or neutral and whenever we detected neutral effects that result seemed associated to limited sample sizes. So, most compelling evidence indicate that, in overall, *C. humilis* exerts a consistent positive effect on woody species.

Garrote PJ, AR Castilla, JM Fedriani. 2019. The endemic Mediterranean dwarf palm boosts old-field recolonization: implications for restoration. Journal of Environmental Management 250: 109478.

Garrote PJ, A Castilla, JM Fedriani. 2022. Coping with changing plant-plant interactions in restoration ecology: effect of species, site, and individual variation. Applied Vegetation Sciences. DOI: 10.1111/avsc.12644

Line 232: Why 199 simulations? It seems like a rather arbitrary number. Is this based on some initial modeling or preliminary results?

The 199 simulations are the number recommended by Wiegand & Moloney (2013). They estimate the curve asymptotes for each spatial statistic (rank, *P*-value, etc.).

Wiegand, T., & Moloney, K. A. (2013). Handbook of spatial point-pattern analysis in ecology. CRC press.

Line 255: “that” should be “than”

Thank you. Done.

Journal Requirements:

When submitting your revision, we need you to address these additional requirements.

1. Please ensure that your manuscript meets PLOS ONE's style requirements, including those for file naming.

Reviewed and ensured.

2. In your Methods section, please provide additional information regarding the permits you obtained for the work. Please ensure you have included the full name of the authority that approved the field site access and, if no permits were required, a brief statement explaining why.

We have added at Lines 131-132: “All permits necessary were granted by the National Park Service (ref. 2019/10) and the Junta de Andalucía (ref. 2019107300002261/IRM/MDCG/mes).”

3. Please note that in order to use the direct billing option the corresponding author must be affiliated with the chosen institute. Please either amend your manuscript to change the affiliation or corresponding author, or email us at plosone@plos.org with a request to remove this option.

Thank you. We have now specified JM Fedriani (who is affiliated at CSIC) as corresponding author in the Plos One manuscript submission platform. Also, in the manuscript both JM Fedriani and V Gonzalez-Garcia appear as corresponding authors.

4. We note that you have stated that you will provide repository information for your data at acceptance. Should your manuscript be accepted for publication, we will hold it until you provide the relevant accession numbers or DOIs necessary to access your data. If you wish to make changes to your Data Availability statement, please describe these changes in your cover letter and we will update your Data Availability statement to reflect the information you provide.

https://figshare.com/articles/dataset/PerchigEffectDwarfPalm_xlsx/19642446

5. We note that Figures 1 and 4 in your submission contain map images which may be copyrighted. All PLOS content is published under the Creative Commons Attribution License (CC BY 4.0), which means that the manuscript, images, and Supporting Information files will be freely available online, and any third party is permitted to access, download, copy, distribute, and use these materials in any way, even commercially, with proper attribution. For these reasons, we cannot publish previously copyrighted maps or satellite images created using proprietary data, such as Google software (Google Maps, Street View, and Earth). For more information, see our copyright guidelines: http://journals.plos.org/plosone/s/licenses-and-copyright.

Sorry, we are not sure to fully understand your concern. Figure 1 and Figure 4 represent study areas sketch maps and study plots, respectively. They have been made by us specifically for this manuscript. To this aim, we used QGIS v3.18 software. So, we don't see any copyright issue. However, we now provide the photo credit in Figure 2 and Figure 3 headings (Photo credit: Pedro J. Garrote) which was missing in the earlier manuscript version.

2. Changes made to address reviewer #1 comments:

Although there is a lot of consideration on the use of artificial perches in restoration, the role of natural perches deserves more investigation, especially in a poorly studied biome under this perspective, as is the case with Mediterranean Europe. In this regard, the authors provide an important dataset based on field observations and fine spatial analyses, which represents a great contribution to the restoration of that particular habitat.

Thank you very much for your suggestions and comments to improve our manuscript.

Despite their efforts to explain the different patterns found between the two study sites (Mastasgordas and Reserva), I think this attempt was a bit confused, perhaps because there may be underlying causes not fully investigated by the authors. I would put this question in the perspective of the field data and not in the analysis itself. One underlying problem may be the unbalanced frequency of birds and seeds in the two areas. From the nine bird species recorded, six are reputed insectivores and just three can be considered omnivores that can take significant amounts of fruit in their diets (https://esajournals.onlinelibrary.wiley.com/doi/abs/10.1890/13-1917.1). These three omnivore species performed more than 50% of the visits recorded in the two areas and probably deposited more seeds per visit beneath the palms than the other ones. Special attention should be paid to the foraging and movement patterns of these three omnivores, since they may account for the general pattern of the seed rain and, perhaps, provide sound explanations for these patterns.

Lines 190-193: "Overall, nine bird species were recorded perching: *Cisticola juncidis*, *Lanius senator*, *Phoenicurus ochruros, Phylloscopus collybita, Pica pica, Saxicola rubicola, Sturnus unicolor, Sylvia atricapilla* and *Sylvia melanocephala"*.

All species but *Cisticola*, *Lanius*, *Pica* and *Sturnus* are reported by Herrera (1984) as frugivorous. However, *Pica pica* does include fleshy-fruits (Green *et al*. 2019) and acorns (Martínez-Baroja 2019) in their diet. Additionaly, genus *Sturnus* has been reported as fruit-eater too (Jordano 1987, Gonzalez-Varo et al. 2017), as well as genus *Phoenicurus*, *Sylvia* and *Saxicola* (Campo-Celada *et al*. 2022, Carnicer *et al*. 2009). Therefore, just two of our reported species (*Cisticola juncidis* and *Lanius senator*) would not participate directly in seed dispersal.

That is a good point even though during the fall and early winter (when our study was carried out) most of the recorded bird species (Table 1) shift their diet, at least partially, towards a frugivorous diet. To address your concern, we have added the following text in the discussion (Line 562-570): "However, we sampled during the peak of the seed dispersal season, time when some birds change their diet into a more fruit-based one. For instance, it has been reported that P. ochruros, S. atricapilla and S. malenocephala are able to quickly change their diet when fleshy fruits become available, while other species such as S. rubicola show a more gradual change to a frugivorous diet [67, 68]. Also, S. unicolor, P. ochruros, P. collybita, P. pica, and S. rubicola has been reported as fruit-eater, although in lesser quantities [69, 70, 71, 72, 73]. Therefore, although not all recorded bird species contribute equally to seed dispersal, fleshy fruits represent a considerable fraction of most of their diets during the seed dispersal season."

Campo-Celada M, Jordano P, Benítez-López A, Gutiérrez-Expósito C, Rabadán-González J, Mendoza I. 2022. Assessing short and long-term variations in diversity, timing and body condition of frugivorous birds. Oikos, 2: e08387

Carnicer J, Jordano P, Melían CJ. 2009. The temporal dynamics of resource use by frugivorous birds: a network approach. Ecology, 90(7): 1958-1970.

Gonzalez-Varo JP, Carvalho CS, Arroyo JM, Jordano P. 2017. Unravelling seed dispersal through fragmented landscapes: Frugivore species operate unevenly as mobile links. Molecular Ecology, 26(16): 4309-4321.

Green AJ, Elmberg J, Lovas-Kiss A. 2019. Beyond Scatter-Hoarding and Frugivory: European Corvids as Overlooked Vectors for a Broad Range of Plants. Front. Ecol. Evol., 18.

Herrera CM. 1987. A study of avian frugivores, bird-dispersed plants, and their interaction in Mediterranean scrublands. Ecological Monographs, 54(1): 1-23

Martínez-Baroja L, Pérez-Camacho L, Villar-Salvador P, Rebollo S, Quiles P, Gómez-Sánchez D, Molina-Morales M, Leverkus AB, Castro J, Rey-Benayas JM. 2019. Massive and effective acorn dispersal into agroforestry systems by an overlooked vector, the Eurasian magpie (*Pica pica*). Ecosphere, 10(12): e02989.

Another point addresses the plant composition and fruit types between the two areas. *Rubus ulmifolius* is the only recorded fruit consumed that is a functional berry, containing dozens of seeds. The other plant species are single-seeded drupes. This species was not related as common or abundant in Matasgordas, whose seed rain was nil, but its presence was highlighted in Reserva, whose seed rain under *C. humilis* accounted for 85-90% of the total seed rain. Even a few feces with dozen of *R. ulmifolius* seeds would certainly bias the results and the comparison between areas when the response variable "number of seeds" is considered. Would the spatial patterns be the same between the areas if *R. ulmifolius* is excluded from the analysis? If so, the conclusions presented by the authors should be revisited and adjusted to the bias introduced by this species.

I see these points do not invalidate the importance of *C. humilis* as perches and facilitative species in that habitat, but at least would provide more explanatory power for the distinct spatial patterns found between the two study sites.

Thank you for your suggestion. We repeated our spatial analysis for the presence of seeds and for the number of seeds excluding *Rubus*' seeds. The results obtained are pretty similar to the ones obtained initially when we included those seeds in the analysis. The results from such a new analysis are now included as Supporting Information (S2 Spatial seed analysis excluding *Rubus*' seeds).

Besides that, I would recommend the authors to improve the English text, as some sentences are somewhat difficult to understand.

Thank you. We have carefully revised the manuscript several times to improve it language use and clarity. The revised manuscript has considerably improved in this respect.

Bellow, I add some other specific points to the authors.

Page 4, lines 94-96: To question whether there are differences in the role of *C. humilis* as perches between the two areas presupposes some previous knowledge of the distributional pattern of this species between the areas. Although the authors provide a detailed description of the study areas in the proper session (pages 6 and 7) it should be enlightening to give a hint here on what triggered the idea that the plant role could be different between areas. Certainly, these two areas were not chosen at random.

Garrote *et al.* 2019 documented that Reserva presents higher density of shrubs than our study plot at Matasgordas. Such a difference in shrub cover led to a greater abundance of fleshy fruits in Reserva which likely makes this site more attractive to frugivorous birds.

Lines 137-138: "Study areas are different in terms of vegetation and human-use history [4, 38]".

Lines 147-152: "Due to such past human activity, Matasgordas is now formed, mainly, by two habitats: (i) Scrubland dominated by P. lentiscus shrubs with some *Q. suber* and *O. europaea* var. *sylvestris* trees [4, 33] and (ii) an old-field, where our study plot was set, which woody vegetation is mainly composed by animal-dispersed native plants such as *C. humilis*, P. *lentiscus*, *D. gnidium* and *P. bourgaeana*, *Asparagus aphyllus* L., *Halimium halimifolium* (L.) Willk and *Cistus salviifolius* L. and scattered *Q. suber* and *O. europaea* var. *sylvestris* trees [4]".

Lines 167-175: "Reserva (…) has been historically managed by human for agriculture, hunting, cattle grazing and tree felling, especially O. europaea var. sylvestris and *Q. suber* [41]. Reserva was protected earlier than Matasgordas, in 1964 and it has been recovering ever since, being recolonised by animal-dispersed plants such as *A. aphyllus*, *C. humilis*, *Phillyrea angustifolia* L. or *R. ulmifolius* [4]. This scrubland is dominated by *H. halimifolium* and *Stauracanthus genistoides* (Brot.) Samp. with scattered trees of *Q. suber*, *O. europaea* var. *sylvestris* and *Pinus pinea* [33]. The study plot at Reserva presents higher density of shrubs than the one at Matasgordas. Such a difference in shrub cover led to a greater abundance of fleshy fruits in Reserva, which likely makes this site more attractive to frugivorous birds [4].".

Page 6, line 142: Delete the period after bourgaeana.

Thank you. Done.

Page 7, lines 177-178: What was the total sampling effort for bird perching for the separate areas?

Thank you for your comment. To address it, we have added the following text (Line 189-190) in the revised manuscript: "During the sampling, which lasted about 3h per day in Matasgordas and about 2h in Reserva, any observed bird perching on focal *C. humilis* individuals was recorded."

Page 9, lines 189-192: What do you mean by "palm interior"? Does it include the foliage surface in the interior and also the soil under the foliage? Was the open area of a similar surface associated with the palms bare soil?

Palm interior refers to the foliage surface since the soil under the palm was unreachable in most of the cases. Thus, we have replaced in the manuscript "palm interior" for "palm foliage surface".

Yes, the open area associated to each palm was of similar surface (than the associated palm) and mostly in bare soil with scarce vegetation, mainly grasses. This open area was 3-4m from the associated *C. humilis*.

Lines 200-204: "Every target *C. humilis* was checked for bird feces and regurgitations by carefully checking the palm foliage surface. We also evaluated whether seeds arrive at open spaces without palms. To this aim, we checked for the presence of bird feces and regurgitations in an open area associated to each focal *C. humilis*. These areas were of similar surface and mostly located in bare soils with scarce vegetation (mainly grasses) and 3-4m from the associated C. *humilis*".

What were the criteria used to distinguish between bird-and-mammal dispersed seeds in these areas?

Most sampled seeds were found within bird feces which are easily distinguishable from mammal feces based on size, shape, color and smell. For example, the uric acid in bird feces forms a conspicuous and white sticky paste that makes them easily to identify. Also, the diameter of feces of target birds (a few millimeters) was much smaller than for feces of local frugivorous mammal species (several centimeters).

Page 14, lines 312-313: Since the number of feces and number of seeds are two interrelated response variables, what is the weight of the regurgitated seeds in the analysis? How much regurgitated seeds contributed to the total number of seeds sampled?

Regurgitated seeds were just a small fraction of the total sample. Their number was too small to really contribute to the final analysis.

Page 16, line 375: Consider this writing: "Otherwise, in Reserva, the probability …"

Thank you. We have changed the sentence. Line 395-397 (previously line 375): "Results in Reserva substantially differed from results from Matasgordas. The probability of two *C. humilis* separated by distance r, having both seeds (p11(r) statistic), was similar to expected of random labelling for all spatial scales (Fig 6B)."

Page 2, line 501: Insert "of" after "chances".

Thank you. Done.

3. Changes incorporated to address reviewer #2 comments:

Unmasking the perching effect of the pioneer Mediterranean palm Chamaerops humilis L.

I have now carefully read the manuscript. I found the authors approach very interesting using SPPA to

unravel the perching effect. Using this spatial analysis they evaluate several spatial patterns of “perching” effect and whether the seed arrival via frugivorous birds is related to the spatial distribution of the C. humilis in two disturbed scenarios. I think the manuscript could be interesting will be interesting for readers interested in restoration or ecological succession drivers.

Thank you very much for your suggestions and comment to improve our manuscript.

I think the manuscript needs to improve the readability mostly in the methods and the results section (see comments below).

We have considerably improved the clarity and readability of the whole manuscript, including the methods and results sections.

I have concerns with the general work. On one hand, I think that it needs more information about the state of the selected plots. For instance, the main objective is to evaluate the perching effect based on the plants distribution, however, the authors did not show the spatial distribution of the individual plants, plants density, size of the plot, etc. that in my opinion has to be in the article.

Thank you for communicating this concern. Both Figure 1 and Figure 4 show the spatial distribution of the individual *C. humilis*. Following your suggestion, plant densities and plot sizes are now included in the Material and Methods section:

Line 136-137: “We selected two study plots 10 km apart called Matasgordas (area sampled: 13.9 ha) and Doñana Biological Reserve (area sampled: 21.4 ha)”.

Line 182-184: “The density of *C. humilis* plants in Matasgordas’ plot (7.78 individuals/ha) was pretty similar than in Reserva’ plot (8.41 individuals/ha).”.

On the other hand, your main conclusions are based on some patterns found in one plot (male plants, isolation). This is my major concern because when you present the analysis of the other plot the main patterns did not applied to other ecological context. It seems that the results only applied under certain circumstances. I think this is a very common problem when studying the effects of perching which makes it very difficult to generalize the drivers of this process.

We agree in that is very difficult to generalize the drivers of the perching effect. Differences in results between our study areas likely relate to the fact that they present a different state of ecological succession, an issue that is detailed in our Discussion and could be used by managers to decide, for example, whether particular areas are adequate to promote seed arrival using natural or artificial perches.

Abstract

L39- “dispersed seeds We detected” missed a point

Thank you. Done.

Introduction

L97: Do exist some individuals of C. humilis that act as “hotspots” of dispersed seeds? Change for: Do any C. humilis individuals act as "hot spots" for seed dispersal?

Changed for: Do particular *C. humilis* individuals act as "hot spots" of seed arrival, and if so, why? (Lines 99-100, previously Line 97).

Methods

L134-144. With this explanation it is unclear where did you work. Reading some works made in the area it seems that Matasgordas has a higher plant density than Reserva. For instance, In the article by Fedriani and Delibes (2011) when the description of the Matasgordas site is made, it seems to have a higher density of individuals, same situation in the article by Jácome et al. 2016 where the spatial patterns of this species are studied where they also report higher number of individuals in the area. Thus, it would be interesting to specify that you do it "in a low density area in order to be comparable with Reserva."

Thank you for your comment. The density for *C. humilis* is highly spatially heterogeneous both in Matasgordas and in Reserva areas. Thus, it is expectable that density changes depending on the specific location of the study plots in both areas. In our selected study plots, the density of *C. humilis* plants in Matasgordas (7.78 individuals/ha) was pretty similar to that in Reserva (8.41 individuals/ha) but, again, this could change if the plots were set in different specific locations. Ultimately, Matasgordas and Reserva were not too different in terms of palm density.

Our previous studies (Fedriani and Delibes 2011, Jácome et al. 2016) were carried out in Matasgordas and Reserva but their study plots were not the same that the pots of the present study; so, no wonder they reported other *C. humilis* densities. For instance, the study by Fedriani and Delibes (2011) was carried out in a patch of Mediterranean scrubland within Matasgordas area (where *C. humilis* density is certainly very high), whereas our present study was conducted in the old-field within Matasgordas area (where *C. humilis* density is much lower than in the scrubland).

To prevent possible misunderstandings in relation to this issue, we now consistently use throughout our manuscript the term study plot instead of study site.

L163- Related with my previous comment. Although Reserva was protected earlier authors have to consider that there still traditional uses in the area, thus plants in the Reserva are submitted to a high herbivorous pressure due to the presence of horses, cattle and high density of cervids. This pressure has reflected in the lower density of individuals explained above.

As mentioned above, the densities of *C. humilis* in both Matasgordas (7.78 ind. ha^{-1}) and Reserva (8.41 ind. ha^{-1}) were not too different but, they could change depending on where the study plots are located.

Suggested papers:
-Fedriani, J. M., & Delibes, M. (2011). Dangerous liaisons disperse the Mediterranean dwarf palm: Fleshy-pulp defensive role against seed predators. Ecology, 92, 304–315.
-Jácome-Flores, M. E., Delibes, M., Wiegand, T. & Fedriani, J. M. Spatial patterns of an endemic Mediterranean palm recolonizing old fields. Ecol. Evol. 6, 8556–8568 (2016).

L170-Every target C. humilis individual (109 and 180 in Matasgordas and Reserva, respectively)

Thank you. Done.

SPPA requires the delimitation of an area in which all target individuals of the study species are georeferenced. I suppose this was the case, however it is not clear in the methodology. I suggest to put more information such as density and/or no. of individuals, area sampled, etc. for each selected plot. Also this information will clarify more the differences that you found between plots.

Thank you. The study plots are shown in Fig. 1. We have added the suggested information:

Lines 136-137: “We selected two study plots 10 km apart called Matasgordas (area sampled: 13.9 ha) and Doñana Biological Reserve (area sampled: 21.4 ha)”.

Lines 181-184: “Every target *C. humilis* (109 and 180 in Matasgordas and Reserva, respectively) was individually georeferenced with a submetric GPS Leica 1200. The density of *C. humilis* plants in Matasgordas’ plot (7.78 individuals/ha) was pretty similar than in Reserva’ plot (8.41 individuals/ha)”.

Discussion

L463-L478- Reduce the length of this paragraph, there is too much mention of dispersal mechanisms of other species. This section should only focus on the ability of C. humilis to attract different dispersers including mammals.

We changed the paragraph to highlight the *C. humilis* ability to attract seed dispersers as you suggest.

Lines 500-509: “Besides, it is apparent that some seeds can emerge seedling beneath *C. humilis*, since all seeds collected belonged to beneficiary woody species found within *C. humilis*. Nevertheless, we did not find seeds of the beneficiary species *C. salviifolius*, *P. bourgaeana*, *Q. suber* and *S. genistoides*, which are usually associated to *C. humilis*. Most of these plant species are seldom dispersed by birds. Other animals are more likely to disperse them, such as insects for *C. salviifolius* or *S. genistoides* seeds [56, 57, 58] or mammals, such as ungulates (red deer, wild boars, domestic cattle) and carnivores (Eurasias badger, red fox) for *P. bourgaeana* seeds [59]. Therefore, the presence of these woody species associated to *C. humilis* reveals that it does not attract just bird-dispersed plants but also mammal-dispersed plants, accentuating its role in habitat restoration.”

L484-485 I suggest to the authors to do SPPA to the individual plants to unravel the spatial patterns of the “Matasgordas and Reserva C. humilis populations”, this analysis could be useful to explain some of the patterns. For instance maybe the patterns of the plants in Matasgordas tend to be more random explaining your findings without any over explanation. On the other hand maybe Reserva population its characterized by more isolated plants that had enough height to function as a perch for birds.

Thanks for your suggestion. We carried out some cluster analysis in order to quantify the *C. humilis* distribution and level of aggregation. To evaluate the fit of the observed point patterns in both Matasgordas and Reserva to different cluster processes, four different summary functions were used: pair correlation function g(r), L-function L(r), Spherical contact distribution $H_S(r)$ and nearest neighbor distribution function D(r).

In both study plots, the four summary functions showed good fits with the selected null models, being the model-predicted values mostly confined within the simulation envelopes. The distribution of *C. humilis* in Matasgordas and Reserva were both best described by a double-clustered component pattern with a random component pattern, being Reserva the one with a greater proportion of isolated *C. humilis* individuals. This may relate to the observed tendency found in Reserva of bird’s preference for isolated *C. humilis*, which is now mentioned in the Discussion.

We added the results of cluster analyses as Supporting Information (S1 Cluster Analysis) and are quoted in the description of the study plots.

L528-529: …while the surface of Chamaerops humilis was found significant in Reserva
I suggest to change surface to cover area.

Thank you for your suggestion. We have removed that line in the revised version.

L526-534. Sometimes is difficult and confusing to follow the explanations or results because sometimes appear first Matasgordas and then Reserva. I suggest to the authors to be consistent with the appearance order through all document.

Thank you. The order is now consistent, as suggested.

L550 Change: What is more to Furthermore or Additionally

Thank you. Done.

L552- Unexpectedly, it was found that larger C. humilis (i.e. those with larger volume) were less suitable for seed arrival, at least in Reserva. -Change “it was found” to “we found”

Thank you for your suggestion. We have removed that line in the revised version.

-I suggest to the authors not use volume but instead the interaction between cover area (surface) and height. This interaction could reveal some perching preferences regarding this two variables. The variety of shapes in this palm make it difficult to standardize the volume as a “common variable” between all individuals.

Although this species can be highly variable in shape, we found that *C. humilis* in our study plots were very similar in shape, with a consistent dome shape. This uniformity made us to choose the variable ‘volume’.

However, following your suggestion, we carried out the GLM with the interaction between Height (m) and Area (m^2) instead of the variable Volume (m^3) and the results obtained were pretty similar to the ones obtained in the initial GLM. Results from this new analysis are shown in the Supporting Information (S3 Effect of palm traits).

L554-We speculate that male C. humilis could be selected over females because they lack fruits. C. humilis fruits are too big for birds, but they are often eaten by medium-sized carnivores such as the European badger and the red fox [33]. The presence of these predators could dissuade frugivorous birds from visit female C. humilis in fruit, and thus perching more often male palms.
I suggest to put some information about the abundance of “potential predators” in each study area to reinforced this idea. Another explanation to this pattern (in my opinion more likely) is the presence of rodents and rabbits beneath the female plants. Rodents and rabbits are also highly attracted to C. humilis fruits, in this foraging process they could predate the seeds dropped in the perching process, limiting the number of seeds. In this context, the same situation could be happening in aggregated C. humilis individuals that are preferred by rodents due to high vegetation cover.

Thank you for your comment. On the one hand, the presence and abundance of potential predators in this area, such as red foxes and badgers, have been studied earlier (Fedriani et al. 1998, Fedriani et al. 2008). On the other hand, all seeds collected were found at a certain height at the palm leaves, being pretty much inaccessible for those mammals (especially rabbits). Further, during our intensive monitoring of *C. humilis* using camera traps we have not recorded ever rodent foraging on leaves at the top surface of this palm, where seeds dispersed by birds were collected.

L564- It seems that your results could be useful for restoration planification where the C. humilis is a very useful species. However the inconsistency of the results between areas and the unclear relation between the traits masked by the context dependency seems to be an issue to the applicability of the study. For instance the efficiency of the perching effect seems to be related to male plants, if we use this results as

it comes we would be deprecated female plants that would be useful to attract super-efficient fleshy fruit dispersers such as badgers or foxes.

Thank you for your comment. We have included the following sentences in the revised manuscript (Lines 593-597): "The use of *C. humilis* in ecological restoration should follow a balance between male and female plants. Male plants would attract preferably birds that would disperse seeds of woody species present in the surrounding area, while female plants would attract medium-sized carnivores and ungulates that would mostly disperse seed of species such as *P. bourgaena* and C*. humilis*."
