# Supplementary material for: Unmasking the perching effect of the pioneer Mediterranean dwarf palm Chamaerops humilis L
Source: PLoS One. 2022 Aug 23;17(8):e0273311. doi: 10.1371/journal.pone.0273311 (PMC9398033; doi:10.1371/journal.pone.0273311)
Supplement: Attachment — Submitted filename: Response to Reviewers (2).docx [file pone.0273311.s011.docx]

**RESPONSE TO EDITOR COMMENTS**

I appreciate the authors’ thoroughness and thoughtfulness in addressing the numerous comments and suggestions by the original reviewers. The paper examines an interesting restoration question about the role of perch plants for seed dispersal in disturbed environments. While it makes an important and timely contribution to our understanding of the effects of structure on seed dispersal the manuscript needed increased detail, clarity and improvement in the description of the study design, the analysis and the interpretation of the results. The revised version is much clearer and the results are much better supported. The new analyses are illuminating and provide important detail to the results. As the reviewers suggest there are still a few minor revisions that could further strengthen the paper and improve the readability. Please note, though, the reviewers for this version are not the original reviewers so they have provided a fresh perspective on the revised manuscript. As Reviewers 3 and 4 highlight, there are a number of places where some additional editing (e.g., improving the grammar) will improve the readability. As reviewer 3 suggests, it would also be help to refer to the sites in a consistent order to aid the reader in tracking the differences. Both reviewers have included detailed suggestions and clarifying language that should be considered throughout, which are included in the annotated versions. In addition, they have provided questions highlighting places where additional information or a rephrasing may be necessary. With these minor revisions, the paper should be well crafted to contribute to our understanding of the ecological implications of structure in restoration on seed dispersal.

-->Thank you very much for your positive comments. We have carefully followed comments by reviewer 3 and 4 to improve further the grammar and readability of our manuscript. We now refer to the study sites in a consistent order to aid the reader in tracking the differences. Also, we have included additional information or a rephrasing when necessary.

**RESPONSE TO REVIEWER 3 COMMENTS**

Reviewer #3: A note that I was not a reviewer on the first round of the manuscript. I thought for the most part that the authors did a thorough job of responding to prior reviewers’ comments. Generally, the paper is clearly written and the analyses seemed sound. I did, however, make quite a few edits directly on the manuscript and think the manuscript needs another round of revisions. Here I just summarize a few key points.

-->Thank you very much for your helpful comments.

1. Most of my comments are grammatical corrections (using either comments or in red text). I started doing a thorough edit but that is challenging with a pdf so only did so on the first couple of pages. The paper needs a thorough edit by somebody with a strong command of English grammar.

-->We have addressed all grammatical corrections pointed by both reviewers. We have also revised again the whole manuscript ton improve its clarity and flow of logic.

2. The authors refer to their two sites by the actual site names, but those site names are meaningless to the reader. Then the authors have to reminder the different characteristics of the sites. I suggest that the authors name the sites for the paper using what they think is the most distinguishing feature – e.g. early-successional and late-successional site.

-->The names of both study sites have been changes as suggested throughout the manuscript.

3. The authors overstep their data in their restoration recommendations. They have data from only two sites and the results differ in the two sites, which is typical of restoration – results are often site-specific. So they have a limited ability to generalize. They also make recommendations in that section that don’t stem from the data presented. I strongly recommend that they combine their “recommendations” and “conclusions” section into one section and condense the restoration conclusions to 1 paragraph.

-->All these suggestions have been included in the revised manuscript.

4. The authors frequently put modifying clauses in parentheses. In most cases, these should be part of the main sentence.

-->We have included in the main sentences several clauses that used to be in parentheses.

Please see the manuscript for additional comments and edits.

-->We have addressed all comments and edits provided in the annotated manuscript. Below, I summarize the most relevant ones:

L472. How can height be negatively relate to number of seeds and size positively related?

-->There was a mistake in this sentence. Both height and area negatively relate to number of seeds, whereas volume was positively related. We have corrected these sentences.

L517-518. Or is it that the feces were more concentrated in single plants because there was only one?

-->Indeed, in late successional study plot (Reserva) there were more individuals that receive seeds (31) than in the early successional study plot (Matasgordas; 20).

**RESPONSE TO REVIEWER 4 COMMENTS**

Reviewer #4: I made a series of minor suggestions/comments directly on the ms.

-->Thank you for your comments and suggestions. All of them have been addressed in the revised manuscript.

Regarding the availability of data, I found a vague statement that "all data are fully available without restriction", without any indication of how to access it.

-->The data used for this study is fully available at:

https://figshare.com/articles/dataset/PerchigEffectDwarfPalm_xlsx/19642446
